# Development and feasibility testing of the taVNS adverse events questionnaire: a multistage methodological study

**DOI:** 10.3389/fnins.2026.1974496

**Published:** 2026-10-28

**Authors:** Katharina Meier-Bartelt, Luisa M. Schmidt, Till Bömmer, Karsten Witt

**Affiliations:** 1 Department of Neurology, Carl von Ossietzky University, Oldenburg, Germany; 2 University Clinic for Neurology at the Evangelical Hospital, Oldenburg, Germany; 3 Research Center Neurosensory Science, Carl von Ossietzky University, Oldenburg, Germany

**Keywords:** adverse events, questionnaire, targeted literature review, taVNS, transcutaneous auricular vagus nerve stimulation, safety, neuromodulation, methodological study

## Abstract

Transcutaneous auricular vagus nerve stimulation (taVNS) is a non-invasive neuromodulatory therapy gaining interest for its neural mechanisms and therapeutic effects. Although taVNS is considered safe, adverse event (AE) reporting remains inconsistent, lacking standardized procedures for assessment and documentation. This study aimed to develop a new instrument and conduct a preliminary evaluation of its use for systematic and structured assessment and reporting of AEs associated with taVNS. To achieve this objective, a multistage development process was undertaken, comprising: item generation, cognitive pretesting, pilot testing and translation into several languages. A targeted literature review of taVNS studies was performed to identify and categorize previously reported AEs and to form item generation. Based on these findings, a semi-structured taVNS Adverse Events Questionnaire (taVNS-AEQ) was developed. Its feasibility and usability were evaluated through cognitive pretesting and pilot testing in a randomized crossover study comparing active and sham taVNS. The literature search initially identified 203 records, of which 71 studies met the predefined eligibility criteria and were included in this review. Among these 71 studies, only 39 provided semi-structured AE data. A total of 539 individual AE symptoms were reported as absolute counts (19 studies), complemented by symptom data assessed using Likert-scales (20 studies), and were categorized for analysis and item generation. In a real-sham crossover study with 29 participants, the most frequent AEs were tingling, fatigue, and changes in concentration. The questionnaire demonstrated good feasibility, and after revisions version 3.0 was translated into four languages. The newly developed semi-structured taVNS-AEQ 3.0 includes four participant items, seven personnel items, and recommendations for documenting stimulation parameters. Current evidence on taVNS-related AEs is limited by the heterogeneous nature of the reporting. The taVNS-AEQ offers a newly developed semi-structured approach to AE assessment, potentially enhancing the quality and comparability of future taVNS studies.Systematic review registration: PROSPERO (Registration Number: CRD420251208237).

## 1 Introduction

Transcutaneous auricular vagus nerve stimulation (taVNS) is a non-invasive vagus neuromodulation technique that stimulates the auricular branch of the vagus nerve via an ear electrode. It leverages the broad innervation of the vagus nerve and its modulatory influence on diverse physiological functions (Yuan and Silberstein, 2016a). As a non-invasive nerve stimulation, taVNS has gained increasing attention in basic research and clinical applications. It received European clearance for the treatment of epilepsy and depression (2010) and chronic pain (2012) (Howland, 2014). Demonstrating its tolerability is essential for research and therapeutic applications. Although taVNS aims to induce neuromodulatory effects, it may also elicit adverse events (AEs), which are defined as unfavourable or harmful outcomes occurring during or after an intervention (Higgins and Altman, 2008). To date, no standardized questionnaire that had undergone different stages of development has comprehensively captured taVNS-related AEs. An ideal instrument would cover the full spectrum of known and rare AEs, ensure comparability across studies and stimulation parameters, and improve user expectations and treatment adherence through systematic reporting of AEs.

Given its extensive innervation, the vagus nerve influences the cardiac, respiratory, gastrointestinal, immunologic, somatosensory, and endocrine systems (Howland, 2014; Matsuoka et al., 2025). Therefore, vagus nerve stimulation (VNS) may affect multiple organ systems and produce AEs across domains. Individual factors, such as disease conditions, sensitivity, and anatomical variants, may further shape the AE profile. While invasive VNS is associated with more severe AEs (e.g., bradycardia, dyspnoea, surgical trauma), taVNS-related AEs are generally mild (Yuan and Silberstein, 2016b; Huffman et al., 2021; McGhee et al., 2023). Common AEs include transient local skin irritation, ear discomfort, neck pain, headache, and nausea (Redgrave et al., 2018; Kim et al., 2022). However, these AEs were not always compared with the AEs of sham stimulation. Unintentional stimulation of adjacent nerve fibres may also occur, which could potentially cause peripheral facial paralysis, tongue, and pharyngeal effects (Kiyokawa et al., 2014).

The objective of this study was to develop and conduct preliminary feasibility testing of a newly developed semi-structured questionnaire for the assessment and reporting of AEs associated with taVNS. As part of a multistage development process, a targeted literature search was performed to obtain a comprehensive overview of the AEs reported in taVNS studies and to generate questionnaire items. Based on the identified AE spectrum, the taVNS-AEQ was developed to capture potentially unknown or very rare AEs and enable consistent AE recording across stimulation parameters, experimental settings, and populations. Subsequently, taVNS-AEQ 1.0 was evaluated through cognitive pretesting, version 2.0 through pilot testing in a sham-controlled taVNS feasibility study and after that version 3.0 translated into several languages to facilitate its broader application.

The overarching aim was to create an easy-to-use instrument that supports evidence-based statements on taVNS-related AEs in future and enhances future safety standards.

## 2 Methods

### 2.1 Item generation

First, a targeted literature search was conducted including studies of healthy participants and clinical patient groups, to find a broad spectrum of relevant symptoms and characterize AEs for item generation. Inclusion and exclusion criteria of the items were determined by the research team. In result, the initial item pool was selected and the first version of the taVNS-AEQ was developed.

Second, the taVNS-AEQ 1.0 was tested through cognitive pretesting with 12 healthy participants. After revisions, version 2.0 of the taVNS-AEQ was created.

Third, the questionnaire was further evaluated to check for plausibility and feasibility in form of pilot testing in a cross-over real versus sham taVNS study with 29 healthy young participants.

Fourth, after further revisions, the newly developed semi-structured taVNS-AEQ version 3.0 was translated into four languages and linguistically reviewed by native speakers.

In this study, a symptom is defined as a sensory, cognitive, physical or perceived change of subjective sensation. This term does not imply that a symptom is pathological or treatment-related to taVNS.

An adverse event is defined as unfavourable or harmful outcome or unintended symptom occurring during or after stimulation, irrespective of an existing causal relationship with taVNS.

AE severity refers to its intensity and is commonly categorized as mild, moderate or severe. An AE is considered as serious based on additional clinical consequences, for example hospitalization, life-threatening event, medical intervention or persistent disability (ICH, 1995).

### 2.2 Targeted literature review of AEs in taVNS

A literature review was conducted to identify and characterize AEs and concepts relevant to the development of the preliminary AE item generation. Studies conducted in both healthy and clinical patient populations were considered in order to obtain a broad overview of AE concepts reported across different populations.

The search and study selection process was transparently documented and structured by relevant elements of the Preferred Reporting Items for Systematic Reviews and Meta-Analyses (PRISMA) framework (Page et al., 2021), including extensions for literature search (Rethlefsen et al., 2021) and AE reporting (Zorzela et al., 2016), as well as recommendations from the Cochrane Adverse Effects Methods Group (Peryer et al., 2024; Loke et al., 2007), although a target literature and no systematic review was conducted. The protocol was preregistered with PROSPERO (Registration Number: CRD420251208237).

#### 2.2.1 Search strategy

The targeted literature search was conducted in PubMed and Scopus covered publications available up to May 17, 2025, using the terms “taVNS,” “atVNS,” “tVNS,” “transcutaneous auricular vagus nerve stimulation,” “auricular transcutaneous vagus nerve stimulation,” “transcutaneous vagus nerve stimulation,” “non-invasive vagus nerve stimulation,” and “non-invasive VNS.”

#### 2.2.2 Selection criteria

Three researchers independently screened the titles and abstracts for eligibility criteria. Studies were included if they met the following PICOT criteria:

- P: human participants
- I: taVNS intervention
- C: real stimulation with or without sham control
- O: any reported outcome
- T: AEs occurring during or after stimulation

The exclusion criteria were non-human studies, absence of AE reporting, reviews, conference abstracts, preprints, letters without data, stimulation sites outside the ear, and combined stimulation of additional nerves. A flow diagram (adopted by PRISMA) summarizes the selection process (Figure 1).

**Figure 1:**
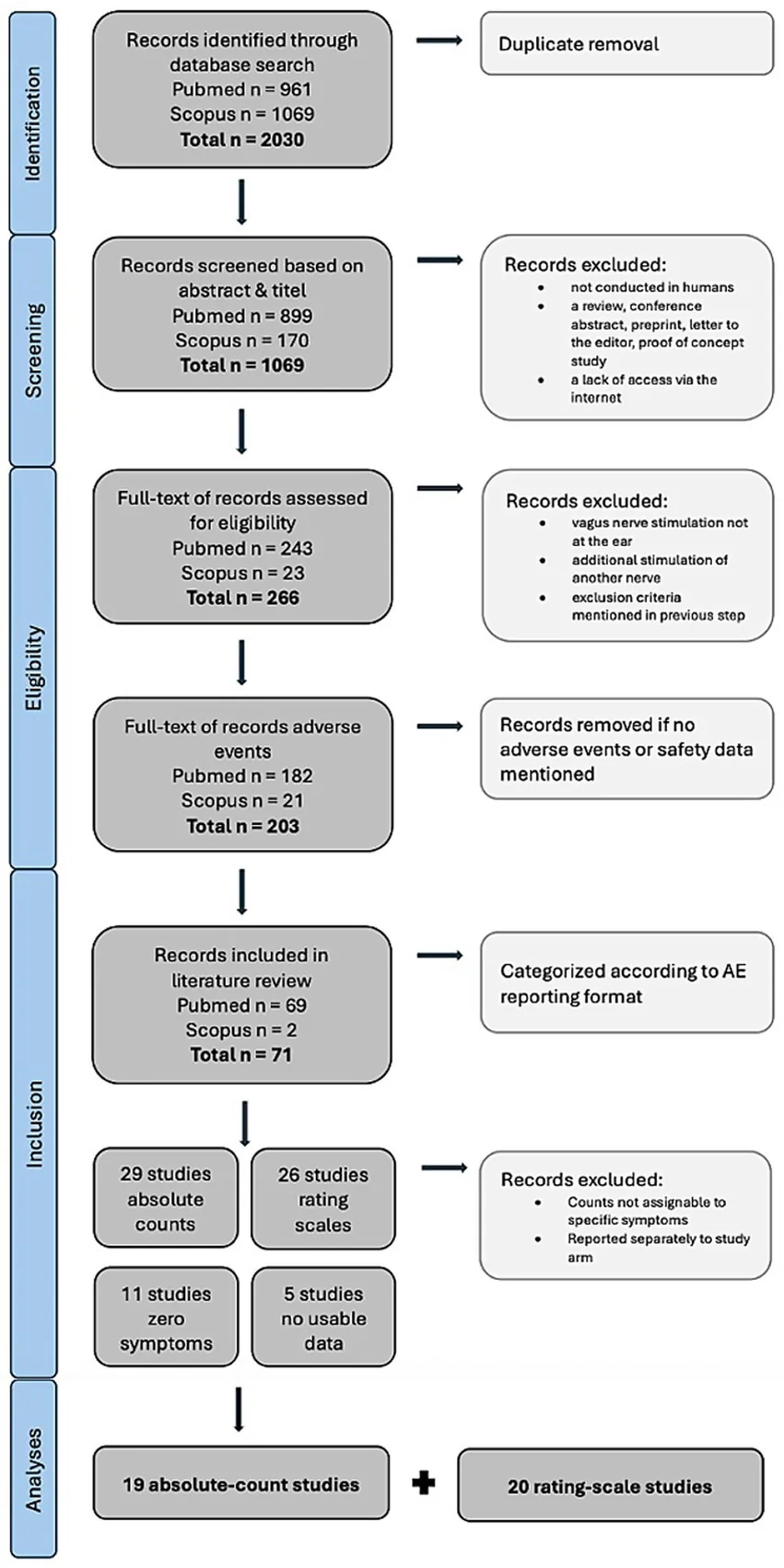
Flow chart of literature search adopted by PRISMA.

#### 2.2.3 Data extraction and statistical analysis

Data extraction was independently performed by two authors (KMB and TB), and disagreements were resolved through consultation with a third reviewer (LMS). The extracted variables included: (a) study metadata (authors, year, design, sample size and drop-out rate); (b) participant characteristics (age, sex ratio, and when applicable, diagnosis, disease severity, and duration); (c) taVNS parameters (target region, frequency, amplitude, pulse width, duration, study design, and sham condition); and (d) AEs, including detection methods, qualitative and quantitative AE data, and temporal relation to stimulation.

A quantitative meta-analysis was not conducted because of substantial heterogeneity in stimulation parameters, AE definitions, assessment methods, and reporting quality. Many studies lacked numerical AE data, used incompatible designs, or did not include sham controls, preventing the calculation of comparable effect estimates. Given these methodological constraints and the aim of generating an AE inventory, a descriptive synthesis is deemed the most appropriate approach.

### 2.3 Development of the questionnaire on adverse events in taVNS (taVNS-AEQ)

A semi-structured questionnaire was developed based on the review findings and in accordance with the CROSS and CONSORT-H guidelines (Sharma et al., 2021; Junqueira et al., 2023). The instrument was designed to integrate participant-reported and researcher-reported assessments, be easy to understand and administer (<10 min), and be applicable across diverse study populations and designs. An AE was included if the sign or symptom (1) appeared in more than three studies, (2) was reported more frequently during taVNS than during sham, (3) occurred more than five times, or (4) led to study discontinuation or was rated as severe. Items were selected (KMB), reviewed (LSM, TB) and partially adapted to symptom terminology followed the WHO and MedDRA standards (MedDRA: Medical Dictionary für Regulatory Activities, 2022; World Health Organization, 2022).

### 2.4 Cognitive pretesting

The preliminary taVNS-AEQ 1.0 underwent cognitive pre-testing using the *think-aloud* method (Wolcott and Lobczowski, 2021). One of the three case vignettes was administered to each of the 12 healthy participants right before the beginning of pretesting. A standardized introduction about the *think-aloud* technique was provided to ensure consistent testing conditions and a test question was asked to ensure that the participants understood the procedure. The participants were instructed to imagine that they hand received taVNS and experienced all five described symptoms written in the case vignette. The participants were allowed to use the vignettes and were asked to verbalize their thoughts aloud during questionnaire completion.

Retrospectively, participants were asked about comprehension difficulties and suggestions for improvement and rated the perceived difficulty of the instructions (1 = easy, 2 = moderate, 3 = severe).

The suggestions were incorporated by consensus of the research team, resulting in taVNS-AEQ version 2.0.

### 2.5 Pilot testing

The questionnaire 2.0 was then applied in a single-blind, crossover taVNS study with 29 healthy young adults to assess feasibility (Bömmer et al., 2024). The study was conducted in accordance with the Declaration of Helsinki and approved by the Ethics Committee of the University of Oldenburg for studies involving human participants (app. Number: 2020–026, February 17, 2022). All participants were informed about the study procedures, provided written informed consent prior to participation and indicated an absence of comorbidities and reported not using centrally acting medications.

Each participant received two real stimulations (left cymba conchae) and two sham stimulations (left earlobe) across two 45-min sessions, separated at least by 48 h. Stimulation differed only in the duty cycle (30 s on/30 s off vs. 3.4 s on/26.6 s off). Stimulation parameters were as follows: frequency 25 Hz, pulse width 250 μs, pulse form rectangular, duty cycle 0,625% on period, intensity was individually gradually increased before each stimulation block (starting at 0.1 mA) until the participants indicated the stimulation to be painful. We used a legacy ear electrode (NEMOS, Cerbomed, Erlangen, Germany). It was held in place with an earpiece, secured with leuctotape and electrode gel to reduction the impedance.

Each stimulation condition was treated as a separate observation, and both the participants’ questionnaires and researchers’ assessments were completed after each stimulation. Thus, repeated stimulation conditions within the same participant were analysed separately.

Less consistent questionnaire responses were managed according to predefined rules. Open-text responses were summarized descriptively (Supplementary Tables S6, S7). The responses were then discussed by the research group.

Following the pilot study, the German questionnaire 2.0 was revised to improve the assessment of AEs. Subsequently, the symptom items were further refined based on an updated targeted literature review, resulting in taVNS-AEQ version 3.0.

### 2.6 Statistical analysis

Given the small sample size and feasibility-oriented design and in line with the CONSORT-H, its update, and CROSS guidelines (Sharma et al., 2021; Junqueira et al., 2023; Ioannidis et al., 2004; Moher et al., 2001), AE frequencies were summarized and no formal statistical comparisons between active and sham conditions were performed. Visualizations followed the recommendations of Phillips et al. (2022) and Cornelius et al. (2020). The reported data included (i) the number of participants experiencing each symptom, (ii) the likelihood of a causal link to stimulation, (iii) symptom severity ratings, (iv) onset and duration of symptoms, and (v) study dropouts. These variables are essential for the characterization of AEs.

### 2.7 Translation of the taVNS-AEQ

The final German questionnaire 3.0 was translated into English, French, Spanish, and Italian by native speakers found through the University of Oldenburg. Each questionnaire was independently translated by one native speaker into the target language and independently back-translated by a second native speaker into German.

Discrepancies between the original and back-translated versions were discussed with the second translator and resolved through adjustments to the target-language version. Subsequently, the initial translator reviewed the adjustments and provided comments to ensure that the intended meaning was preserved.

## 3 Results

### 3.1 Item generation

#### 3.1.1 Targeted literature review

Note that an earlier and outdated version of the literature review was used as the basis for the development of taVNS-AEQ 1.0. After the pilot testing, the review was updated (years 2022–2025) and the item generation and the questionnaire were modified according to the updated results. The results mentioned in the following refer to the updated literature review.

The search identified 71 eligible studies, compromising a total of 3,581 participants, of whom 2,412 received taVNS. The cymba conchae was the most common stimulation site (*n* = 54, 76.1%). Sham stimulation most frequently involved earlobe stimulation (*n* = 31, 43.7%) or no electrical stimulation (*n* = 12, 16.9%). The NEMOS device (Cerbomed^®^) was used most often (*n* = 18; 25.4%), followed by TENS 7000 (*n* = 4; 5.6%), tVNS501 (Rishena^®^) (*n* = 4; 5.6%), and the tVNS L device (tVNS Technologies^®^) (*n* = 4; 5.6%). Stimulation parameters varied widely: amplitudes ranged from 0.1–12 mA, and stimulation frequencies from 5–100 Hz (most commonly 20–30 Hz). Stimulation was applied to the left ear in 52 studies, bilaterally in five, alternating sides in seven, and three did not report laterality. Most studies adjusted the intensity to individual perceptions or pain thresholds. The study characteristics are summarized in Supplementary Table S1.

#### 3.1.2 Detection methods for adverse events

In 61 of the 71 studies, AE detection methods were extractable. Assessment methods were not mutually exclusive, as individual studies use multiple methods to assess AEs. AEs were assessed using symptom-defined scales (*n* = 26), questionnaires (*n* = 11), patient reports (*n* = 18), open AE questions (*n* = 9), clinical observation/examination (*n* = 5), participant diaries (*n* = 6), vital sign monitoring (*n* = 15), and symptom-specific queries (*n* = 2).

Clinical studies typically use longer stimulation periods (weeks to months), whereas experimental studies in healthy participants apply single or short-term protocols. When stimulation was administered 1–5 times (*n* = 41), structured tools (questionnaires/scales/queries) were used most frequently (*n* = 26). In long-term studies, passive methods (patient reports, observation, and diaries) predominated. Studies using structured questionnaires provided the most detailed and comparable AE data.

#### 3.1.3 Symptom severity, drop-outs, and temporal patterns

Approximately 60% of the 71 studies reported symptom severity: 27 used Likert scales, and 14 used severity terms, although none referenced a standardized grading system (Foster et al., 2008). Most of the symptoms were rated as mild. Over one-third of the studies did not report drop-out information. Seven studies documented 1–2 drop-outs due to tinnitus (Rong et al., 2016), dizziness (Huang et al., 2014; Aihua et al., 2014), abdominal pain (Shi et al., 2024), painful pressure (Janner et al., 2018), pulmonary infection, or circulatory collapse (Laqua et al., 2014).

Eleven studies reported the temporal relationship between AEs and stimulation: in 7, symptoms resolved immediately after stimulation; in 4, they persisted for 5 min to 6 h.

#### 3.1.4 Frequency analysis of adverse events

For frequency analyses, the 71 included studies were first categorized according to their AE reporting format: 29 studies reported AEs as absolute counts, 26 used rating scales, 5 did not provide usable AE data and 11 reported no symptoms. Among the 55 studies that reported AE data (29 studies with absolute counts and 26 using rating scales), further eligibility criteria (assignable to specific symptoms, reported separately to study arm) were applied to determine whether the data could be included in the analyses. Following this assessment, 19 absolute-count studies and 20 rating-scale studies remained for qualitative and quantitative analyses (Figure 1).

In the 19 absolute-count studies, a total of 539 individual AE symptoms were reported. Of these, 388 were explicitly reported during or after taVNS. Skin-related AEs were the most prevalent (*n* = 170), including redness, itching, burning, tingling, wounds, pressure, pain, and unpleasant sensations. Additionally, symptoms such as nausea, insomnia, headaches and dizziness occurred most frequently. Supplementary Table 2 provides the severity data. Only 16 of 71 studies reported the number of affected participants and 15 of 71 studies reported an increased likelihood of stimulation-related symptoms. Studies applying taVNS for clinical treatment reported the majority of AEs (371 of 539 individual AE symptoms) possibly attributed to longer stimulation periods (1–3 months) (Liu et al., 2024; Zhou et al., 2023; Straube et al., 2015). In studies using rating scales, only scale scores were reported without defining thresholds for classifying symptoms as AEs or referencing standard scales. As the scales varied widely (0–3 to 0–100), mean values were not directly comparable. Only seven studies with rating scales reported significantly higher AE severity during taVNS versus sham (all earlobe), including burning (*n* = 4), tingling (*n* = 3), or painful (*n* = 1) sensation of the skin, ear irritation (*n* = 3), more distraction by stimulation (*n* = 2), arousal (*n* = 2), uncomfortable feeling (*n* = 1), nausea (*n* = 1), drowsiness (*n* = 1) and, in one study, positive sensations (butterflies in stomach, happy, pleasant feeling).

To provide an overview of symptom assessment across the literature irrespective of the reporting format used, Table 1 summarizes the number of studies in which each symptom was evaluated. The most frequently assessed AEs were neck pain, headaches, nausea and malaise.

**Table 1:** Frequency of symptom reporting across studies included in targeted literature review (39 studies).

Adverse events	Number of studies examining AEs
Neck pain	24
Headaches	23
Nausea	22
Malaise (unspecific)	21
Facial and/or neck muscle cramps	18
Dizziness	16
Burning of the skin	15
Tingling sensation	14
Stinging sensation	13
Changes in concentration	12
Unpleasant skin sensation	11
Cardiovascular symptoms (e.g., palpitation, weakness, collapse)	9
Fatigue	8
Fluctuation of feelings	8
Ear discomfort (e.g., feeling of fullness, pressure)	7
Itching	7
Skin redness	5
Tinnitus/ear noises	5
Arousal	4
Skin pain	4
Insomnia	3
Pain	3
Stomach pain	3
Constipation	3
Pleasant feelings	2
Numbness of a limb	2
Vomiting	2
Skin pressure sensation	1
Feeling of heat	1
Chest pain	1
Diarrhea	1
Dry mouth	1
Dysphagia	1
Epistaxis	1
Fluid in the air	1
Grinding teeth	1
Happy feelings	1
Hidrosis	1
Hip pain	1
Hoarseness	1
Light-headedness	1
Rush of blood to temples	1
Sexual dysfunction	1
Slight heedfulness	1

#### 3.1.5 Development of the taVNS-AEQ

AEs identified in the targeted literature review were systematically integrated into the taVNS-AEQ 1.0 using a combined open- and closed-ended structure. The introductory open question (“Have you noticed stimulation symptoms during this study cycle?”) is followed by a symptom checklist, enabling standardized quantification and direct comparison across the sessions. To ensure cross-study comparability, most items employed categorical response formats. Figure 2 illustrates the 25 symptoms included in the checklist in taVNS-AEQ version 3.0. Insomnia and constipation were positioned at the end of the list, as these AEs are relevant primarily for long-term stimulation protocols. Although one study reported increased positive sensations (e.g., “pleasant feeling,” “happy,” “butterflies in the stomach”) during taVNS compared with sham, these phenomena do not meet the formal definition of AEs and were, therefore, excluded.

**Figure 2:**
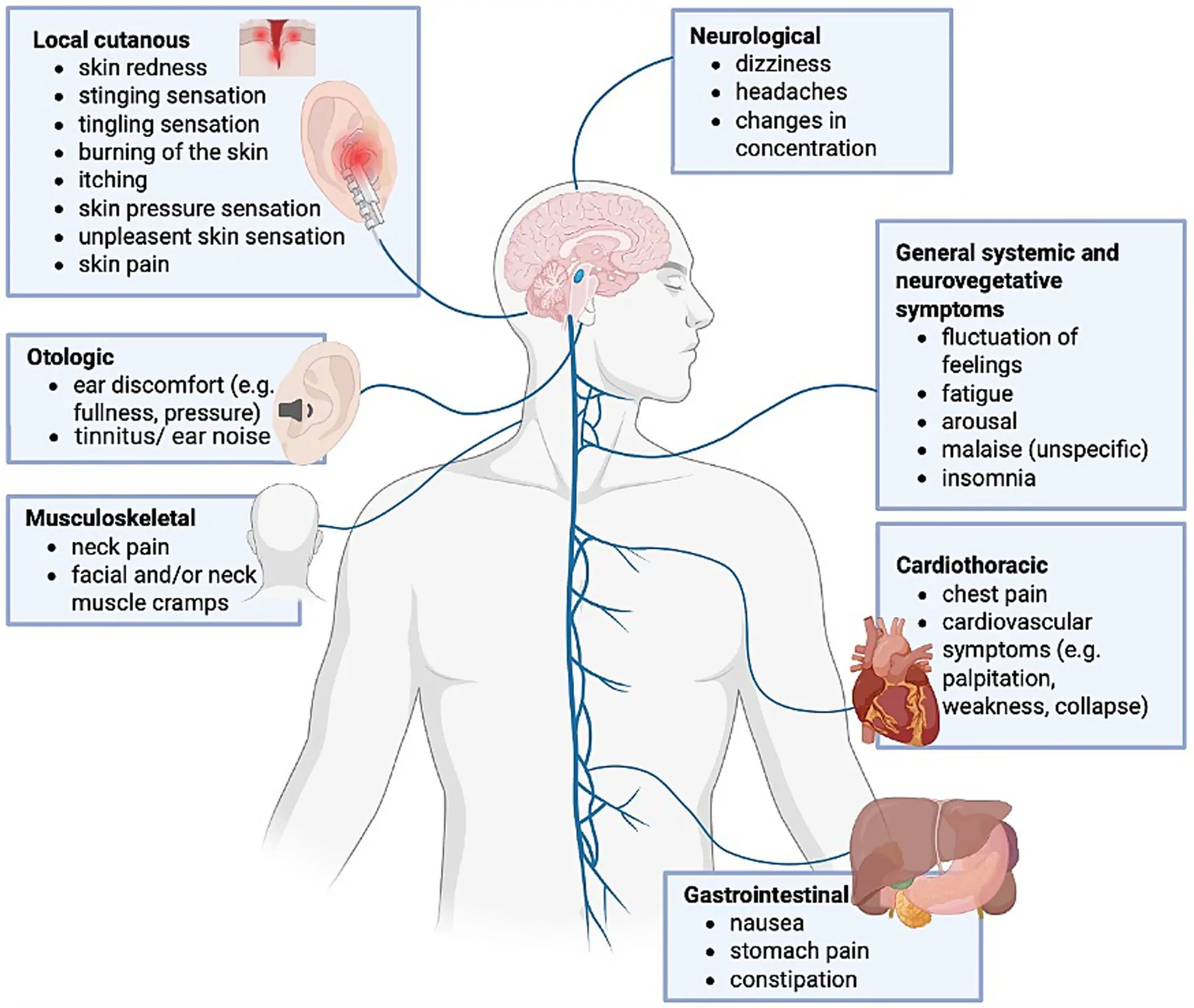
Signs and symptoms commonly reported during or after taVNS included for assessment in the taVNS-AEQ. The elements of this figure were created using BioRender.com.

To characterize each reported symptom, the questionnaire captured severity (mild, moderate or severe), onset (beginning, middle, end or after the study cycle), persistence after stimulation (present or disappeared), duration (≤10 min, 11–25 min, 26–49 min, 50 min (beginning until end), or only during electrical stimulation), potential reason (study setting or participation, stimulation itself, pre-existing medical condition, symptom known or randomly without apparent reason), and strength of impairment during the study (impairing yes or no). An open field allowed the reporting of additional symptoms not listed in the checklist. Participants also indicated their willingness to participate in future taVNS studies and whether they believed they received real or sham stimulation.

The second section captures the perspective of the study personnel, including stimulation intensity (and adjustments), observations, dropouts and pre-existing medical conditions. Both the participant and coordinator sections were completed after each session, and explanations were provided only upon request to maintain procedural consistency.

### 3.2 Cognitive pretesting

In the pre-test using the *think-aloud* method, 9 of the 12 participants rated the questionnaire as easy and 3 as moderately difficult. Completion of the vignette-based version (five symptoms) required a mean of 14:02 min. No structural modifications were proposed. The questionnaire 1.0 was revised on the basis of the findings during cognitive testing (Table 2).

**Table 2:** Revisions of the taVNS-AEQ 1.0 after cognitive pretesting.

Aspect	Finding during cognitive testing	Resulting revision
Questionnaire instructions	Initial difficulties understanding how to complete symptom table	Instructions revised and additional arrows added for guidance
Generalized body pain	Term caused comprehension difficulties	Term removed from the symptom list, multiple pain locations can still be reported under “other symptoms”
Malaise	Meaning was considered unclear	Specified as unspecific malaise
Ear discomfort	Considered insufficiently specific	Clarified as ear fullness or pressure
Unpleasant skin sensation	Intended meaning not sufficiently clear	Renamed other unpleasant skin sensations and placed last in symptom list for electrode site
Tinnitus	Participants needed time to understand term	Ear ringing added as a descriptor
Question active /sham	Two participants did not understand the meaning of for control purposes	Wording revies to explicitly describe the absence of stimulation and the simulation of stimulation to analyse placebo effects
Symptom onset/duration	Free-text reporting of symptom onset/duration was considered less suitable	Replaced by a categorical response scale
Study discontinuation	To facilitate accurate documentation if discontinuation occurs	Question transferred to study coordinator questionnaire

All 12 participants understood the questionnaire correctly. Minor differences in symptom assignment and interpretation of severity and relatedness were observed across the case vignettes, but no further revisions were considered necessary by the research group (KW, LMS, TB, KMB). No additional difficulties or explicit suggestions for improvement were reported by participants during retrospective interviews.

### 3.3 Pilot testing

In the taVNS study, 29 participants completed 111 questionnaires. Two participants did not complete the study. One participant was lost to follow-up with an unknown reason for discontinuation. One additional participant withdrew from the study, stating the burden and the duration of the study procedure as explanation. No AE was reported as the reason for withdrawal. In one of the 27 participants, the stimulation condition of the second appointment was incorrectly designated, leading to a repetition of one condition, therefore only 3 questionnaires from this participant were included. Of the 111 questionnaires, 26 were completed after 3.4 s of active stimulation, 29 after 30s of active stimulation, 28 after 3.4 s of placebo stimulation and 28 after 30s of placebo stimulation. All participants who answered “yes” to the introductory open question subsequently reported at least one symptom on the checklist (Table 3). Notably, half of those who initially answered “no” later indicated one or more symptoms. Overall, app. 80% of the questionnaires contained at least one symptom.

**Table 3:** Cross-table to compare the results of the open-ended introductory question and the specific symptom-related questions.

Response to specific symptom-related questions	Open-ended introductory question		
	Yes	No	Total
Yes	73 (65.76%)	19 (17.12%)	92 (82.88%)
No	0 (0%)	19 (17.12%)	19 (17.12%)
Total	73 (65.76%)	38 (34.24%)	111 (100%)

Across all the sessions of the 29 participants, 235 symptoms were documented (Supplementary Tables S3, S6). Descriptively, the frequency of symptoms was higher during real stimulation (64/235) than during sham stimulation (51/235) in the short-pulse condition (3.4 s), whereas the reverse pattern (59/235 stimulation, 61/235 sham) was observed in the long-pulse condition (30 s). Up to six symptoms per study session (sham/real/3.4 s/30s) were reported (Supplementary Table S4). The most frequent symptoms of all 111 questionnaires were unpleasant tingling (60 (29 stimulation/31 sham) of 235), fatigue (57 (31 stimulation/26 sham) of 235), decline in attention (39 (31 stimulation/18 sham) of 235), headache (16 (10 stimulation/6 sham) of 235), itching (10 (7 stimulation/3 sham) of 235), and neck pain (10 (6 stimulation/4 sham) of 235) (Supplementary Table S5). Five symptoms did not occur (skin wounds, numbness, pulse-change sensation, hoarseness, and nausea). Muscle cramps in the face or neck occurred exclusively during the real taVNS. A difference of a frequency >3 of symptoms between real (30s + 3.4 s) and sham (30s + 3.4 s) were observed only for fatigue, headache, and skin itching (Figure 3). The lowest risk ratio (descriptive) during stimulation was observed for tinnitus and ear noises.

**Figure 3:**
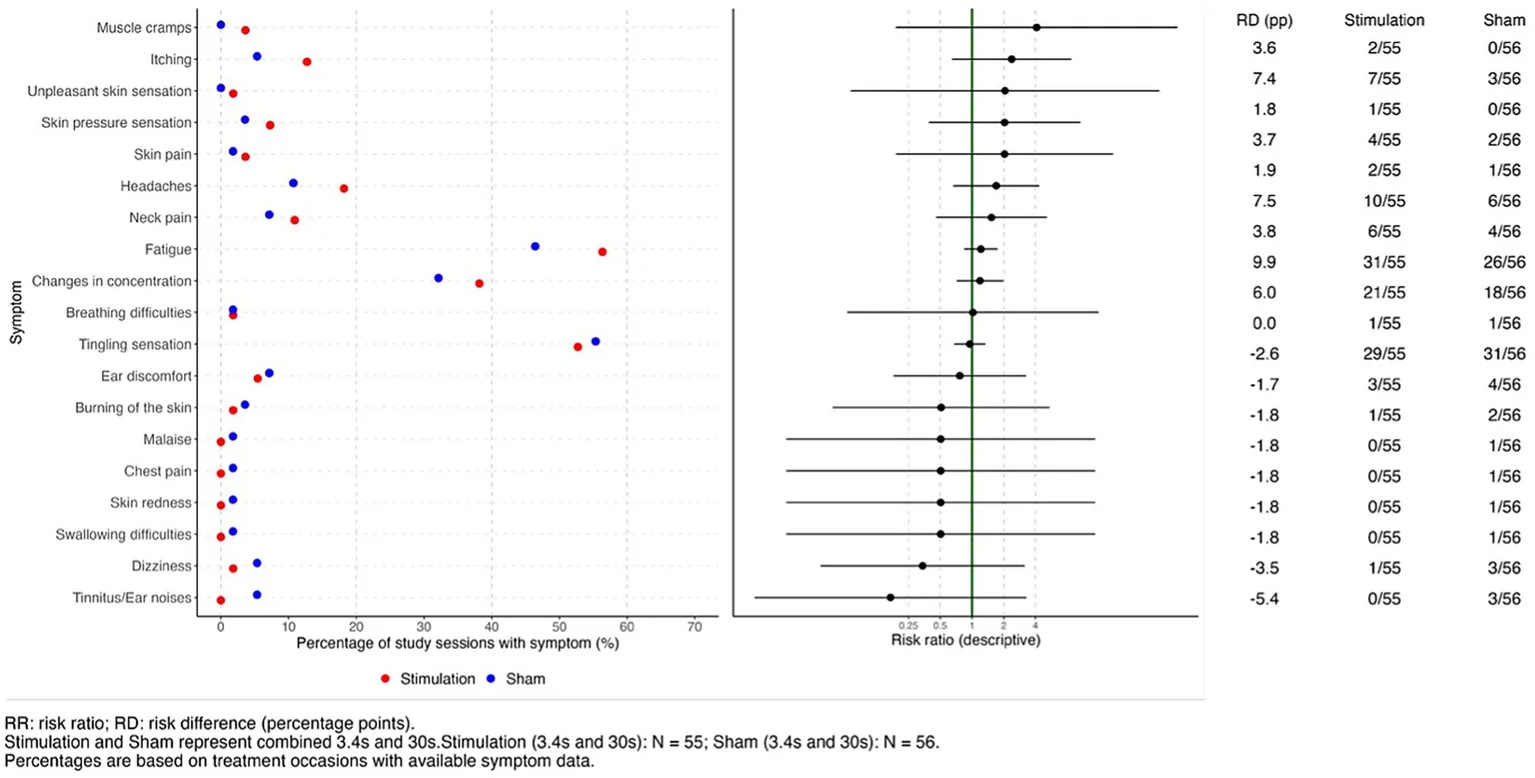
Symptom occurrence during stimulation and sham stimulation sessions. Percentages represent the proportion of study sessions in which each symptom was reported. Stimulation and sham sessions are combined (30s and 3.4 s), resulting in 55 stimulation sessions and 56 sham sessions. The estimates and CI should be interpreted only descriptively, as no within-participant clustering was performed. Left: Symptom frequencies as percentages with corresponding numerators and denominators in the right table. Middle: Risk ratio (RR) for stimulation versus sham with confidence intervals. Right: Risk difference (RD) in percentage points (PP).

Of the 227 symptoms in the 111 questionnaires with severity ratings (excluding the participant-reported 8 symptoms in the free-text field), seven reached the “severe” category (2 stimulation/5 sham), and most were rated mild (Figure 4). The symptoms most frequently reported by participants as impairing were fatigue (59.65%, 34 impairing of 57 fatigue symptoms: 34/57), changes in concentration (46.15%, 18/39), skin pain at the electrode site (33.33%, 1/3), tingling (31.67%, 19/60), and headaches (31.25%, 5/16) (Supplementary Table S5). Symptom onset ranged from 0.2 to 40 min, with comparable variability in duration (Supplementary Table S6).

**Figure 4:**
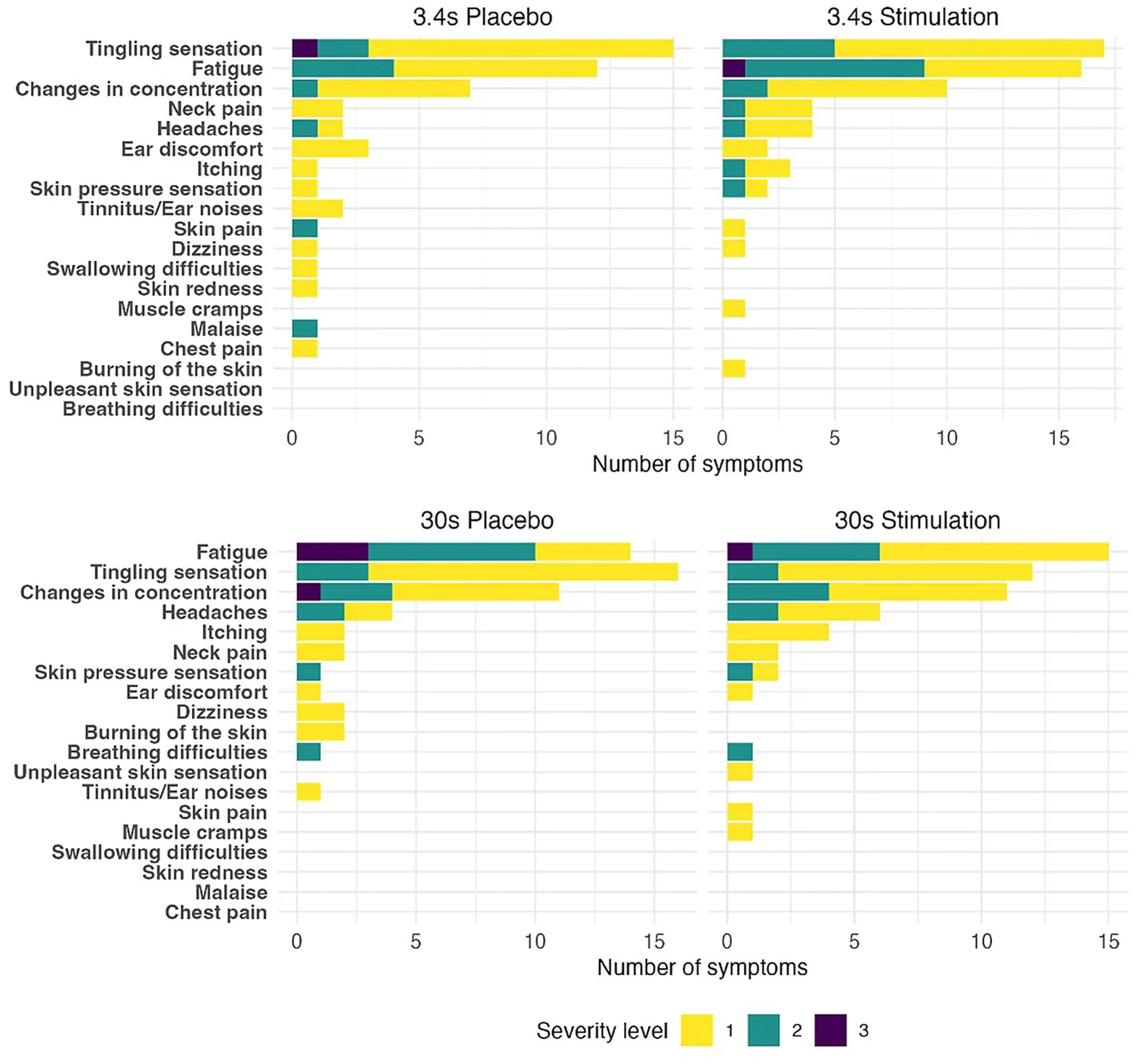
Horizontally stacked bar chart illustrating the number of severity levels per symptom (1 = mild, 2 = moderate, 3 = severe). For the number of symptoms reported in all 111 questionnaires, the reported intensity is indicated by colour, separately for the four conditions (3.4 s placebo, 30 s placebo, 30 s stim, 3.4 s stim).

Four participants reported eight additional symptoms in the open fields. The study coordinators documented 14 additional symptoms in seven participants, including decline in attention (*n* = 2), fatigue (*n* = 2), twitching (*n* = 1), itching (*n* = 1), increased blinking (*n* = 1), pressure marks (*n* = 1), altered vision (*n* = 1), and transient ptosis (*n* = 1).

Of the 111 completed questionnaires, 69/111 (62.16%) were classified by participants as active stimulation and 42/111 (37.84%) as sham stimulation. Of these, 36 of 69 (52.17%) active-stimulation and 23 of 42 (54.76%) sham-stimulation classifications were correct, resulting in 59/111 (53.15%) correct classifications overall.

No AE-related dropouts or clinically relevant health changes occurred. The stimulation intensity was reduced in 6 of the 111 sessions (5.41%) (Supplementary Table S7). Completion time ranged from 5 to 10 min depending on the symptom load.

The pilot study resulted in the following changes to the taVNS-AEQ version 2.0 after consensus of the research group (Table 4).

**Table 4:** Revisions of the taVNS-AEQ 2.0 after pilot study.

Aspect	Resulting revision
Questionnaire structure	Questions about symptoms were placed at the beginning to assess the number of symptoms reported without questions about predefined symptoms to identify potential discrepancies with the number reported following the subsequent assessment of specific symptoms
Response scale position	In the table, the response scales were provided only at the top of the column; in the revised version, the corresponding response options are provided in each item row for every symptom, so every part has to select a response for each symptom, thereby the risk of incomplete or ambiguous responses is reduced
Follow-up questions for reported symptoms	Items in the symptom table were revised: in the previous version, participants were asked about symptom severity, timing, free-text duration and whether the symptom was previously known. In the revised version, these items were replaced by questions assessing symptom severity, onset, whether the symptom persisted after the end of study, total duration, presumed cause and whether the symptom was perceived strongly impairing in categorial response form. Accordingly, the additional question asking which symptoms were perceived as impairing was removed in Version 3.0.
Confidence in stimulation identification	Following the question on whether participants believed they had received active or sham stimulation, their confidence in this assessment was evaluated using five categories
Study coordinator-administered questionnaire	Question 7 was revised to record participants’ known pre-existing medical conditions and current medications
Symptom organization	The order of symptoms was revised and symptoms were grouped according to organ systems.

After completing the pilot study, the literature review was updated, which led to further changes to the item generation independently of the pilot study findings. As described in the methods and results section, symptom items were reassessed against the predefined inclusion and exclusion criteria. Consequently, some previously included symptoms no longer met the inclusion criteria and were removed (difficulty breathing, voice disturbance/hoarseness, swallowing difficulties, and skin wounds), while newly identified symptoms meeting the criteria were added (tingling sensation, fluctuation of feelings, arousal, constipation, and insomnia). The number of symptom items increased from 24 to 25 items.

The version 2.0 used in the pilot study (Supplementary material 10), the final multilingual questionnaires version 3.0 (English, German, French, Italian, and Spanish) and instructions for the application and analysis of the questionnaire (Supplementary material 9) are provided in the Supplementary material.

## 4 Discussion

The present study followed a multistage methodological approach with literature-based item generation, cognitive pretesting, pilot testing, and translation to develop and preliminarily evaluate the taVNS Adverse Events Questionnaire (taVNS-AEQ). In general, these stages were designed to develop the questionnaire and assess its comprehensibility, usability, and feasibility for reporting AEs in the context of taVNS studies.

For item generation, a targeted literature search was conducted. Despite the initially identified 203 records in this review, only app. 19.21% (19 absolute-count studies and 20 rating-scale studies) provided qualitative and quantitative data on AEs. A potential reason for the limited systematic reporting of AEs is the perception of taVNS as a safe and low-risk intervention, which leads many studies to prioritize efficacy outcomes over detailed safety assessments. In this context, the development of a user-friendly and semi-structured questionnaire is crucial for enhancing the consistency, comparability, and quality of AE reporting in future taVNS studies.

### 4.1 Adverse events of taVNS

Our review showed that for item generation, in studies reporting absolute AE counts, skin burning and tingling sensations, nausea, insomnia, headache, and dizziness were the most commonly reported AEs. In studies using rating scales, neck pain, headache, nausea, and malaise were the most frequently symptoms. This finding aligns with two prior meta-analyses that compiled data on tVNS via the cervical and auricular routes (Kim et al., 2022; Redgrave et al., 2018), which contrasts with invasive vagus nerve stimulation.

In our pilot study employing the taVNS-AEQ 2.0, symptoms such as tingling sensation, fatigue, concentration changes, headache, itching, and neck/ear discomfort appeared more frequently in real taVNS than in sham stimulation of the earlobe, these observations were descriptive. Given that our list of the most prevalent AEs reported differs from those reported in our and other reviews, prospective, standardized, and sham-controlled studies are needed to delineate the spectrum and frequency of taVNS effects. Furthermore, the stimulation parameters also influence the frequency and type of AEs (Giraudier et al., 2020).

The literature and interest in trigeminal stimulation for modulating cognitive function are expanding (Mercante et al., 2018; Westwood et al., 2023). Auricular stimulation applied outside the vagal innervation territory can elicit trigeminal and cervical stimulation effects. Therefore, it is crucial to incorporate the place and intensity parameters of sham stimulation alongside real taVNS to differentiate between various stimulation effects originating from different stimulation sites.

No participant experienced any severe cardiac events in this study. In addition, Redgrave et al. (2018) showed that taVNS is not associated with higher number of cardiac AEs. This is a feared AE, especially during invasive VNS (Asconapé et al., 1999; Ali et al., 2004) or right-sided stimulation (Panebianco et al., 2016).

### 4.2 Rationale für AEQ development

The systematic collection of taVNS symptoms would be most effectively facilitated by consistently implementing an AE questionnaire. When this adheres to a structured protocol, it can further enhance the benefits of standardization. Given the expanding range of stimulation parameters, stimulation outcomes, sham stimulation variants, and diverse disorders for which taVNS is employed, it is crucial to systematically evaluate AEs for the safety of both patients and practitioners. The necessity for a new questionnaire stems from the absence of a widely accepted taVNS-AEQ, as existing tools have not yet achieved sufficient standardization or widespread adoption in research and clinical practice. In light of this gap, we assessed the feasibility and usability of the newly developed instrument through several methodological stages. Although the literature review included AE information from both the healthy volunteers and clinical populations, the next two steps, cognitive pretesting and pilot testing, were initially conducted in healthy participants to assess the comprehensibility and feasibility of the item pool under relatively controlled conditions. Specifically, before its application, the questionnaire underwent cognitive pretesting using a think-aloud approach, followed by iterative refinements based on participant feedback to enhance item clarity and comprehensibility. The initial pilot application of the questionnaire in a real-sham crossover taVNS study involving 29 healthy participants further informed additional adaptations, thereby enhancing its overall usability.

In previous studies the characterization of AEs associated with taVNS were already analysed and the AEs were indicated as well-tolerated. (Kim et al., 2022; Redgrave et al., 2018; Giraudier et al., 2025; Gerges et al., 2024). To date, only a few studies developed and published questionnaires for the structured assessment of AEs (Kim et al., 2022; Giraudier et al., 2025; Gerges et al., 2024). Kim et al. (2022) developed a questionnaire based on the most frequently reported AEs in their meta-analysis, including questions about the severity of symptoms, relatedness to taVNS and an open question for additional symptoms.

Gerges et al. (2024) proposed an AE questionnaire specifically informed by AEs reported in clinical populations. Giraudier et al. (2025) adapted an existing questionnaire to assess 10 AEs. Thus, this is not the first study developing an AE questionnaire for taVNS studies. However, existing questionnaires are not considered as established instruments in the literature, and consequently, no tool to facilitate comparability or reduce heterogeneity of reporting AEs exists.

Comparing the taVNS-AEQ 3.0 with existing questionnaires (Kim et al., 2022; Giraudier et al., 2025; Gerges et al., 2024), it provides a more comprehensive and structured assessment of AEs associated with taVNS (Supplementary Table S8). It was developed during multi-methodological stages, whereas the existing questionnaires were established after one analysis without reported development process with revisions or applications. In addition, the taVNS-AEQ 3.0 covers a broader range of symptoms – with an open question about symptoms plus 25 predefined symptoms – versus 10 predefined symptoms plus “others” in the three existing questionnaires. Notably, in the taVNS-AEQ 3.0 the participants are first asked about if any symptoms occurred in an open-question before asking specifically about predefined symptoms to distinguish spontaneous and prompted answers. Furthermore, this questionnaire includes questions only for the study coordinator to see both perspectives and obtain further information about the symptoms or observations as the importance of specific events differs between study coordinators and participants (Zhu et al., 2011; Basch et al., 2009; Wetzels et al., 2008). All existing questionnaires ask about symptom severity with rating scales. However, only in this questionnaire are questions about symptom onset/timing, duration, symptom persistence after the study, assessment of symptom impairment and the potential relatedness to stimulation in a categorical response format. When possible, the number of free-text answers was reduced in order to avoid ambiguous answers and facilitate structured assessment and date collection. This is the only questionnaire available in more than one language.

Overall, the features of the taVNS-AEQ 3.0 offer additional value as it covers a broad symptom range with semi-structured assessment of symptom characteristics and study coordinator-observed findings. This enables a consistent and comparable AE assessment in taVNS studies.

### 4.3 Methodological challenges in AE assessment during taVNS

When applying a semi-structured questionnaire, it should be acknowledged that highly detailed taVNS-AEQs may increase the risk of overreporting minor or non-specific symptoms. However, this potential limitation is generally outweighed by the risk associated with the absence of a structured AEQ, namely substantial underreporting of AEs. Without systematic assessment, relevant symptoms may remain undetected, particularly in clinical populations where both therapeutic and potential AEs are insufficiently characterized. Therefore, especially in studies involving patient groups rather than healthy participants, detailed and standardized AE monitoring is essential to ensure safety reporting.

Furthermore, earlobe stimulation was used in the sham condition in many taVNS studies. However, it cannot be assumed to be completely physiologically and perceptually inert. It should be mentioned that in our study, a considerable proportion of AEs also occurred during sham stimulation, reflecting nonspecific effects at this stimulation site. Stimulation of the earlobe can potentially produce nonspecific cutaneous or other nonspecific symptoms. However, the earlobe is assumed to be a suitable sham site because the stimulation effects at the earlobe are considered to be comparable to those at the cymba conchae (Sharon et al., 2021). Perhaps, because of the nonspecific stimulation effects during sham stimulation in our study, the proportion of AEs occurred during sham and the participants were generally not able to distinguish between active and sham stimulation. Importantly, the taVNS-AEQ 3.0 was developed to facilitate data collection of AEs during taVNS. When AEs are assessed in many more taVNS studies with different simulation parameters, sham stimulation sites and population groups, pattern of occurrence of AEs can help to distinguish between AEs related to taVNS alone and AEs attributable to the study characteristics.

### 4.4 Recommendations for taVNS-AEQ

The recommendations formulated by Redgrave et al. (2018) were utilized as one source for developing the taVNS-AEQ, with additional recommendations that incorporated: (i) routinely report safety and tolerability data in any study involving taVNS in human participants, (ii) employ a standard definition of SAE and a standardized method for measuring AEs, such as open-ended questions with a severity indicator, (iii) fully disclose all taVNS stimulation parameters, including frequency, pulse width, amplitude, and either the cumulative exposure time or the variables necessary to calculate it, (iv) report the proportion of participants experiencing “any” AE as well as the proportion experiencing each individual AE (Redgrave et al., 2018), including drop-outs and reasons for discontinuation, (v) utilize both a participant-reported and a study coordinator-administered questionnaire, (vi) provide detailed reports of AEs in the sham condition, including all parameters (stimulus side, frequency, pulse width, stimulation duration) reported also in the real stimulation condition, (vii) report comorbidities, additional medication, and the clinical study population in detail to collect safety data in vulnerable patient groups. Particularly in clinical applications involving patient populations, the use of such a newly developed semi-structured questionnaire is crucial, as it facilitates the systematic, complete, and comparable capture of symptoms, thereby reducing the risk of overlooked or underreported AEs.

The item pool of the taVNS-AEQ is considered a framework that can be further adapted and evaluated for specific target populations. The open question in the taVNS-AEQ about “other” experienced symptoms with free-text answers helps to detect population-specific symptoms. For example, for the application in patients with globus pharyngeus, other symptoms that are particularly relevant to this clinical population have to be considered, in this case symptom domains related to globus sensation, such as throat pressure or tightness, swallowing-related sensations, and other pharyngeal or laryngeal symptoms (Meng et al., 2026; Hang and Meng, 2025). Such population-specific adaptation should complement, rather than replace, the initial item-generation used in this study.

Additionally, to evaluate potential long-term adverse effects, the taVNS-AEQ could be administered again 24 h later.

### 4.5 Limitations

The literature search was restricted to the databases PubMed and Scopus and it was not updated after May 17, 2025. Therefore, it may not have captured all relevant publications, and the data are not captured in the item pool.

Notably, the method of AE detection has a considerable influence on the number of symptoms reported, particularly actively asked symptoms are more likely to be identified than in studies that capture symptoms only through spontaneous reporting. Furthermore, the heterogeneity in the reporting of stimulation parameters and AEs is an inherent limitation of every taVNS AE analysis. Consequently, it was not feasible to conduct a correlation analysis between the stimulation parameters and AEs or to perform further comparisons between the sham and stimulation groups. The analysis focused on descriptive statistics. Among the 71 studies that mentioned AEs, crucial information such as stimulation parameters, precise method of AE detection, number of individuals experiencing these symptoms, and additional details were frequently unavailable. Given the limited information available for some studies, the method of AE detection, stimulation parameters, stimulation duration, frequency of stimulation and overall duration of the study were not considered in the development process for item generation.

Since AEs were not systematically assessed or reported in many taVNS studies, as described in the literature review, it was necessary to consider healthy and clinical populations to obtain a broad overview of AEs in various study contexts for item generation. Cognitive pretesting and pilot testing were conducted in healthy participants, whereas the literature review included both healthy and clinical populations with different ages, underlying conditions, medication use, baseline symptoms and stimulation exposure. Further evaluation of the taVNS-AEQ before application in distinct population groups, study sizes and stimulation settings may therefore be required, as these factors can influence the occurrence and reporting of AEs.

Moreover, psychometric evaluation, including assessment of factor structure and reliability was not evaluated and will be required in future studies. Nonetheless, the first versions (1.0 and 2.0) of the taVNS-AEQ underwent a comprehensive multi-stage testing and refinement process, which included cognitive pretesting and iterative revisions across various study phases, thereby enhancing its overall feasibility and usability. However, the final version 3.0 of the taVNS-AEQ has not yet undergone feasibility and psychometric evaluation.

## 5 Conclusion

This study describes the development of a semi-structured instrument for the systematic reporting of AEs associated with taVNS. There is growing interest in the effects and therapeutic applications of taVNS. AEs, although crucial for translating research findings into therapeutic applications, they have received less attention. This is particularly significant when the stimulation parameters, outcome variables, and medical conditions are more heterogeneous.

Consequently, the taVNS-AEQ was developed through a multistage methodological process comprising a targeted literature review for item generation, cognitive pretesting, pilot testing, and translation into several languages. In these steps, the feasibility and usability of the newly developed semi-structured questionnaire was demonstrated as a tool to detect AEs and its characteristics.

Further psychometric evaluation and validation across independent study centres, clinical populations, and language versions will be important to establish broader applicability of the taVNS-AEQ. Systematic AE reporting may also facilitate the identification of patterns in AEs and explore their correlations with stimulation settings, which could contribute to improved safety monitoring in taVNS research and clinical practice.

## Data Availability

The original contributions presented in the study are included in the article/Supplementary material, further inquiries can be directed to the corresponding author.
