# Supplementary material for: Development and feasibility testing of the taVNS adverse events questionnaire: a multistage methodological study
Source: Front Neurosci. 2026 Oct 28;20:1974496. doi: 10.3389/fnins.2026.1974496 (PMC13752190; doi:10.3389/fnins.2026.1974496)
Supplement: Supplementary file 1 [file Supplementary_file_1.pdf]

Development and feasibility testing of the taVNS Adverse Events Questionnaire (taVNS-AEQ): a multistage methodological study

Katharina Meier-Bartelt[1], Luisa M. Schmidt[1], Till Bömmer[1,2] und Karsten Witt[1,2,3]

1 Department of Neurology, Carl von Ossietzky University, 26121 Oldenburg, Germany
2 University Clinic for Neurology at the Evangelical Hospital, 26121 Oldenburg, Germany
3 Research Center Neurosensory Science, Carl von Ossietzky University, 26129 Oldenburg, Germany

Supplement

Table of contents

Supplementary Table S1 .... **3**

Table 1: Characteristics of the studies used in the targeted literature review *3*

Supplementary Table S2 .... **11**

Table 2: Overview of symptom assessment across studies and reported severity levels *11*

Supplementary Table S3 .... **25**

Table 3: Frequency of symptoms documented during the taVNS sessions (111 questionnaires) *25*

Supplementary Table S4 .... **27**

Table 4: Distribution of reported symptoms per participant across stimulation sessions during taVNS (111 questionnaires) *27*

Supplementary Table S5 .... **28**

Table 5: Frequency of reported symptoms during taVNS sessions (111 questionnaires) and participant-rated impairment *28*

Supplementary Table S6 .... **28**

Table 6: Participant-reported symptoms with onset und duration during the taVNS sessions (111 questionnaires) *28*

Supplementary Table S7 .... **29**

Table 7: Documentation of reasons for stimulation intensity reduction during taVNS sessions (111 questionnaires) *29*

Supplementary Table S8 .... **29**

Table 8: Comparison of the newly developed semi-structured taVNS-AEQ 3.0 with existing instruments *29*

Supplementary Material 9 .... **31**

9.1: TaVNS-AEQ – English version 3.0 .... *32*

9.2: TaVNS-AEQ – German version 3.0 .... *42*

9.3 TaVNS-AEQ – Italian version 3.0 .... *53*

9.4 TaVNS-AEQ – Spanish version 3.0 .... *63*

9.5 TaVNS-AEQ – Frensh version 3.0 .... *73*

9.6 Instructions for application and analysis of questionnaire *83*

Supplementary Material 10 .... **84**

TaVNS-AEQ – Old German version 2.0 used in study *84*

Supplementary Table S1

Table 1: Characteristics of the studies used in the targeted literature review

Digital Object Identifier	Author, Year, Reference	Outcome parameter	Study design	Stim. region	Stim. side	Stim. device	Fre-quency (Hz)	Intensity (mA)	Stim. block	Study duration	Number of subjects (taVNS/ Sham)	Sham group
10.1093/eurheartj/ehaf037	Ackland, G. L., et al., 2025	Exercise capacity	single-centre, randomized, double-blind, sham-controlled, crossover trial	auricular tragi regions	left and right	TENS	25	individual threshold (between 4 and 12mA)	continuous	for 7 days daily	28 (28/28)	no current
10.1016/j.yebeh.2014.08.005	Aihua, L., et al., 2014	Epilepsy	randomized sham-controlled, intervention study	cymba conchae	left and right	TENS-200	20	individual	continuous	3x/day for 12 months	60 (30/30), analyzed: (26/21)	earlobe
10.1016/j.brs.2016.07.004	Beste, C., et al., 2016	Gamma-aminobutyric acid (GABA)	single-blinded, sham-controlled intervention study	cymba conchae	left	CM02/NEMOS	25	0,5	30s on - 30s off	one-time	51 (25/26)	external ear
10.1016/j.nlm.2016.05.007	Burger, A. M., et al., 2016	Emotion	randomized controlled study	cymba conchae	left	NEMOS	25	0,5	30s on - 30s off	one-time	31 (18/13)	earlobe
10.1038/s41598-018-29561-w	Burger A. M., et al., 2018	Fear extinction learning	randomized single-blinded controlled trial	cymba conchae	left	NEMOS	25	0,5	30s on - 30s off	one-time	85 (42/43)	earlobe
10.1016/j.biopsycho.2019.01.014	Burger, A. M., et al., 2019	Emotion	experimental, randomized study	cymba conchae	left	NEMOS	25	0,5	30s on - 30s off	one-time	97 (48/49) (only 91 completed questionnaire)	earlobe
10.1016/j.brs.2012.04.006	Busch, V., et al., 2013	Pain	double-blinded, randomized, experimental, controlled, cross-over design	tragus	left and right	STV02 Cerbomed	25	0,25 - 10	.	two-times	48 (48/48)	no current
10.1007/s00221-024-06876-x.	Camargo, L., et al., 2024	Cognition, Emotion	randomized, double-blind, sham-controlled	cymba conchae	left and right	Healaon	30	0,5 - 2,5	continuous	one-time	44 (22/22)	no current

Digital Object Identifier	Author, Year, Reference	Outcome parameter	Study design	Stim. region	Stim. side	Stim. device	Fre-quency (Hz)	Intensity (mA)	Stim. block	Study duration	Number of subjects (taVNS/ Sham)	Sham group
10.1155/2017/7876507	Capone, F., et al, 2017	Stroke,	proof-of-principle, double-blinded, semi-randomized, sham-controlled study	Tragus (inner side)	left	Twister	20	Individual	30s on – every 5min	1x60min for 10 days	12 (7/5)	earlobe
10.1016/j.clinph.2021.05.014	Capone, F., et al., 2021	Pupil size	randomized, single-blinded, cross-over within-subject design	tragus (inner side)	left	Digitimer DS7	20	0.5, 1.0, 2.0, 3.0	continuous	one-time	19 (19/19)	earlobe
10.3389/fnins.2021.767302	Chang, J. L., et al., 2021	Stroke	double-blinded, sham-controlled intervention study	cymba conchae	left	MIDI	30	0,1 - 5,0	continuous	3x/weeks 3 weeks	36 (18/18)	no current
10.1007/s10286-024-01053-0.	Cibulcova, V., et al., 2024	Memory	single-blind randomized placebo-wait-list controlled	surface of tragus (inner, outer)	left	Parasym® PK01	25	individual	continuous	4h/day for 2 weeks	78 (44/34)	earlobe
10.1016/j.cortex.2017.03.017	Colzato, L. S., et al., 2017	Emotion	single-blind, sham-controlled, randomized crossover within subjects	outer auditory canal	left	NEMOS	25	5	30s on 30s off	two-times	38 (38/38)	earlobe
10.1111/psyp.13885	D'Agostini, M., et al., 2021	Pupil	single-blinded, randomized, between-subject design	cymba conchae	left	DS5/NEMOS	25	individual	30s on – 30s off	one-time	67 (32/35)	earlobe
10.1111/psyp.14754.	D'Agostini, M., et al., 2025	Cognition	randomized, sham-controlled study	cymba concha	left	DS5/NEMOS	25	Individual (max.4)	continuous	one-time	80 (39/41)	earlobe
10.1111/psyp.14250.	De Smet, S., et al., 2023	Cognition	between-subjects study (not specified)	cymba concha	left	NEMOS®	25	individual	30s on – 30s off	one-time	83 (42/41)	earlobe
10.1186/s12891-025-08288-6	Elsehrawy G. G., et al., 2025	Knee Osteoarthritis	single-blind, sham-controlled, randomized clinical trial	cymba conchae	left	TENS 7000TM	25	individual	continuous	3 days per week for 12 weeks	68 (34/34)	outer earlobe
10.1016/j.nicl.2016.12.016	Fang, J. et al., 2017	Depression	experimental, blinded, double-armed	concha (external ear)	left	Custom	20	4,0 - 6,0	.	2x/day for 5 days/week for 4 weeks	38 (17/21)	superior scapha
10.1016/j.neuroscience.2019.05.059	Finisguerra, A., et al., 2019	Spiritual Representation	cross-over, randomized, sigle-blinded trial	cymba conchae	left	NEMOS	25	0,5	30s on – 30s off	2x90min separeted by 1 week	24 (24/24)	earlobe

Digital Object Identifier	Author, Year, Reference	Outcome parameter	Study design	Stim. region	Stim. side	Stim. device	Fre-quency (Hz)	Intensity (mA)	Stim. block	Study duration	Number of subjects (taVNS/ Sham)	Sham group
10.3758/s13415-018-0596-2	Fischer, R., et al., 2018	EEG P300	randomized, single-blinded, cross-over within-subject design	cymba conchae	left	CM02 Cerbomed	25	individual	continuous	one-time	21 (21/21)	earlobe
10.7717/peerj.14447.	Forte, G., et al., 2022	Heart Rate Variability (HRV)	sham-controlled, single-blinded, randomized crossover within-subject design	cymba concha	left	taVNS L device by tVNS Technologies GmbH	25	individual	30s on - 30s off	2x10min, separated by one week interval	28 (28/28)	helix
10.3390/jcm13144267.	Gianlorenço, A. C., et al., 2024	Heart Rate Variability (HRV)	randomized, double-blind, sham–controlled	auricular concha	left and right	Healaon	30	individual (~1,5)	.	one-time	44 (22/22)	no current
10.3389/fpsyg.2020.01276	Giraudier, M., et al., 2020	Word recognition memory	single-blind, double-armed, randomized between-subject design	cymba conchae	left	Cerbomed GmbH	25	individual	30s on 30s off	one-time	60 (30/30)	earlobe
10.1007/s00406-015-0618-9	Hasan, A., et al., 2015	Schizophrenia	bizonal, randomized, controlled pilot study	external auditory canal	left	CM02, Cerbomed	25	0,1 - 10	30s on – every 3min	3x1h/day, 3x 2h/day, 3x3h/day for 12 weeks	17 (9/8)	no current
10.1186/1472-6882-14-203	Huang, F. et al., 2014	Diabetes mellitus	randomized, controlled pilot study	cymba conchae	.	TENS-200	20	1	continuous	2x/day, 12 weeks	72 (36/36)	scapha (center of outer ear rim)
10.1016/j.neurobiolaging.2015.02.023	Jacobs, H. I., et al., 2015	Memory	single-blinded, sham-controlled pilot study, cross-over design	tragus (inner side)	left	TENSTem dental	8	5	continuous	one-time	30 (30/30)	earlobe
10.1213/ANE.0000000000002820	Janner H., et al., 2018	Acute and chronic pain	prospective, single-blinded, crossover, placebo-and sham-controlled experimental	cymba conchae	bilateral	PuntoBravo (Rostock)	100/2	individual	continuous	4 sessions 25min, 50min, 48h breaks	49 (49/49)	earlobe
10.1155/2020/6049891	Jiao, Y., et al., 2020	Insomnia	multicenter, randomized, clinical trial	auricular concha	bilateral	Hwato brand SDZ-IIB, Suzhou, Jiangsu, China	20	individual	30min	2x30min/day, 5 consecutive days for 4 weeks	72 (36/36)	bilateral auricular scapha

Digital Object Identifier	Author, Year, Reference	Outcome parameter	Study design	Stim. region	Stim. side	Stim. device	Fre-quency (Hz)	Intensity (mA)	Stim. block	Study duration	Number of subjects (taVNS/ Sham)	Sham group
10.3389/fpsyg.2018.01159	Jongkees, B.J., et al., 2018	Response selection during sequential action	single-blind, sham-controlled between-subject design	outer auditory canal	left	CMO2 Cerbomed	25	0,5	30s on – 30s off	one-time	40 (20/20)	earlobe
10.1016/j.autneu.2014.07.008	Laqua, R., et al., 2014	Pain treatment	crossover placebo-controlled (randomized)	auricular concha	bilateral	TNS SM 2 MF	100/2	individual	continuous	two-times	22 (22/22)	earlobe
10.3389/fneur.2023.1286919.	Le Roy, B., et al., 2023	Stress operationality	single-centre, cross-over, interventional, exploratory study	cymba conchae	left	tVNS L device	25	individual	30s on – 30s off	two- or six-times	44 (44/44)	earlobe
10.3389/fneur.2023.1210103.	Lench, D., et al., 2023	Parkinson's disease	double-blind, sham controlled RCT	anterior wall of the left outer ear canal landmarked by the tragus	left	custom fabricated ear clip electrodes	25	individual	60s on – 30s off	1h/day for 10 days	30 (15/15)	earlobe
10.4103/1673-5374.332155	Li, J. N., et al., 2022	Stroke	randomized, controlled study	cymba conchae	left	Suzhou Medical Appliance	20	1,71	30s on – every 5 min	20min/day for 20 days	56 (28/28)	no current
10.1016/j.jad.2024.03.005.	Liu, C., et al., 2024	Post-stroke depression	double-blind, randomized, placebo-controlled trial	concha cavity, cymba concha	left	stimulation therapy instrument (manufactured in Suzhou, China)	20	1,82 ± 0,4	30s on – every 5 min	45min/day for 28 days	80 (40/40)	no current
10.1038/s41598-024-72179-4	Ludwig, M., et al., 2024	Pupil	sham-controlled, single-blind, within-subject, counterbalanced, randomized	cymba conchae	left	tVNS Technologies nextGen research device	10/25	3/5	3s on – 15s off	one-time taVNS	24 (24/24)	earlobe
10.1111/psyp.14753.	Ludwig, M., et al., 2025	Memory and pupil size	sham-controlled, single-blind, within-subject, counterbalanced, randomized	cymba concha	left	tVNS Technologies nextGen research device	10/25	individual	3s on – 15s off	one-time	24 (24/24)	earlobe

Digital Object Identifier	Author, Year, Reference	Outcome parameter	Study design	Stim. region	Stim. side	Stim. device	Fre-quency (Hz)	Intensity (mA)	Stim. block	Study duration	Number of subjects (taVNS/ Sham)	Sham group
10.1016/j.neuropsychologia.2020.107465	Maraver M.J., et al., 2020	Emotion	single-blind, sham/placebo-controlled, randomized cross-over within-subject	cymba conchae	left	NEMOS	25	0,5	30s on-30s off	one time taVNS but 2 sessions for sham and real	43 (43/43)	earlobe
10.3389/fpsyg.2020.00551	Mertens, A., et al., 2020	Memory	randomized, controlled study, cross-over design	cymba conchae	.	NEMOS	25	0,5 / individual	30s	one-time taVNS	65 (65/65)	earlobe
10.3389/fneur.2022.1030927	Paccione, C.E., et al., 2020	Fibromyalgia	single-blinded randomized, sham-controlled trial	cymba conchae	left	NEMOS	25	0,1 to 10, individual	30s on – 30s off	2x/day, 14 days,	57 (28/29)	earlobe
10.3390/brainsci10100668	Ricci, L., et al., 2020	Cerebral cortex activity	randomized sham-controlled double-blind study with cross over	external acoustic meatus	left	Twister-EBM, two Ag-AgCl electrodes	30	individual	30s per train, repeated every 5min	60min	8 (8/8)	earlobe
10.1016/j.jad.2016.02.031	Rong, P. et al., 2016	Depression	experimental, not randomized study	cymba conchae	.	self-developement	20	4,0 - 6,0	continuous	2x/day for 12 weeks	160 (first 91/69, then 160/160)	scapha
10.1186/s42234-023-00124-3.	Sahn, B., et al., 2023	pediatric-onset Crohn's disease (CD) or ulcerative colitis (UC)	phase 1: Randomized, sham-controlled, single-blind, cross-over trial; phase 2: Open-label, single-arm, longitudinal extension	cymba conchae	left	TENS 7000, Roscoe Medical	20	individual	continuous	Phase 1: 5min/day for 2 weeks; Phase 2: 2x 5min/day for 12 weeks	22 (22/22)	calf
10.1016/j.cortex.2017.11.007	Sellaro, R., et al., 2018	Emotion	single-blind, sham-controlled, randomized cross-over within-subject	cymba conchae	left	NEMOS	25	0,5	30s on – 30s off	two-times	24 (24/24)	earlobe
10.1523/jneurosci.1361-20.2020	Sharon, O., et al., 2021	Pupil	randomized study, cross-over design	cymba conchae	left	NEMOS	25	individual	3,4s on, app. 26s off	4x 5min, one-time	24 (24/24)	earlobe

Digital Object Identifier	Author, Year, Reference	Outcome parameter	Study design	Stim. region	Stim. side	Stim. device	Fre-quency (Hz)	Intensity (mA)	Stim. block	Study duration	Number of subjects (taVNS/ Sham)	Sham group
10.14309/ajg.0000000000002548.	Shi, X., et al., 2024	Functional Dyspepsia	multicenter, Randomized Controlled Study	tragus	left	XD-Kerfun BS-VNS-001, Xi'an Kerfun Medical, China	10/25	0.5-1.5	30s on – 30s off	2x 30min/day for 4 weeks	300 (200/100)	earlobe
10.7874/jao.2015.19.3.159	Shim, H. J., et al., 2015	Tinnitus	intervention study	cymba conchae	left	Eco2	20	1,0 -10	continuous	10 times, 30min	30 (30/0)	none
10.1016/j.neuropsychologia.2023.108614.	Sommer, A., et al., 2023	Cognition	single-blinded, sham-controlled, within-subject design	cymba conchae	left	NEMOS®	25	individual	30s on – 30s off	2x90min on two days	32 (32/32)	earlobe
10.1097/MD.0000000000011625	Song. G.F., et al., 2018	Poststroke epilepsy	retrospective, not blinded, intervention and control group on waiting list	auricular concha	bilateral	NEMOS	.	.	continuous	1 session daily, twice weekly, for 4 weeks	52 (27 in treatment group/ 25 on waiting-list)	.
10.1016/j.ijpsycho.2019.10.010	Steenbergen, L., et al., 2020	Emotion	randomized, controlled study, cross-over within-subject Design	cymba conchae	left	NEMOS	25	0,5	30s on - 30s off	one-time	94 (94/94)	earlobe
10.3758/s13415-021-00928-3	Steenbergen, L., et al., 2021	Emotion	single-blind, sham-controlled, randomized cross-over within-subject	cymba conchae	left	NEMOS	25	0,5	30s on-30s off	2xapp.60min seperated by one week	73 (73/73)	earlobe
10.1111/j.1528-1167.2012.03492.x	Stefan, H., et al., 2012	Epilepsy	pilot study (no randomized stimulation)	auricular branch	left	.	10	individual	continuous	3x1h/day for 9 months	10 (10/0)	none
10.1186/s10194-015-0543-3	Straube, A. et al., 2015	Migraine	monocentric, randomized, controlled study	cymba conchae	left	NEMOS	1, 25	individual	30s on - 30s off	4 hours/day for 3 months	42 (1Hz 17, 25Hz 25) (42/0)	control group - 1Hz
10.7554/eLife.100088., correction: Published: 15 January 2025	Tan, G., et al., 2025	Cardiovascular function	randomized sham-controlled, intervention study, double-blind	conchae	left	TENS 7000 Digital TENS Unit	20	0.4	continuous	2x20min/day during stay in ICU	24 (13/11)	earlobe
10.3390/jcm13247601.	Tavares-Figueiredo, I., et al., 2024	Chronic low back pain	Pilot study	cymba concha	left	VAGUSTIM device	25	individual	continuous	three months every day 30min	27 (27/0)	none

Digital Object Identifier	Author, Year, Reference	Outcome parameter	Study design	Stim. region	Stim. side	Stim. device	Fre-quency (Hz)	Intensity (mA)	Stim. block	Study duration	Number of subjects (taVNS/ Sham)	Sham group
10.1016/j.bbr.2022.114164.	Thakkar, V. J., et al., 2023	Memory	pilot study	posterior side of the left tragus	left	TENS7000 device, Parasym device	5	individual	during reading	one-time taVNS -> 12 textpassages (12x stimulation)	24 (12/12)	earlobe
10.1186/s42234-022-00095-x	Tirado, C. F., et al., 2022	OWS (Opioid withdrawal syndrome)	randomized, sham-controlled, double-blind period (30min); 5-day open-label period	cymba concha, anterior to the tragus	left	Sparrow	5/100	average 1.0/1.4	5min on – 10s off	24-hours a day on Days 2–5	31 (16/15)	no current
10.1162/jocn_a_01603	Tona, K. D., et al., 2020	Cognition	single-blind,sham-controlled,crossover design	concha (inner side of ear)	left	NEMOS, Cerbomed	25	0.5/1.0	30s on-30s off	two sessions, scheduled 1 week apart	72 (72/72)	earlobe
10.15562/bmj.v12i1.4017	Uzlifatin, Y., et al., 2023	Chronic low back pain	randomized controlled trial	cymba conchae	left	TENS Myomed 632 device	25	individual	continuous	20min 5x/weeks for 2 weeks	22 (11/11)	none
10.3389/fnhum.2018.00202	Ventura-Bort, C., et al., 2018	EEG und Alpha-Amylase	randomized, blinded, controlled study, cross-over within subject design	cymba conchae	left	CM02 Cerbomed	25	individual	30s on – 30s off	one-time	21 (21/21)	earlobe
10.1002/hbm.26613.	Ventura-Bort, C., et al., 2024	Neurophysiology	a randomized, single-blinded, within-subject, crossover design	cymba conchae	left	CM02, Cerbomed	25	individual	30s on – 30s off	2x15min (+6min), seperated by one week intervall	53 (53/53)	earlobe
10.1007/s00415-022-11465-5.	Wang, Y., et al., 2023	Post-stroke dysphagia	a sham-controlled, double-blinded, parallel pilot study	concha and ear hole	left and right	produced by Xinzhile Medical Co., Ltd, (Jiangxi, China)	25	Individual (0-5mA)	continuous	2x 30min/day for 5 consecutive days a week/for 3 weeks	39 (19/20)	no current
10.3389/fnins.2024.1346634.	Wang, M. H., et al., 2024	Subacute stroke motor function	double-blind, randomized, placebo-controlled pilot trial	cymba conchae	left	tVNS501, RISHENA, Changzhou, China	25	individual	30s on – 30s off	two-times	40 (20/20)	no current

Digital Object Identifier	Author, Year, Reference	Outcome parameter	Study design	Stim. region	Stim. side	Stim. device	Fre-quency (Hz)	Intensity (mA)	Stim. block	Study duration	Number of subjects (taVNS/ Sham)	Sham group
10.1155/2020/8841752	Wu, D., et al., 2020	Stroke	randomized, blinded, sham-controlled, pilot study	cymba conchae	left	BHD-1A	20	individual	30s on – every 5min	30min/day for 15 days	21 (10/11)	no current
10.1016/j.brs.2023.02.008.	Wu, Z., et al., 2023	Inflammation	randomized, double-blind, sham-cotrolled pilot study	tragus and cymba conchae	bilateral	T0138, Xinzhile Medical Device, Incl. Jiangxi, China	25	3	continuous	1x30m/day für 5 days	23 (12/11)	no current
10.1111/cns.14309.	Zhang, H., et al., 2023	Gait and cortical activity	pilot, randomized, double-blind, sham-controlled	cymba conchae	left	tVNS501, RISHENA, Changzhou, China	20	individual	60s on - 10s off	2x30min/day for 7 days	22 (11/11) + 14 healthy controls	no current
10.1016/j.jad.2024.06.083.	Zhang, H., et al., 2024	Cognition	double-blind, randomized, and sham-controlled trial	cymba conchae	left	tVNS501, RISHENA, Changzhou, China	20 for 7s, 4 for 3s	individual	7s (20 Hz) – 3s (4 Hz)	2x 30min/day for 2 weeks	90 (15/15) -> 30 PD without anxiety, 30 healthy controls	earlobe
10.1111/1744-9987.14243.	Zhang, Q., et al., 2025	Hemodialysis	randomized, controlled clinical trial	cymba conchae	left	.	25	individual	continuous	three times per week for 8 weeks	63(32/31)	no current
10.1007/s40520-022-02177-x	Zhou, Q., et al., 2022	Immunology	prospective double-blinded, randomized, controlled study	cymba conchae	left	Roscoe TENS 7000	10	individual	continuous	one-time	119 (60/59)	ear lobe + helix region
10.3389/fnins.2023.1323079.	Zhou, Y., et al., 2023	Minimally conscious state	prospective, single-center, double-blind, shamcontrolled, parallel-group design	specific auricular acupoints including cymba conchae	left	taVNS501, Changzhou Rishena Medical Device Co.	20	Individual	continuous	twice daily, 6 days per week, over a period of 4 weeks	51 (26/25)	no current
10.1152/ajpgi.00426.2020	Zhu, Y., et al., 2021	Functional dyspepsia	randomized, double-blinded, sham-controlled study	cymba concha	left and right	SNM-FDC01	25	0,5 - 1,5	2s on – 3s off	2x 1h/day for two weeks	21 (21/21)	left arm wrist
10.1016/j.ijchp.2024.100462.	Zhu, S., et al., 2024	Emotional inhibitory control	sham-controlled, participant-blind, between-subject design	tragus	left	.	.	individual	continuous	two-times	82 (41/41)	earlobe

Supplementary Table S2

Table 2: Overview of symptom assessment across studies and reported severity levels

Symptom	Number of studies symptom examined	Study	Number of symptom during taVNS	Number of symptom during sham	Mean for symptom on scale (taVNS/sham) * significant higher in taVNS in comparison with sham	Severity level
**Neck pain**	**24**					
		D'Agostini, M., et al., 2021	.	.	(2,63/1,8)	Likert scale 0-6
		D'Agostini, M., et al., 2025	.	.	(0,16/0,33)*	Likert scale 0-6
		De Smet, S., et al., 2023	.	.	(1,17/1,46)	Likert scale 1-5
		Lench, D., et al., 2023	0	1	.	No serious AE
		Sommer, A., et al., 2023	.	.	(1,34/1,59)	Likert scale 1-7
		Tavares-Figueiredo, I., et al., 2024	1	.	.	4 minor, 2 mild (no scales mentioned)
		Ventura-Bort, C., et al., 2024	.	.	(1,55/1,61)	Likert scale 1-7
		Zhu, S., et al., 2024	.	.	(1,05/1,24)	Likert scale 1-7
		Beste, C., et al., 2016	.	.	.	.
		Burger, A. M., et al., 2019	.	.	.	Likert scale 1-5
		Colzato, L. S., et al., 2017	.	.	.	Likert scale 1-5
		Fischer, R., et al., 2018	.	.	(1,47/1,30)	Likert scale 1-7
		Giraudier, M., et al., 2020	.	.	(1,80/1,30)	Likert scale 1-7
		Jacobs, H. I., et al., 2015			(13,5/17) taVNS/90min after taVNS (3/10) sham/ 90min nach sham	Likert scale 0-3
		Sharon, O., et al., 2021	.	.	(1,87/1,97)	Likert scale 0-9
		Steenbergen, L., et al., 2020	.	.	.	Likert scale 1-5

		Tona, K. D., et al., 2020	.	.	.	Likert scale 1-5, all below 2,0
		Ventura-Bort, C., et al., 2018	.	.	(1,5/1,3)	Likert scale 1-7
		Burger, A. M., et al., 2016	.	.	overall: MtVNS= 2.20, Msham=1.90	Likert scale 1-5
		Burger A. M., et al., 2018	.	.	.	Likert scale 1-5
		Jongkees, B.J., et al., 2018	.	.	.	Likert scale 1-5
		Maraver M.J., et al., 2020	.	.	(1,65/1,62)	Likert scale 1-5
		Sellaro, R., et al., 2018	.	.	.	Likert scale 1-5
		Steenbergen, L., et al., 2021	.	.	(1,20/1,15)	Likert scale 1-5
**Headaches**	**23**					
		D'Agostini, M., et al., 2021	.	.	(2,09/1,97)	Likert scale 0-6
		D'Agostini, M., et al., 2025	.	.	(0,3/0,48)	Likert scale 0-6
		De Smet, S., et al., 2023	.	.	(1,12/1,20)	Likert scale 1-5
		Lench, D., et al., 2023	0	1	.	Minor AEs
		Liu, C., et al., 2024	5	4	.	transient
		Ludwig, M., et al., 2025	.	.	(0,08/0,04)	Likert scale 0-3
		Ludwig, M., et al., 2024	.	.	(0,04/0)	Likert scale 0-3
		Sommer, A., et al., 2023	.	.	(1,28/1,44)	Likert scale 1-7
		Ventura-Bort, C., et al., 2024	.	.	(1,47/1,41)	Likert scale 1-7
		Zhang, Q., et al., 2025	2	1	.	.
		Zhu, S., et al., 2024	.	.	(1,66/1,34)	Likert scale 1-7
		Beste, C., et al., 2016	.	.	.	.
		Colzato, L. S., et al., 2017	.	.	.	No major complaints
		Giraudier, M., et al., 2020	.	.	(1,83/2,03)	Likert scale 1-7
		Song. G.F., et al., 2018	3	1	.	No severe AEs
		Tona, K. D., et al., 2020	.	.	.	Likert scale 1-5, all below 2,00
		Ventura-Bort, C., et al., 2018	.	.	(1,5/1,85)	Likert scale 1-7
		Burger A. M., et al., 2018	.	.	Overall MtVNS= 2.20, Msham= 1.90	Likert scale 1-5

		Maraver M.J., et al., 2020	.	.	0,62-2,65, no significant differences	Likert scale 1-5
		Sellaro, R., et al., 2018	.	.	.	Likert scale 1-5<br>No major AEs
		Steenbergen, L., et al., 2021	.	.	(1,32/1,37)	Likert scale 1-5
		Stefan, H., et al., 2012	1	.	.	.
		Jiao, Y., et al., 2020	1	0	.	.
**Nausea**	**22**					
		D'Agostini, M., et al., 2021	.	.	(1,53/1,09)*	Likert scale 0-6
		D'Agostini, M., et al., 2025	.	.	(0,03/0,2)	Likert scale 0-6
		Ludwig, M., et al., 2025	.	.	(0,04/0)	Likert scale 0-3
		Ludwig, M., et al., 2024	.	.	(0,04/0)	Likert scale 0-3
		De Smet, S., et al., 2023	.	.	(1,15/1,07)	Likert scale 1-5
		Lench, D., et al., 2023	1	1	.	Minor AEs
		Liu, C., et al., 2024	7	8	.	transient
		Sommer, A., et al., 2023	.	.	(1,00/1,03)	Likert scale 1-7
		Ventura-Bort, C., et al., 2024	.	.	(1,27/1,01)	Likert scale 1-7
		Zhu, S., et al., 2024	.	.	(1,07/1,22)	Likert scale 1-7
		Beste, C., et al., 2016	.	.	.	.
		Colzato, L. S., et al., 2017	.	.	.	No major complaints
		Giraudier, M., et al., 2020	.	.	(1,17/1,30)	Likert scale 1-7
		Song. G.F., et al., 2018	2	0	.	No severe AEs
		Tona, K. D., et al., 2020	.<br>.	.	.	Likert scale 1-5
		Burger A. M., et al., 2018	.	.	overall: MtVNS= 2.20/ Msham=1.90	Likert scale 1-5
		Maraver M.J., et al., 2020	.	.	(1,25/1,25)	Likert scale 1-5
		Sellaro, R., et al., 2018	.	.	.	No major complaints<br>Likert scale 1-5
		Steenbergen, L., et al., 2021	.	.	(1,17/1,10)	Likert scale 1-5

		Ventura-Bort, C., et al., 2018	.	.	(1,2/1,23)	Likert scale 1-7
		Jongkees, B.J., et al., 2018	.	.	.	Likert scale 1-5
		Zhou, Q., et al., 2022	18	16	.	.
**Malaise (unspecific)**	**21**					
		D'Agostini, M., et al., 2021	.	.	(2,91/2,06)*	Likert scale 0-6
		D'Agostini, M., et al., 2025	.	.	(1,62/1,52)	Likert scale 0-6
		Sommer, A., et al., 2023	.	.	(1,47/1,47)	Likert scale 1-7
		Tavares-Figueiredo, I., et al., 2024	.	.	.	No scales mentioned
		Ventura-Bort, C., et al., 2024	.	.	(1,73/1,59)	Likert scale 1-7
		Zhu, S., et al., 2024	.	.	(2,07/1,95)	Likert scale 1-7
		Beste, C., et al., 2016	.	.	.	.
		Busch, V., et al., 2013	1	0	.	.
		Colzato, L. S., et al., 2017	.	.	.	No major complaints
		Fischer, R., et al., 2018	.	.	(2,15/1,81)	Likert scale 1-7
		Giraudier, M., et al., 2020	.	.	(1,67/2,23)	Likert scale 1-7
		Sharon, O., et al., 2021	.	.	(2,52/2,71)	Likert scale 0-9
		Steenbergen, L., et al., 2020	.	.	.	Likert scale 1-5
		Tona, K. D., et al., 2020	.	.	(2,0/2,1)<br>0.5mA/1.0mA real<br>(1,6/1,7)<br>0.5mA/1.0mA sham	Likert scale 1-5
		Ventura-Bort, C., et al., 2018	.	.	(1,94/1,65)	Likert scale 1-7
		Burger A. M., et al., 2018	.	.	.	Likert scale 1-5
		Burger, A. M., et al., 2016	.	.	.	Likert scale 1-5
		Jongkees, B.J., et al., 2018	.	.	.	5 Likert scale 1-5<br>No major side effects
		Maraver M.J., et al., 2020	.	.	(2,21/2,09)	Likert scale 1-5
		Sellaro, R., et al., 2018	.	.	.	Likert scale 1-5
		Steenbergen, L., et al., 2021	.	.	(2,38/2,18)	Likert scale 1-5

**Facial and / or neck muscle cramps**	**18**					
		De Smet, S., et al., 2023	.	.	(1,37/1,32)	Likert scale 1-5
		Sommer, A., et al., 2023	.	.	(1,22/1,5)	Likert scale 1-7
		Zhu, S., et al., 2024	.	.	(1,59/1,56)	Likert scale 1-7
		Beste, C., et al., 2016	.	.	.	Consultation only, no examination
		Burger, A. M., et al., 2019	.	.	.	Likert scale 1-5
		Colzato, L. S., et al., 2017	.	.	.	No major complaints
		Fischer, R., et al., 2018	.	.	(1,62/1,70)	Likert scale 1-7
		Giraudier, M., et al., 2020	.	.	(2,27/1,93)	Likert scale 1-7
		Sharon, O., et al., 2021	.	.	(2,10/2,00)	Likert scale 1-9
		Steenbergen, L., et al., 2020	.	.	.	Likert scale 1-5
		Tona, K. D., et al., 2020	.	.	(<2.0/<2.0)	Likert scale 1-5
		Burger A. M., et al., 2018	.	.	.	Likert scale 1-5
		Burger, A. M., et al., 2016	.	.	.	Likert scale 1-5
		Jongkees, B.J., et al., 2018	.	.	.	Likert scale 1-5<br>No major side effects
		Maraver M.J., et al., 2020	.	.	(1,83/1,51)	Likert scale 1-5
		Sellaro, R., et al., 2018	.	.	.	Likert scale 1-5
		Steenbergen, L., et al., 2021	.	.	(1,30/1,18)	Likert scale 1-5
		Ventura-Bort, C., et al., 2024	.	.	(1,93/2,05)	Likert scale 1-7
**Dizziness**	**16**					
		Huang, F. et al., 2014	1	1	.	Both dropped out
		Lench, D., et al., 2023	1	0	.	Minor AEs
		Liu, C., et al., 2024	3	4	.	Transient
		Ludwig, M., et al., 2025	.	.	(0,04/0,04)	Likert scale 0-3
		Sommer, A., et al., 2023	.	.	(1,16/1,22)	Likert scale 1-7
		Ventura-Bort, C., et al., 2024	.	.	(1,49/1,43)	Likert scale 1-7
		Zhang, Q., et al., 2025	1	1	.	.
		Zhu, S., et al., 2024	.	.	(1,54/1,71)	Likert scale 1-7

		Aihua, L., et al., 2014	1	0	.	severe, 1 drop-out (taVNS)
		Fischer, R., et al., 2018	.	.	(1,71/1,67)	Likert scale 1-7
		Giraudier, M., et al., 2020	.	.	(1,67/2,27)	Likert scale 1-7
		Jacobs, H. I., et al., 2015	.	.	(33/0) taVNS/90min nach taVNS () (6/6) Sham/90min nach Sham)	Likert scale 0-3
		Sharon, O., et al., 2021	.	.	(1,82/1,97)	Likert scale 0-9
		Song. G.F., et al., 2018	3	1	.	No severe AEs
		Ventura-Bort, C., et al., 2018	.	.	(1,55/1,7)	Likert scale 1-7
		Sellaro, R., et al., 2018	.	.	.	Likert scale 1-5
**Burning of the skin**	**15**					
		D'Agostini, M., et al., 2021	.	.	(3,19/1,69)*	Likert scale 0-6
		D'Agostini, M., et al., 2025	.	.	(2,0/0,73)*	Likert scale 0-6
		De Smet, S., et al., 2023	.	.	(1,90/1,44)	Likert scale 1-5
		Sommer, A., et al., 2023	.	.	(1,06/1,16)	Likert scale 1-7
		Beste, C., et al., 2016	.	.	.	.
		Burger, A. M., et al., 2019			(2,5/1,0)*	Likert scale 1-5
		Colzato, L. S., et al., 2017	.	.	.	No major complaints
		Steenbergen, L., et al., 2020	.	.	(2,08/1,62)*	Likert scale 1-5
		Burger A. M., et al., 2018	.	.	Overall: MtVNS=2.20, Msham= 1.90	Likert scale 1-5
		Burger, A. M., et al., 2016	.	.	.	Not significant more during taVNS, Likert scale 1-5
		Jongkees, B.J., et al., 2018	.	.	.	Not significant more during taVNS, Likert scale 1-5
		Maraver M.J., et al., 2020	.	.	.	0,62-2,65 (Likert scale from 1-5)
		Ricci, L., et al., 2020	.	.	.	mild

		Sellaro, R., et al., 2018	.	.	.	5 Likert scale 1-5<br>No major complaints
		Steenbergen, L., et al., 2021	.	.	(2,27/2,27)	5 Likert scale 1-5
**Tingling sensation**	**14**					
		Camargo, L., et al., 2024	1	.	.	Mild
		D'Agostini, M., et al., 2025	.	.	(2,95/2,9)	Likert scale 0-6
		De Smet, S., et al., 2023	.	.	(2,00/2,07)	Likert scale 1-5
		Ludwig, M., et al., 2024	.	.	(0,38/0,29)	Likert scale 0-3
		Ludwig, M., et al., 2025	.	.	(0,04/0)	Likert scale 0-3
		Gianlorenço, A. C., et al., 2024	1	0	.	No serious AEs
		Zhang, Q., et al., 2025	1	2	.	.
		Burger, A. M., et al., 2019	4	3	.	.
		Busch, V., et al., 2013	12	2	.	.
		Elsehrawy G. G., et al., 2025	.	.	.	55 overall, mild, tolerable
		Fischer, R., et al., 2018				Likert scale 1-7
		Jacobs, H. I., et al., 2015	.	.	.	Likert scale 0-3
		Tona, K. D., et al., 2020	.	.	(3,1/2,7/2,4)<br>(1mA/0,5mA/sham)	Likert scale 1-5
		Burger A. M., et al., 2018	.	.	(Overall: MtVNS= 2,20, Msham=1,90)	Likert scale 1-5
**Stinging sensation**	**13**					
		Ventura-Bort, C., et al., 2024	.	.	(2,4/2,13)	Likert scale 1-7
		Zhu, S., et al., 2024	.	.	(3,22/3,71)	Likert scale 1-7
		Burger, A. M., et al., 2019	.	.	(4,0/3,0)*	Likert scale 1-5
		Colzato, L. S., et al., 2017	.	.	.	No major complaints
		Ventura-Bort, C., et al., 2018	.	.	(2,87/1,95)*	Likert scale 1-7
		Giraudier, M., et al., 2020	.	.	(2,87,2,80)	Likert scale 1-7
		Jongkees, B.J., et al., 2018	.	.	.	Likert scale 1-5<br>No major AEs
		Maraver M.J., et al., 2020	.	.	(2,62/2,65)	Likert scale 1-5

		Sellaro, R., et al., 2018	.	.	.	Likert scale 1-5No major AEs
		Steenbergen, L., et al., 2021	.	.	(3,42/3,35)	Likert scale 1-5
		Beste, C., et al., 2016	.	.	.	.
		Fischer, R., et al., 2018	.	.	(3,00/2,05)*	Likert scale 1-7
		Sharon, O., et al., 2021	.	.	(2,22/2,18)	Likert scale 0-9
**Changes in concentration**	**12**					
		Lench, D., et al., 2023	0	1	.	Minor AEs
		Ludwig, M., et al., 2025	.	.	(0,5/0,5)	Likert scale 0-3
		Ludwig, M., et al., 2024	.	.	(0,58/0,46)	Likert scale 0-3
		Sommer, A., et al., 2023	.	.	(1,97/1,75)	Likert scale 1-7
		Fischer, R., et al., 2018	.	.	(3,71/3,52)	Likert scale 1-7
		Giraudier, M., et al., 2020	.	.	(2,97/3,47)	Likert scale 1-7
		Jacobs, H. I., et al., 2015	.	.	(3/23) taVNS/90min after taVNS (6/30) Sham/90min after Sham	.
		Sharon, O., et al., 2021	.	.	(4,02/3,73)	Likert scale 0-9
		Ventura-Bort, C., et al., 2018	.	.	(3,55/3,45)	Likert scale 1-7
		D'Agostini, M., et al., 2025	.	.	(43,89/31,2)*	Distraction on VAS 0-1000
		D'Agostini et al 2021	.	.	(21.84/6.34)*	
		Ventura-Bort, C., et al., 2024	.	.	(2,8/2,93)	Likert scale 1-7
**Unpleasant skin sensation**	**11**					
		Tirado, C. F., et al., 2022	1	.		Mild
		Sommer, A., et al., 2023	.	.	(1,41/1,53)	Likert scale 1-7
		Ventura-Bort, C., et al., 2024	.	.	.	Low
		Elsehrawy G. G., et al., 2025	60 participants (88%)	.	.	Mild

		Fischer, R., et al., 2018	.	.	(2,00/1,3)*	Likert scale 1-7
		Giraudier, M., et al., 2020	.	.	(1,77/1,93)	Likert scale 1-7
		Hasan, A., et al., 2015	2	4	.	no severe AEs
		Jacobs, H. I., et al., 2015			(3,5/10) taVNS/90min after taVNS (20/3) sham/ 90min after sham	Likert scale 0-3
		Sharon, O., et al., 2021			(2,17/2,15)	Likert scale 0-9
		Ventura-Bort, C., et al., 2018			(1,8/1,15)*	Likert scale 1-7
		Ackland, G. L., et al., 2025	.	.	.	.
**Cardiovascular symptoms (e.g. palpitation, weakness, collapse)**	**9**					
		Zhang, Q., et al., 2025	1	0	.	No serious AEs
		Hasan, A., et al., 2015	.	.	HF higher on the end of study during sham *	.
		Zhou, Q., et al., 2022	0 HR <50/min (7) or >120/min (7), 0 BD <90mmHg (8), >180mmHg (17)	0 HR <50/min (5) or >120/min (10), 0 BD <90mmHg (11), >180mmHg (20)	.	No significant differences
		Tirado, C. F., et al., 2022	.	.	.	significant reduction in HR und R-R interval
	Orthostatic hypotension	Lench, D., et al., 2023	1	1	.	No serious AEs
	Hypertension	Tavares-Figueiredo, I., et al., 2024	2	.	.	minor
	Sinus Bradycardia	Zhou, Y., et al., 2023	1 (sinus bradycardia) 5 (abnormal blood pressure)	0 (sinus bradycardia) 7 (abnormal blood pressure)	.	.
		Laqua, R., et al., 2014	1 circulatory collapse due to	.	.	.

			bradycardia and hypotension			
	Palpitation	Shi, X., et al., 2024	1	0	.	Mild
**Fatigue**	**8**					
		Lench, D., et al., 2022	2	1	.	minor
		Liu, C., et al., 2024	3	2	.	.
		Zhang, Q., et al., 2025	0	1	.	No serious AEs
		Song. G.F., et al., 2018	2	1	.	No severe AEs
	drowsiness	D'Agostini, M., et al., 2025	.	.	(0,65/1,32)*	Likert scale 1-7
		Ludwig, M., et al., 2025	.	.	(0,67/0,83)	Likert scale 0-3
		Ludwig, M., et al., 2024	.	.	(1,17/1)	Likert scale 0-4
		Aihua, L., et al., 2014	3	0	.	.
**Fluctuation of feelings**	**8**					
	Butterflies in stomach	D'Agostini et al 2021	.	.	(1,78/1,06)*	Likert scale 1-7
	Jitteriness/ anxiousness	Lench, D., et al., 2023	1	0	.	minor
		Shi, X., et al., 2024	.	.	(1,28/1,22)	Likert scale 1-7
		Ventura-Bort, C., et al., 2024	.	.	(1,58/1,29)	Likert scale 1-7
		Fischer, R., et al., 2018	.	.	(1,85/1,54)	Likert scale 1-7
		Giraudier, M., et al., 2020	.	.	(1,53/1,73)	Likert scale 1-7
		Jacobs, H. I., et al., 2015	.	.	3/0) taVNS/90min after taVNS (0/0) Sham/90min after Sham	
		Ventura-Bort, C., et al., 2018	.	.	(1,65/1,37)	Likert scale 1-7
**Ear discomfort (e.g. feeling of fullness, pressure)**	**7**					
	pain	Song. G.F., et al., 2018	3	1	.	No severe AEs occurred
	pain	Ricci, L., et al., 2020	.	.	.	mild

		Cibulcova, V., J. Koenig, M. Jackowska and V. K. Jandackova (2024	16	10	average 3,21	Pain scale 1-10, Light
	Decreased hearing	Lench, D., et. Al.,2023	0	1	.	minor
	Ear irritation	Ventura-Bort, C., et al., 2024	.	.	(1,71/1,27)*	Likert scale 1-7
	Ear irritation	Giraudier, M., et al., 2020	.	.	(1,77/1,93)	Likert scale 1-7
	Ear irritation	Zhu, S., et al., 2024	.	.	(2,22/1,73)*	Likert scale 1-7
**Itching**	**7**					
		D'Agostini, M., et al., 2025	.	.	(1,68/1,28)	Likert 0-6
		Busch, V., et al., 2013	10 itching<br>7 tickling	1 itching<br>2 tickling	.	.
		Ricci, L., et al., 2020	.	.	.	mild
		Ludwig, M., et al., 2025	.	.	(0,04/0)	Likert scale 0-3
		Ludwig, M., et al., 2024	.	.	(0/0,04)	Likert scale 0-4
		Zhu, S., et al., 2024	.	.	(2,76/2,83)	Likert scale 1-7
		Sharon, O., et al., 2021	.	.	(4,07/4,15)	Likert scale 0-9
**Skin redness**	**5**					
		Wu, D., et al., 2020	1	.	.	No rating
		Li, J. N., et al., 2022	2	.	.	no major side effects
		D'Agostini, M., et al., 2025	.	.	(0,4/0,05)	Likert scale 0-6
		Sahn, B., et al., 2023	1	0	.	No serious AEs
		Straube, A. et al., 2015	.	.	.	mild/moderate/severe as scale
**Tinnitus / ear noises**	**5**					
		Zhu, Y., et al., 2021	1	.	.	mild
		Le Roy, B., et al., 2023	1	0	.	.
		Shi, X., et al., 2024	3	0	.	mild
		Lench, D., et al., 2023	1	0	.	minor
		Rong, P. et al., 2016	2	3	.	mild
**Arousal**	**4**					
		D'Agostini et al 2021	.	.	(3,34/2,0)*	Likert scale 1-7

		D'Agostini, M., et al., 2025	.	.	(3,811/2,9)*	Likert scale 1-7
		Zhu, S., et al., 2024	.	.	(2,76/2,83)	Likert scale 1-7
		Sharon, O., et al., 2021	.	.	(4,07/4,15)	Likert scale 0-9
**Skin pain**	**4**					
		Hasan, A., et al., 2015	2	4	.	No severe AEs (see "unpleasant skin sensation")
		Janner H., et al., 2018	.	1	.	1 Drop-out
		Straube, A. et al., 2015	.	.	.	Mild or moderate
		Jiao, Y., et al., 2020	2	1	.	.
**Insomnia**	**3**					
		Lench, D., et. Al.,2023	3	1	.	minor
		Liu, C., et al., 2024	6	7	.	transient
		Shi, X., et al., 2024	1	1	.	mild
**Pain**	**3**	D'Agostini, M., et al., 2025	.	.	(2,29/1,2)*	Likert scale 1-7
		Busch, V., et al., 2013	2	1	.	leicht
		Straube, A. et al., 2015	101	.	.	Mild/ moderat
**Stomach pain**	**3**					
		Lench, D., et al., 2023	1	0	.	No serious AEs
		Liu, C., et al., 2024	4	5	.	Transient
		Shi, X., et al., 2024	1	0	.	mild
**Constipation**	**3**					
		Lench, D., et al., 2023	1	0	.	minor
		Stefan, H., et al., 2012	1	.	.	.
		Zhou, Y., et al., 2023	8	11	.	.
**Pleasant feelings**	**2**					
		D'Agostini et al 2021	.	.	(2,59//1,74)*	Likert scale1-7
		Sharon, O., et al., 2021	.	.	(4,82/5,23)	Likert scale 0-9
**Numbness of a limb**	**2**					

		D'Agostini, M., et al., 2025	8	6	.	Likert scale 0-6
		Le Roy, B., et al., 2023	1	0	.	1 dropped out, no third session
**Vomiting**	**2**					
		Liu, C., et al., 2024	4	3	.	transient
		Song. G.F., et al., 2018	1	0	.	No severe
**Skin pressure sensation**	**1**	Busch, V., et al., 2013	8	6	.	transient
**Feeling of heat**	**1**	Ludwig, M., et al., 2025	.	.	(0,21/0,21)	
**Chest pain**	**1**	Paccione, C.E., et al., 2020	1	.	.	1 dropped out
**Diarrhea**	**1**	Liu, C., et al., 2024	4	5	.	transient
**Dry mouth**	**1**	Liu, C., et al., 2024	3	3	.	Transient
**Dysphagia**	**1**	Busch, V., et al., 2013	1	0	.	transient
**Epistaxis**	**1**	Tavares-Figueiredo, I., et al., 2024	1	.	.	No scales mentioned
**Fluid in the air**	**1**	Lench, D., et al., 2023	1	0	.	Minor AEs
**Grinding teeth**	**1**	Lench, D., et al., 2023	1	0	.	minor
**Happy feelings**	**1**	D'Agostini et al 2021	.	.	((2,97/2,09)*	Likert scale 1-7
**Hidrosis**	**1**	Liu, C., et al., 2024	3	3	.	transient
**Hip pain**	**1**	Lench, D., et al., 2023	1	0	.	Minor AEs
**Hoarseness**	**1**	Stefan, H., et al., 2012	1	.	.	.
**Light-headedness**	**1**	Lench, D., et al., 2023	1	2	.	Minor AEs
**Rush of blood to temples**	**1**	Le Roy, B., et al., 2023	1	0	.	minor
**Sexual dysfunction**	**1**	Liu, C., et al., 2024	5	7	.	Transient
**Slight headfullness**	**1**	Jiao, Y., et al., 2020	1	0	.	.

Symptoms investigated in only one study and explicitly not attributed to the stimulation: infection recurrence, worsening disease activity (CED), Fall with radius fracture, Psoriasis flair up, deep vein thrombosis, pulmonary embolism, seizure, PSH, hydrozephalus, pneumonitis, bladder infection, gastric hemorrhage, alanine aminotransferase increased, anemia, coagulant abnormalities, electrolyte disturbance, hypoalbuminemia

Supplementary Table S3

Table 3: Frequency of symptoms documented during the taVNS sessions (111 questionnaires)

Symptom	Condition	Number of symptoms	Most frequent severity level	Average severity level	Most frequent start time point	Average duration (in min)
**Tingling sensation**	3.4s Stimulation	17	mild	1.29	beginning	9.19
**Tingling sensation**	3.4s Placebo	15	mild	1.27	beginning	7.91
**Tingling sensation**	30s Stimulation	12	mild	1.17	middle	14.85
**Tingling sensation**	30s Placebo	16	mild	1.19	beginning	17.55
**Tingling sensation**	Total	60	mild	1.23	beginning	12.34
**Fatigue**	3.4s Stimulation	16	moderate	1.62	end	10.64
**Fatigue**	3.4s Placebo	12	mild	1.33	end	12.79
**Fatigue**	30s Stimulation	15	mild	1.47	end	14.12
**Fatigue**	30s Placebo	14	moderate	1.93	middle	11.77
**Fatigue**	Total	57	mild	1.6	end	12.32
**Changes in concentration**	3.4s Stimulation	10	mild	1.2	middle	7.44
**Changes in concentration**	3.4s Placebo	7	mild	1.14	end	11.67
**Changes in concentration**	30s Stimulation	11	mild	1.36	end	16.85
**Changes in concentration**	30s Placebo	11	mild	1.45	middle	9.8
**Changes in concentration**	Total	39	mild	1.31	middle	11.65
**Headaches**	3.4s Stimulation	4	mild	1.25	middle	16.25
**Headaches**	3.4s Placebo	2	mild	1.5	middle	17.5
**Headaches**	30s Stimulation	6	mild	1.33	beginning	15
**Headaches**	30s Placebo	4	mild	1.5	middle	17
**Headaches**	Total	16	mild	1.38	middle	16.12
**Itching**	3.4s Stimulation	3	mild	1.33	beginning	11.9
**Itching**	3.4s Placebo	1	mild	1	end	10
**Itching**	30s Stimulation	4	mild	1	beginning	5.88
**Itching**	30s Placebo	2	mild	1	middle	3.5
**Itching**	Total	10	mild	1.1	middle	7.62
**Neck pain**	3.4s Stimulation	4	mild	1.25	middle	18.33
**Neck pain**	3.4s Placebo	2	mild	1	beginning	40
**Neck pain**	30s Stimulation	2	mild	1	end	11
**Neck pain**	30s Placebo	2	mild	1	beginning	30
**Neck pain**	Total	10	mild	1.1	end	24.11
**Ear discomfort**	3.4s Stimulation	2	mild	1	beginning	1.12
**Ear discomfort**	3.4s Placebo	3	mild	1	beginning	7.33
**Ear discomfort**	30s Stimulation	1	mild	1	end	20
**Ear discomfort**	30s Placebo	1	mild	1	middle	2
**Ear discomfort**	Total	7	mild	1	end	6.61
**Skin pressure sensation**	3.4s Stimulation	2	mild	1.5	middle	16.5
**Skin pressure sensation**	3.4s Placebo	1	mild	1	beginning	10
**Skin pressure sensation**	30s Stimulation	2	mild	1.5	beginning	10.5

**Skin pressure sensation**	30s Placebo	1	moderate	2	middle	30
**Skin pressure sensation**	Total	6	mild	1.5	beginning	15.67
**Dizziness**	3.4s Stimulation	1	mild	1	beginning	1
**Dizziness**	3.4s Placebo	1	mild	1	middle	2
**Dizziness**	30s Stimulation	0				
**Dizziness**	30s Placebo	2	mild	1	beginning	1
**Dizziness**	Total	4	mild	1	beginning	1.25
**Tinnitus/Ear noises**	3.4s Stimulation	0				
**Tinnitus/Ear noises**	3.4s Placebo	2	mild	1	middle	1.75
**Tinnitus/Ear noises**	30s Stimulation	0				
**Tinnitus/Ear noises**	30s Placebo	1	mild	1	middle	5
**Tinnitus/Ear noises**	Total	3	mild	1	middle	2.83
**Burning of the skin**	3.4s Stimulation	1	mild	1	middle	5
**Burning of the skin**	3.4s Placebo	0				
**Burning of the skin**	30s Stimulation	0				
**Burning of the skin**	30s Placebo	2	mild	1	beginning	0.2
**Burning of the skin**	Total	3	mild	1	beginning	2.6
**Skin pain**	3.4s Stimulation	1	mild	1	beginning	5.7
**Skin pain**	3.4s Placebo	1	moderate	2	beginning	10
**Skin pain**	30s Stimulation	1	mild	1	beginning	2
**Skin pain**	30s Placebo	0				
**Skin pain**	Total	3	mild	1.33	beginning	5.9
**Muscle cramps**	3.4s Stimulation	1	mild	1	middle	2
**Muscle cramps**	3.4s Placebo	0				
**Muscle cramps**	30s Stimulation	1	mild	1	middle	2
**Muscle cramps**	30s Placebo	0				
**Muscle cramps**	Total	2	mild	1	middle	2
**Breathing difficulties**	3.4s Stimulation	0				
**Breathing difficulties**	3.4s Placebo	0				
**Breathing difficulties**	30s Stimulation	1	moderate	2	middle	3
**Breathing difficulties**	30s Placebo	1	moderate	2	end	2
**Breathing difficulties**	Total	2	moderate	2	middle	2.5
**Malaise**	3.4s Stimulation	0				
**Malaise**	3.4s Placebo	1	moderate	2	middle	2
**Malaise**	30s Stimulation	0				
**Malaise**	30s Placebo	0				
**Malaise**	Total	1	moderate	2	middle	2
**Chest pain**	3.4s Stimulation	0				
**Chest pain**	3.4s Placebo	1	mild	1	middle	2.5
**Chest pain**	30s Stimulation	0				

**Chest pain**	30s Placebo	0				
**Chest pain**	Total	1	mild	1	middle	2.5
**Skin redness**	3.4s Stimulation	0				
**Skin redness**	3.4s Placebo	1	mild	1	after stimulation	1.5
**Skin redness**	30s Stimulation	0				
**Skin redness**	30s Placebo	0				
**Skin redness**	Total	1	mild	1	after stimulation	1.5
**Unpleasant skin sensation**	3.4s Stimulation	0				
**Unpleasant skin sensation**	3.4s Placebo	0				
**Unpleasant skin sensation**	30s Stimulation	1	mild	1	beginning	1
**Unpleasant skin sensation**	30s Placebo	0				
**Unpleasant skin sensation**	Total	1	mild	1	beginning	1
**Swallowing difficulties**	3.4s Stimulation	0				
**Swallowing difficulties**	3.4s Placebo	1	mild	1	beginning	2
**Swallowing difficulties**	30s Stimulation	0				
**Swallowing difficulties**	30s Placebo	0				
**Swallowing difficulties**	Total	1	mild	1	beginning	2

Supplementary Table S4

Table 4: Distribution of reported symptoms per participant across stimulation sessions during taVNS (111 questionnaires)

Condition	Number of subjects	Number of subjects without symptoms (%)	Number of subjects ≥ 1 symptom (%)	Minimum	Maximum	Median	Mean	Standard Deviation
**3.4s Stimulation**	26	4 (15.38)	22 (84.62)	0	6	3	2.46	1.73
**3.4s Placebo**	28	6 (21.43)	22 (78.57)	0	4	2	1.82	1.42
**30s Stimulation**	29	4 (13.79)	25 (86.21)	0	5	2	2.03	1.35
**30s Placebo**	28	5 (17.86)	23 (82.14)	0	6	2	2.18	1.72
**Total**	111	19 (17.12)	92 (82.88)	0	6	2	2.12	1.55

Supplementary Table S5

Table 5: Frequency of reported symptoms during taVNS sessions (111 questionnaires) and participant-rated impairment

Symptom	Number of symptoms	Most severely impairing (%)
**Fatigue**	57	34 (59.65)
**Tingling sensation**	60	19 (31.67)
**Changes of concentration**	39	18 (46.15)
**Headaches**	16	5 (31.25)
**Neck pain**	10	3 (30.00)
**Itching**	10	2 (20.00)
**Skin pressure sensation**	6	1 (16.67)
**Skin pain**	3	1 (33.33)
**Dizziness**	4	1 (25.00)
**Ear discomfort**	7	1 (14.29)
**Malaise**	1	1 (100.00)
**Skin redness**	1	0 (0.00)
**Burning of the skin**	3	0 (0.00)
**Unpleasant skin sensation**	1	0 (0.00)
**Breathing difficulties**	2	0 (0.00)
**Muscle cramps**	2	0 (0.00)
**Swallowing difficulties**	1	0 (0.00)
**Chest pain**	1	0 (0.00)
**Tinnitus/Ear noises**	3	0 (0.00)
**Total**	227	86 (37.89)

Supplementary Table S6

Table 6: Participant-reported symptoms with onset und duration during the taVNS sessions (111 questionnaires)

Subject	Condition	Symptom	Severity level	Start time point	Duration (in min)
1	30s placebo	reduced responsiveness	moderate	beginning	23,5
2	30s placebo	warm sensation of the ear	mild	beginning	2,5
2	30s stimulation	warm sensation of the ear	moderate	middle	2,5
2	3.4s stimulation	warm sensation of the ear	mild	after stimulation	2,5
3	30s placebo	strained eyes	moderate	end	10
3	3.4s stimulation	strained eyes	mild	middle	20
4	30s stimulation	irritated eyes	moderate	end	12
4	30s placebo	irritated eyes	moderate	end	10

Supplementary Table S7

Table 7: Documentation of reasons for stimulation intensity reduction during taVNS sessions (111 questionnaires)

Condition	Primary stimulation intensity (in mA)	Secondary stimulation intensity (in mA)	Minute of transition	Reason
**30s Stimulation**	0,4	0,3	10	Impaired concentration, tingling sensation
**3.4s Stimulation**	0,4	0,2	1	Itching, pain
**3.4s Stimulation**	0,4	0,35	12	Severe intensity distracting
**3.4s Stimulation**	0,4	0,35	4	Too strong
**30s Placebo**	0,35	0,3		Too strong
**30s Placebo**	0,85	0,8	12	Too strong

Supplementary Table S8

Table 8: Comparison of the newly developed semi-structured taVNS-AEQ 3.0 with existing instruments

	taVNS-AEQ 3.0	Kim et al. (2022)	Gerges et al. (2024)	Giraudier et al. (2025)
**Item generation**	**Development**: Targeted literature review, cognitive testing, pilot testing; Version 3.0<br><br>**Item number**: 25 and others	**Development**: Systematic review and meta-analysis<br><br>**Item number**: 10 and others	**Development**: Scoping review<br><br>**Item number**: 10 and others	**Development**: adapted from a questionnaire used in another study, no methodological development process reported<br>**Item number**: 10
**Symptom domains**	local cutaneous, otologic, musculoskeletal, neurological, general systematic and neurovegetative symptoms, cardiothoracic, gastrointestinal	local cutaneous, otologic, neurological, general systematic and neurovegetative symptoms	local cutaneous, otologic, neurological, general systematic and neurovegetative symptoms, gastrointestinal	local cutaneous, musculoskeletal, neurological, general systematic and neurovegetative symptoms, gastrointestinal
**Open-ended vs predefined symptom assessment**	Both, first open question then predefined symptoms	Predefined symptoms with an open-ended "other" option	Predefined symptoms with an open-ended "other" option	Predefined symptoms

**Participant vs investigator report**	Both	Participant	Participant	Participant
**Assessment of symptom severity**	Rating scale, 1-3	Rating scale, 1-4	Rating scale, 1-4	Rating scale, 1-7
**Assessment of symptom onset/timing**	Start time (beginning, middle, end, after study)	Open question, free-text answer	-	-
**Assessment of symptom duration**	Duration (<10, 11-25, 26-49, 50min, only during elec. Stim.)	Open question, free-text answer	Open question, free-text answer	-
**Assessment of symptom persistence**	Still present or symptom disappeared	Open question, free-text answer	Still present or symptom disappeared	Questionnaire answered again 90min after session
**Assessment of symptom impairment**	Strong impairing yes or no	-	-	-
**Assessment of symptom relatedness to stimulation**	Potential reason of symptom, one answer required (0,1,2 or 3)	Relatedness of symptom (1,2,3,4 or 5)	Relatedness, yes or no	-
**Assessment of pre-existing conditions**	Pre-existing medical condition asked	Open question if symptom had before, free-text answer	-	-
**Response format**	Primarily categorical response format, minimal use of free-text answers	3 of 5 questions free-text answers, 2 of 5 categorical responses	Only categorical responses	Only rating scales if and to what extent symptoms experienced
**Total number of questions**	4 for participant, 6 for study coordinator	5 questions for participants	3 questions for participants	1 question for participants
**Language**	English, German, Spanish, Italian, French	English	English	English

Supplementary Material 9

Questionnaire’s questions should be kept the same for comparability, but some aspects need to be adjusted depending on the study – e.g., Stimulation condition (3.4s/30s).

9.1: TaVNS-AEQ – English version 3.0

Questionnaire on adverse events of transcutaneous auricular vagus nerve stimulation

TaVNS-AEQ

This questionnaire is designed to capture adverse events during vagus nerve stimulation studies. It is only used for research purposes. Data is analyzed in a pseudonymized manner.

There is no right or wrong in the answers. In case of uncertainty, please provide the answer that applies best. Thank you very much for your help.

Question 1

Have your noticed stimulation symptoms during this study cycle? Please check the box for **"yes" or "no"**.

☐ Yes ☐ No ➡ Proceed to question 2.

⬇

Please list the symptoms you experienced and answer all related questions in the right columns in the same row for each symptom. Always mark only one box. Repeat this process for all symptoms.

List of symptoms	Severity level<br>**1, 2 or 3**<br>1= mild<br>2= moderate<br>3= severe<br>➡	Start time<br>**0, 1, 2 or 3**<br>0= beginning<br>1= middle<br>2= end<br>3= after study cycle<br>➡	After study cycle<br>**0 or 1**<br>0=symptom still present<br>1=symptom disappeare d<br>➡	Duration of symptom<br>**0, 1, 2, 3 or 4**<br>0= <10min<br>1=11-25min<br>2= 26-49min<br>3= 50min (beginning until end)<br>4= only during electrical stimulation<br>➡	Potential reason<br>**0,1, 2 or 3**<br>0= study setting or participation<br>1= stimulation itself<br>2= pre-existing medical condition, symptom known<br>3=randomly without apparent reason<br>➡	Symptom strong impairing during study?<br>**Yes or no**

	1 2 3<br>☐ ☐ ☐	0 1 2 3<br>☐ ☐ ☐ ☐	0 1<br>☐ ☐	0 1 2 3 4<br>☐ ☐ ☐ ☐ ☐	0 1 2 3<br>☐ ☐ ☐ ☐	Yes No<br>☐ ☐
	1 2 3<br>☐ ☐ ☐	0 1 2 3<br>☐ ☐ ☐ ☐	0 1<br>☐ ☐	0 1 2 3 4<br>☐ ☐ ☐ ☐ ☐	0 1 2 3<br>☐ ☐ ☐ ☐	Yes No<br>☐ ☐
	1 2 3<br>☐ ☐ ☐	0 1 2 3<br>☐ ☐ ☐ ☐	0 1<br>☐ ☐	0 1 2 3 4<br>☐ ☐ ☐ ☐ ☐	0 1 2 3<br>☐ ☐ ☐ ☐	Yes No<br>☐ ☐
	1 2 3<br>☐ ☐ ☐	0 1 2 3<br>☐ ☐ ☐ ☐	0 1<br>☐ ☐	0 1 2 3 4<br>☐ ☐ ☐ ☐ ☐	0 1 2 3<br>☐ ☐ ☐ ☐	Yes No<br>☐ ☐

<u>Question 2</u>

Please read the listed symptoms in the left column of the table on the next page of this questionnaire. **If you did not experience this symptom**, check the box for “no”. In case of “no”, the columns in the table to the right do not need to be filled out in that case. Read the symptom listed below and repeat this procedure.

If you experienced the symptom, check the box for “yes”. Please answer all further questions in the right columns in the same row of this symptom. **Always mark only one box**.

If you have any questions, feel free to ask the study coordinators.

List of symptoms	Symptom exper-ienced? **Yes or no**<br><br>If yes ➡	Severity level **1, 2 or 3**<br>1= mild<br>2= moderate<br>3= severe ➡	Start time **0, 1, 2 or 3**<br>0= beginning<br>1= middle<br>2= end<br>3= after study cycle ➡	After study cycle **0 or 1**<br>0=symptom still present<br>1=symptom disappeare d ➡	Duration of symptom **0, 1, 2, 3 or 4**<br>0= <10min<br>1=11-25min<br>2= 26-49min<br>3= 50min (beginning until end)<br>4= only during electrical stimulation ➡	Potential reason **0,1, 2 or 3**<br>0= study setting or participation<br>1= stimulation itself<br>2= pre-existing medical condition, symptom known<br>3=randomly without apparent reason ➡	Symptom strong impairing during study? **Yes or no**
Skin redness	No ☐ ⬇ Yes ☐ ➡	1 ☐ 2 ☐ 3 ☐	0 ☐ 1 ☐ 2 ☐ 3 ☐	0 ☐ 1 ☐	0 ☐ 1 ☐ 2 ☐ 3 ☐ 4 ☐	0 ☐ 1 ☐ 2 ☐ 3 ☐	Yes ☐ No ☐
Stinging sensation	No ☐ ⬇ Yes ☐ ➡	1 ☐ 2 ☐ 3 ☐	0 ☐ 1 ☐ 2 ☐ 3 ☐	0 ☐ 1 ☐	0 ☐ 1 ☐ 2 ☐ 3 ☐ 4 ☐	0 ☐ 1 ☐ 2 ☐ 3 ☐	Yes ☐ No ☐
Tingling sensation	No ☐ ⬇ Yes ☐ ➡	1 ☐ 2 ☐ 3 ☐	0 ☐ 1 ☐ 2 ☐ 3 ☐	0 ☐ 1 ☐	0 ☐ 1 ☐ 2 ☐ 3 ☐ 4 ☐	0 ☐ 1 ☐ 2 ☐ 3 ☐	Yes ☐ No ☐
Burning of the skin	No ☐ ⬇ Yes ☐ ➡	1 ☐ 2 ☐ 3 ☐	0 ☐ 1 ☐ 2 ☐ 3 ☐	0 ☐ 1 ☐	0 ☐ 1 ☐ 2 ☐ 3 ☐ 4 ☐	0 ☐ 1 ☐ 2 ☐ 3 ☐	Yes ☐ No ☐
Itching	No ☐ ⬇ Yes ☐ ➡	1 ☐ 2 ☐ 3 ☐	0 ☐ 1 ☐ 2 ☐ 3 ☐	0 ☐ 1 ☐	0 ☐ 1 ☐ 2 ☐ 3 ☐ 4 ☐	0 ☐ 1 ☐ 2 ☐ 3 ☐	Yes ☐ No ☐
Skin pressure sensation	No ☐ ⬇ Yes ☐ ➡	1 ☐ 2 ☐ 3 ☐	0 ☐ 1 ☐ 2 ☐ 3 ☐	0 ☐ 1 ☐	0 ☐ 1 ☐ 2 ☐ 3 ☐ 4 ☐	0 ☐ 1 ☐ 2 ☐ 3 ☐	Yes ☐ No ☐

List of symptoms	Symptom exper-ienced? **Yes or no**<br><br>If yes ➡	Severity level **1, 2 or 3**<br>1= mild<br>2= moderate<br>3= severe ➡	Start time **0, 1, 2 or 3**<br>0= beginning<br>1= middle<br>2= end<br>3= after study cycle ➡	After study cycle **0 or 1**<br>0=symptom still present<br>1=symptom disappeare d ➡	Duration of symptom **0, 1, 2, 3 or 4**<br>0= <10min<br>1=11-25min<br>2= 26-49min<br>3= 50min (beginning until end)<br>4= only during electrical stimulation ➡	Potential reason **0,1, 2 or 3**<br>0= study setting or participation<br>1= stimulation itself<br>2= pre-existing medical condition, symptom known<br>3=randomly without apparent reason ➡	Symptom strong impairing during study? **Yes or no**
Unpleasant skin sensation	No ☐ ⬇ Yes ☐ ➡	1 ☐ 2 ☐ 3 ☐	0 ☐ 1 ☐ 2 ☐ 3 ☐	0 ☐ 1 ☐	0 ☐ 1 ☐ 2 ☐ 3 ☐ 4 ☐	0 ☐ 1 ☐ 2 ☐ 3 ☐	Yes ☐ No ☐
Skin pain	No ☐ ⬇ Yes ☐ ➡	1 ☐ 2 ☐ 3 ☐	0 ☐ 1 ☐ 2 ☐ 3 ☐	0 ☐ 1 ☐	0 ☐ 1 ☐ 2 ☐ 3 ☐ 4 ☐	0 ☐ 1 ☐ 2 ☐ 3 ☐	Yes ☐ No ☐
Ear discomfort (e.g. fullness, pressure)	No ☐ ⬇ Yes ☐ ➡	1 ☐ 2 ☐ 3 ☐	0 ☐ 1 ☐ 2 ☐ 3 ☐	0 ☐ 1 ☐	0 ☐ 1 ☐ 2 ☐ 3 ☐ 4 ☐	0 ☐ 1 ☐ 2 ☐ 3 ☐	Yes ☐ No ☐
Tinnitus / ear noise	No ☐ ⬇ Yes ☐ ➡	1 ☐ 2 ☐ 3 ☐	0 ☐ 1 ☐ 2 ☐ 3 ☐	0 ☐ 1 ☐	0 ☐ 1 ☐ 2 ☐ 3 ☐ 4 ☐	0 ☐ 1 ☐ 2 ☐ 3 ☐	Yes ☐ No ☐
Neck pain	No ☐ ⬇ Yes ☐ ➡	1 ☐ 2 ☐ 3 ☐	0 ☐ 1 ☐ 2 ☐ 3 ☐	0 ☐ 1 ☐	0 ☐ 1 ☐ 2 ☐ 3 ☐ 4 ☐	0 ☐ 1 ☐ 2 ☐ 3 ☐	Yes ☐ No ☐
Facial and / or neck muscle cramps	No ☐ ⬇ Yes ☐ ➡	1 ☐ 2 ☐ 3 ☐	0 ☐ 1 ☐ 2 ☐ 3 ☐	0 ☐ 1 ☐	0 ☐ 1 ☐ 2 ☐ 3 ☐ 4 ☐	0 ☐ 1 ☐ 2 ☐ 3 ☐	Yes ☐ No ☐
Dizziness	No ☐ ⬇ Yes ☐ ➡	1 ☐ 2 ☐ 3 ☐	0 ☐ 1 ☐ 2 ☐ 3 ☐	0 ☐ 1 ☐	0 ☐ 1 ☐ 2 ☐ 3 ☐ 4 ☐	0 ☐ 1 ☐ 2 ☐ 3 ☐	Yes ☐ No ☐

List of symptoms	Symptom exper-ienced? **Yes or no** If yes ➡	Severity level **1, 2 or 3** 1= mild 2= moderate 3= severe ➡	Start time **0, 1, 2 or 3** 0= beginning 1= middle 2= end 3= after study cycle ➡	After study cycle **0 or 1** 0=symptom still present 1=symptom disappeared ➡	Duration of symptom **0, 1, 2, 3 or 4** 0= <10min 1=11-25min 2= 26-49min 3= 50min (beginning until end) 4= only during electrical stimulation ➡	Potential reason **0,1, 2 or 3** 0= study setting or participation 1= stimulation itself 2= pre-existing medical condition, symptom known 3=randomly without apparent reason ➡	Symptom strong impairing during study? **Yes or no**
Headaches	No ☐ ⬇ Yes ☐ ➡	1 ☐ 2 ☐ 3 ☐	0 ☐ 1 ☐ 2 ☐ 3 ☐	0 ☐ 1 ☐	0 ☐ 1 ☐ 2 ☐ 3 ☐ 4 ☐	0 ☐ 1 ☐ 2 ☐ 3 ☐	Yes ☐ No ☐
Changes in concentration	No ☐ ⬇ Yes ☐ ➡	1 ☐ 2 ☐ 3 ☐	0 ☐ 1 ☐ 2 ☐ 3 ☐	0 ☐ 1 ☐	0 ☐ 1 ☐ 2 ☐ 3 ☐ 4 ☐	0 ☐ 1 ☐ 2 ☐ 3 ☐	Yes ☐ No ☐
Fluctuation of feelings	No ☐ ⬇ Yes ☐ ➡	1 ☐ 2 ☐ 3 ☐	0 ☐ 1 ☐ 2 ☐ 3 ☐	0 ☐ 1 ☐	0 ☐ 1 ☐ 2 ☐ 3 ☐ 4 ☐	0 ☐ 1 ☐ 2 ☐ 3 ☐	Yes ☐ No ☐
Fatigue	No ☐ ⬇ Yes ☐ ➡	1 ☐ 2 ☐ 3 ☐	0 ☐ 1 ☐ 2 ☐ 3 ☐	0 ☐ 1 ☐	0 ☐ 1 ☐ 2 ☐ 3 ☐ 4 ☐	0 ☐ 1 ☐ 2 ☐ 3 ☐	Yes ☐ No ☐
Arousal	No ☐ ⬇ Yes ☐ ➡	1 ☐ 2 ☐ 3 ☐	0 ☐ 1 ☐ 2 ☐ 3 ☐	0 ☐ 1 ☐	0 ☐ 1 ☐ 2 ☐ 3 ☐ 4 ☐	0 ☐ 1 ☐ 2 ☐ 3 ☐	Yes ☐ No ☐
Malaise (unspecific)	No ☐ ⬇ Yes ☐ ➡	1 ☐ 2 ☐ 3 ☐	0 ☐ 1 ☐ 2 ☐ 3 ☐	0 ☐ 1 ☐	0 ☐ 1 ☐ 2 ☐ 3 ☐ 4 ☐	0 ☐ 1 ☐ 2 ☐ 3 ☐	Yes ☐ No ☐
Chest pain	No ☐ ⬇ Yes ☐ ➡	1 ☐ 2 ☐ 3 ☐	0 ☐ 1 ☐ 2 ☐ 3 ☐	0 ☐ 1 ☐	0 ☐ 1 ☐ 2 ☐ 3 ☐ 4 ☐	0 ☐ 1 ☐ 2 ☐ 3 ☐	Yes ☐ No ☐

List of symptoms	Symptom exper-ienced? **Yes or no**<br><br>If yes ➡	Severity level **1, 2 or 3**<br>1= mild<br>2= moderate<br>3= severe ➡	Start time **0, 1, 2 or 3**<br>0= beginning<br>1= middle<br>2= end<br>3= after study cycle ➡	After study cycle **0 or 1**<br>0=symptom still present<br>1=symptom disappeared ➡	Duration of symptom **0, 1, 2, 3 or 4**<br>0= <10min<br>1=11-25min<br>2= 26-49min<br>3= 50min (beginning until end)<br>4= only during electrical stimulation ➡	Potential reason **0,1, 2 or 3**<br>0= study setting or participation<br>1= stimulation itself<br>2= pre-existing medical condition, symptom known<br>3=randomly without apparent reason ➡	Symptom strong impairing during study? **Yes or no**
Cardiovascular symptoms (e.g. collapse, palpitation, weakness)	No ☐ ⬇ Yes ☐ ➡	1 ☐ 2 ☐ 3 ☐	0 ☐ 1 ☐ 2 ☐ 3 ☐	0 ☐ 1 ☐	0 ☐ 1 ☐ 2 ☐ 3 ☐ 4 ☐	0 ☐ 1 ☐ 2 ☐ 3 ☐	Yes ☐ No ☐
Nausea	No ☐ ⬇ Yes ☐ ➡	1 ☐ 2 ☐ 3 ☐	0 ☐ 1 ☐ 2 ☐ 3 ☐	0 ☐ 1 ☐	0 ☐ 1 ☐ 2 ☐ 3 ☐ 4 ☐	0 ☐ 1 ☐ 2 ☐ 3 ☐	Yes ☐ No ☐
Stomach pain	No ☐ ⬇ Yes ☐ ➡	1 ☐ 2 ☐ 3 ☐	0 ☐ 1 ☐ 2 ☐ 3 ☐	0 ☐ 1 ☐	0 ☐ 1 ☐ 2 ☐ 3 ☐ 4 ☐	0 ☐ 1 ☐ 2 ☐ 3 ☐	Yes ☐ No ☐
Constipation	No ☐ ⬇ Yes ☐ ➡	1 ☐ 2 ☐ 3 ☐	0 ☐ 1 ☐ 2 ☐ 3 ☐	0 ☐ 1 ☐	0 ☐ 1 ☐ 2 ☐ 3 ☐ 4 ☐	0 ☐ 1 ☐ 2 ☐ 3 ☐	Yes ☐ No ☐
Insomnia	No ☐ ⬇ Yes ☐ ➡	1 ☐ 2 ☐ 3 ☐	0 ☐ 1 ☐ 2 ☐ 3 ☐	0 ☐ 1 ☐	0 ☐ 1 ☐ 2 ☐ 3 ☐ 4 ☐	0 ☐ 1 ☐ 2 ☐ 3 ☐	Yes ☐ No ☐

Question 3

Would you be willing to repeat the stimulation as part of a study? Please check the box for **“yes” or “no“**.

Yes ☐ No ☐

If you marked “no“, why would you not take part in the study again? (please state reason)

Question 4

In this study, real vagus nerve stimulations as well as placebo (sham) stimulations are conducted.

Please assess whether it was a **real vagus or sham** stimulation in this study cycle. Please check one of the boxes.

Real vagus nerve stimulation	Placebo / sham stimulation
☐	☐

How confident are you in your decision? Please check only one box.

☐ **1** = Not confident at all
☐ **2** = Slightly confident
☐ **3** = Moderately confident
☐ **4** = Very confident
☐ **5** = Completely confident

Additional comments:

Thank you for completing the questionnaire!

Questionnaire for study coordinator

While the participant completes the participant version of the questionnaire, please fill out this questionnaire for study coordinators on the basis of observations during the study.

Question 1:

Has the participant questionnaire been fully and accurately filled out?

☐ Yes ☐ No

If you marked “no“, request the participant to fill it out completely.

Question 2:

Please mark the row of the study cycle that has been conducted with a cross.

	Study cycle
	**Placebo** stimulation with **30 seconds** electrical stimulation
	**Placebo** stimulation with **3.4 seconds** electrical stimulation
	**Therapeutic** stimulation with **30 seconds** electrical stimulation
	**Therapeutic** stimulation with **3.4 seconds** electrical stimulation

Question 3:

Which stimulation intensity has been administered? ________ mA

Has the intensity been changed during this study cycle?

☐ Yes ☐ No ➡ proceed to question 4

⬇

New stimulation intensity ________ mA

Change of intensity after _________ min

Reason for change: ____

Question 4:

Have symptoms / adverse events been noticed during or after stimulation?

____

____

____

Question 5:

Did the symptoms threaten the participant's health? Please check the box for **"yes" or "no"**.

☐ No ☐ Yes

If you marked "yes", which symptoms and why?

____

____

____

Question 6:

Did it come to an interruption of stimulation due to adverse events? Please check the box for **“yes“ or “no“.**

☐ Yes ☐ No

If your selected “yes“, please state the reason.

___

___

___

Question 7:

Which pre-existing medical conditions are known?

___

Which medications are taken regularly?

___

Thank you for completing the questionnaire!

Guidelines:

- Refer to our own recommendations for questionnaire analysis.
- Refer to checklist with recommendations from Farmer et al. 2020

9.2: TaVNS-AEQ – German version 3.0

Fragebogen zur transkutanen aurikulären Vagusnervstimulation

TaVNS-AEQ

Dieser Fragebogen dient dazu unerwünschte Ereignisse während der Studiendurchführung zu erfassen. Der Fragebogen wird nur für Forschungszwecke verwendet. Ihre Daten werden pseudoanonym ausgewertet.

Bei den Antworten gibt es kein „Richtig oder Falsch“. Bei Unsicherheiten bitten wir Sie eine Antwort anzugeben, die am meisten auf Sie zutrifft. Vielen Dank für Ihre Mithilfe.

Frage 1

Haben Sie Symptome der Stimulation während der Studiendurchführung bemerkt? Bitte „ja“ **oder** „nein“ ankreuzen.

☐ Ja ☐ Nein ➡ Gehen Sie weiter zu Frage 2.

⬇

Bitte listen Sie die erlebten Symptome in der linken Spalte auf und beantworte Sie die dazugehörigen Fragen in den rechten Spalten der gleichen Zeile für jedes Symptom. Bitte jeweils nur ein Kästchen ankreuzen. Wiederholen Sie den Prozess für alle erlebten Symptome.

Liste der Symptome	Schweregrad **1, 2 oder 3** 1= mild 2= mittel / moderat 3= schwer ➡	Startzeitpunkt **0, 1, 2 oder 3** 0= Beginn 1= Mitte 2= Ende 3= nach dem Studiendurchlauf ➡	Nach Ende Studien-durchlauf **0 oder 1** 0=Symptom weiterhin vorhanden 1=Symptom weg ➡	Gesamtdauer des Symptoms **0, 1, 2, 3 oder 4** 0= unter 10min 1=11-25min 2=26-49min 3=50min (Anfang bis Ende) 4=nur bei elektrischer Stimulation ➡	Vermutlicher Grund **0,1, 2 oder 3** 0= Studiensetting/-teilnahme 1= die Stimulation 2= Vorerkrankung / Symptom bekannt 3=zufällig ohne erkennbaren Grund ➡	Symptom stark beeinträch -tigend während Studien? **Ja oder nein**

	1 2 3<br>☐ ☐ ☐	0 1 2 3<br>☐ ☐ ☐ ☐	0 1<br>☐ ☐	0 1 2 3 4<br>☐ ☐ ☐ ☐ ☐	0 1 2 3<br>☐ ☐ ☐ ☐	Ja Nein<br>☐ ☐
	1 2 3<br>☐ ☐ ☐	0 1 2 3<br>☐ ☐ ☐ ☐	0 1<br>☐ ☐	0 1 2 3 4<br>☐ ☐ ☐ ☐ ☐	0 1 2 3<br>☐ ☐ ☐ ☐	Ja Nein<br>☐ ☐
	1 2 3<br>☐ ☐ ☐	0 1 2 3<br>☐ ☐ ☐ ☐	0 1<br>☐ ☐	0 1 2 3 4<br>☐ ☐ ☐ ☐ ☐	0 1 2 3<br>☐ ☐ ☐ ☐	Ja Nein<br>☐ ☐
	1 2 3<br>☐ ☐ ☐	0 1 2 3<br>☐ ☐ ☐ ☐	0 1<br>☐ ☐	0 1 2 3 4<br>☐ ☐ ☐ ☐ ☐	0 1 2 3<br>☐ ☐ ☐ ☐	Ja Nein<br>☐ ☐

<u>Frage 2</u>

Bitte lesen Sie die aufgelisteten Symptome in der linken Spalte der Tabelle auf der nächsten Seite dieses Fragebogens. **Wenn das Symptom bei Ihnen nicht aufgetreten ist,** setzen Sie ein Kreuz in das Kästchen unter dem Wort „nein“. Die Spalten der Tabelle rechts daneben müssen dann nicht ausgefüllt werden. Lesen Sie die weiteren aufgelisteten Symptome und wiederholen Sie den Vorgang.

Wenn das Symptom aufgetreten ist, kreuzen Sie zuerst das Kästchen unter dem Wort „ja“ an. Beantworten sie alle weiteren, in der Zeile des Symptoms stehenden Fragen. Kreuzen Sie bei jeder Frage **genau ein Kästchen** an.

Bei Fragen wenden Sie sich gerne an die Testleiter.

Liste der Symptome	Symptom vorhanden? **Ja oder nein** Wenn ja ➡	Schweregrad **1, 2 oder 3** 1= mild 2= mittel / moderat 3= schwer ➡	Startzeitpunkt **0, 1, 2 oder 3** 0= Beginn 1= Mitte 2= Ende 3= nach dem Studiendurchlauf ➡	Nach Ende Studien-durchlauf **0 oder 1** 0=Symptom weiterhin vorhanden 1=Symptom weg ➡	Gesamtdauer des Symptoms **0, 1, 2, 3 oder 4** 0= unter 10min 1=11-25min 2=26-49min 3=50min (Anfang bis Ende) 4=nur bei elektrischer Stimulation ➡	Vermutlicher Grund **0,1, 2 oder 3** 0= Studiensetting/-teilnahme 1= die Stimulation 2= Vorerkrankung / Symptom bekannt 3=zufällig ohne erkennbaren Grund ➡	Symptom stark beeinträch-tigend während Studien? **Ja oder nein**
Hautrötung	Nein ☐ ⬇ Ja ☐ ➡	1 ☐ 2 ☐ 3 ☐	0 ☐ 1 ☐ 2 ☐ 3 ☐	0 ☐ 1 ☐	0 ☐ 1 ☐ 2 ☐ 3 ☐ 4 ☐	0 ☐ 1 ☐ 2 ☐ 3 ☐	Ja ☐ Nein ☐
Stechendes Gefühl an der Haut	Nein ☐ ⬇ Ja ☐ ➡	1 ☐ 2 ☐ 3 ☐	0 ☐ 1 ☐ 2 ☐ 3 ☐	0 ☐ 1 ☐	0 ☐ 1 ☐ 2 ☐ 3 ☐ 4 ☐	0 ☐ 1 ☐ 2 ☐ 3 ☐	Ja ☐ Nein ☐
Kribbelgefühl an der Haut	Nein ☐ ⬇ Ja ☐ ➡	1 ☐ 2 ☐ 3 ☐	0 ☐ 1 ☐ 2 ☐ 3 ☐	0 ☐ 1 ☐	0 ☐ 1 ☐ 2 ☐ 3 ☐ 4 ☐	0 ☐ 1 ☐ 2 ☐ 3 ☐	Ja ☐ Nein ☐
Brennen der Haut	Nein ☐ ⬇ Ja ☐ ➡	1 ☐ 2 ☐ 3 ☐	0 ☐ 1 ☐ 2 ☐ 3 ☐	0 ☐ 1 ☐	0 ☐ 1 ☐ 2 ☐ 3 ☐ 4 ☐	0 ☐ 1 ☐ 2 ☐ 3 ☐	Ja ☐ Nein ☐
Juckreiz an der Haut	Nein ☐ ⬇ Ja ☐ ➡	1 ☐ 2 ☐ 3 ☐	0 ☐ 1 ☐ 2 ☐ 3 ☐	0 ☐ 1 ☐	0 ☐ 1 ☐ 2 ☐ 3 ☐ 4 ☐	0 ☐ 1 ☐ 2 ☐ 3 ☐	Ja ☐ Nein ☐
Druckgefühl an der Haut	Nein ☐ ⬇ Ja ☐ ➡	1 ☐ 2 ☐ 3 ☐	0 ☐ 1 ☐ 2 ☐ 3 ☐	0 ☐ 1 ☐	0 ☐ 1 ☐ 2 ☐ 3 ☐ 4 ☐	0 ☐ 1 ☐ 2 ☐ 3 ☐	Ja ☐ Nein ☐

Liste der Symptome	Symptom vorhanden? **Ja oder nein** Wenn ja ➡	Schweregrad **1, 2 oder 3** 1= mild 2= mittel / moderat 3= schwer ➡	Startzeitpunkt **0, 1, 2 oder 3** 0= Beginn 1= Mitte 2= Ende 3= nach dem Studiendurchlauf ➡	Nach Ende Studien-durchlauf **0 oder 1** 0=Symptom weiterhin vorhanden 1=Symptom weg ➡	Gesamtdauer des Symptoms **0, 1, 2, 3 oder 4** 0= unter 10min 1=11-25min 2=26-49min 3=50min (Anfang bis Ende) 4=nur bei elektrischer Stimulation ➡	Vermutlicher Grund **0,1, 2 oder 3** 0= Studiensetting/-teilnahme 1= die Stimulation 2= Vorerkrankung / Symptom bekannt 3=zufällig ohne erkennbaren Grund ➡	Symptom stark beeinträch-tigend während Studien? **Ja oder nein**
Weiteres/anders unangenehmes Hautgefühl	Nein ☐ ⬇ Ja ☐ ➡	1 ☐ 2 ☐ 3 ☐	0 ☐ 1 ☐ 2 ☐ 3 ☐	0 ☐ 1 ☐	0 ☐ 1 ☐ 2 ☐ 3 ☐ 4 ☐	0 ☐ 1 ☐ 2 ☐ 3 ☐	Ja ☐ Nein ☐
Schmerzen an der Haut	Nein ☐ ⬇ Ja ☐ ➡	1 ☐ 2 ☐ 3 ☐	0 ☐ 1 ☐ 2 ☐ 3 ☐	0 ☐ 1 ☐	0 ☐ 1 ☐ 2 ☐ 3 ☐ 4 ☐	0 ☐ 1 ☐ 2 ☐ 3 ☐	Ja ☐ Nein ☐
Ohrbeschwerden (z.B. Völlegefühl, Druck)	Nein ☐ ⬇ Ja ☐ ➡	1 ☐ 2 ☐ 3 ☐	0 ☐ 1 ☐ 2 ☐ 3 ☐	0 ☐ 1 ☐	0 ☐ 1 ☐ 2 ☐ 3 ☐ 4 ☐	0 ☐ 1 ☐ 2 ☐ 3 ☐	Ja ☐ Nein ☐
Tinnitus / Ohrgeräusch	Nein ☐ ⬇ Ja ☐ ➡	1 ☐ 2 ☐ 3 ☐	0 ☐ 1 ☐ 2 ☐ 3 ☐	0 ☐ 1 ☐	0 ☐ 1 ☐ 2 ☐ 3 ☐ 4 ☐	0 ☐ 1 ☐ 2 ☐ 3 ☐	Ja ☐ Nein ☐
Nackenschmerzen	Nein ☐ ⬇ Ja ☐ ➡	1 ☐ 2 ☐ 3 ☐	0 ☐ 1 ☐ 2 ☐ 3 ☐	0 ☐ 1 ☐	0 ☐ 1 ☐ 2 ☐ 3 ☐ 4 ☐	0 ☐ 1 ☐ 2 ☐ 3 ☐	Ja ☐ Nein ☐
Muskelkrämpfe im Gesicht und / oder Nacken	Nein ☐ ⬇ Ja ☐ ➡	1 ☐ 2 ☐ 3 ☐	0 ☐ 1 ☐ 2 ☐ 3 ☐	0 ☐ 1 ☐	0 ☐ 1 ☐ 2 ☐ 3 ☐ 4 ☐	0 ☐ 1 ☐ 2 ☐ 3 ☐	Ja ☐ Nein ☐
Schwindel	Nein ☐ ⬇ Ja ☐ ➡	1 ☐ 2 ☐ 3 ☐	0 ☐ 1 ☐ 2 ☐ 3 ☐	0 ☐ 1 ☐	0 ☐ 1 ☐ 2 ☐ 3 ☐ 4 ☐	0 ☐ 1 ☐ 2 ☐ 3 ☑	Ja ☐ Nein ☐

Liste der Symptome	Symptom vorhanden?<br>**Ja oder nein**<br>Wenn ja ➡	Schweregrad<br>**1, 2 oder 3**<br>1= mild<br>2= mittel / moderat<br>3= schwer ➡	Startzeitpunkt<br>**0, 1, 2 oder 3**<br>0= Beginn<br>1= Mitte<br>2= Ende<br>3= nach dem Studiendurchlauf ➡	Nach Ende Studien-durchlauf<br>**0 oder 1**<br>0=Symptom weiterhin vorhanden<br>1=Symptom weg ➡	Gesamtdauer des Symptoms<br>**0, 1, 2, 3 oder 4**<br>0= unter 10min<br>1=11-25min<br>2=26-49min<br>3=50min (Anfang bis Ende)<br>4=nur bei elektrischer Stimulation ➡	Vermutlicher Grund<br>**0,1, 2 oder 3**<br>0= Studiensetting/-teilnahme<br>1= die Stimulation<br>2= Vorerkrankung / Symptom bekannt<br>3=zufällig ohne erkennbaren Grund ➡	Symptom stark beeinträch-tigend während Studien?<br>**Ja oder nein**
Kopfschmerzen	Nein ☐ ⬇ Ja ☐ ➡	1 ☐ 2 ☐ 3 ☐	0 ☐ 1 ☐ 2 ☐ 3 ☐	0 ☐ 1 ☐	0 ☐ 1 ☐ 2 ☐ 3 ☐ 4 ☐	0 ☐ 1 ☐ 2 ☐ 3 ☐	Ja ☐ Nein ☐
Konzentrations-veränderungen	Nein ☐ ⬇ Ja ☐ ➡	1 ☐ 2 ☐ 3 ☐	0 ☐ 1 ☐ 2 ☐ 3 ☐	0 ☐ 1 ☐	0 ☐ 1 ☐ 2 ☐ 3 ☐ 4 ☐	0 ☐ 1 ☐ 2 ☐ 3 ☐	Ja ☐ Nein ☐
Gefühls-veränderungen	Nein ☐ ⬇ Ja ☐ ➡	1 ☐ 2 ☐ 3 ☐	0 ☐ 1 ☐ 2 ☐ 3 ☐	0 ☐ 1 ☐	0 ☐ 1 ☐ 2 ☐ 3 ☐ 4 ☐	0 ☐ 1 ☐ 2 ☐ 3 ☐	Ja ☐ Nein ☐
Unwohlsein (unspezifisch)	Nein ☐ ⬇ Ja ☐ ➡	1 ☐ 2 ☐ 3 ☐	0 ☐ 1 ☐ 2 ☐ 3 ☐	0 ☐ 1 ☐	0 ☐ 1 ☐ 2 ☐ 3 ☐ 4 ☐	0 ☐ 1 ☐ 2 ☐ 3 ☐	Ja ☐ Nein ☐
Aufregung / Erregung	Nein ☐ ⬇ Ja ☐ ➡	1 ☐ 2 ☐ 3 ☐	0 ☐ 1 ☐ 2 ☐ 3 ☐	0 ☐ 1 ☐	0 ☐ 1 ☐ 2 ☐ 3 ☐ 4 ☐	0 ☐ 1 ☐ 2 ☐ 3 ☐	Ja ☐ Nein ☐
Müdigkeit / Fatigue	Nein ☐ ⬇ Ja ☐ ➡	1 ☐ 2 ☐ 3 ☐	0 ☐ 1 ☐ 2 ☐ 3 ☐	0 ☐ 1 ☐	0 ☐ 1 ☐ 2 ☐ 3 ☐ 4 ☐	0 ☐ 1 ☐ 2 ☐ 3 ☐	Ja ☐ Nein ☐
Brustschmerzen	Nein ☐ ⬇ Ja ☐ ➡	1 ☐ 2 ☐ 3 ☐	0 ☐ 1 ☐ 2 ☐ 3 ☐	0 ☐ 1 ☐	0 ☐ 1 ☐ 2 ☐ 3 ☐ 4 ☐	0 ☐ 1 ☐ 2 ☐ 3 ☐	Ja ☐ Nein ☐

Liste der Symptome	Symptom vorhanden?<br>**Ja oder nein**<br>Wenn ja ➡	Schweregrad<br>**1, 2 oder 3**<br>1= mild<br>2= mittel / moderat<br>3= schwer ➡	Startzeitpunkt<br>**0, 1, 2 oder 3**<br>0= Beginn<br>1= Mitte<br>2= Ende<br>3= nach dem Studiendurchlauf ➡	Nach Ende Studien-durchlauf<br>**0 oder 1**<br>0=Symptom weiterhin vorhanden<br>1=Symptom weg ➡	Gesamtdauer des Symptoms<br>**0, 1, 2, 3 oder 4**<br>0= unter 10min<br>1=11-25min<br>2=26-49min<br>3=50min (Anfang bis Ende)<br>4=nur bei elektrischer Stimulation ➡	Vermutlicher Grund<br>**0,1, 2 oder 3**<br>0= Studiensetting/-teilnahme<br>1= die Stimulation<br>2= Vorerkrankung / Symptom bekannt<br>3=zufällig ohne erkennbaren Grund ➡	Symptom stark beeinträch-tigend während Studien?<br>**Ja oder nein**
Kardiovaskuläre Symptome (z.B. Kollaps, Palpitationen, Schwäche)	Nein ☐ ⬇ Ja ☐ ➡	1 ☐ 2 ☐ 3 ☐	0 ☐ 1 ☐ 2 ☐ 3 ☐	0 ☐ 1 ☐	0 ☐ 1 ☐ 2 ☐ 3 ☐ 4 ☐	0 ☐ 1 ☐ 2 ☐ 3 ☐	Ja ☐ Nein ☐
Übelkeit	Nein ☐ ⬇ Ja ☐ ➡	1 ☐ 2 ☐ 3 ☐	0 ☐ 1 ☐ 2 ☐ 3 ☐	0 ☐ 1 ☐	0 ☐ 1 ☐ 2 ☐ 3 ☐ 4 ☐	0 ☐ 1 ☐ 2 ☐ 3 ☐	Ja ☐ Nein ☐
Bauchschmerzen	Nein ☐ ⬇ Ja ☐ ➡	1 ☐ 2 ☐ 3 ☐	0 ☐ 1 ☐ 2 ☐ 3 ☐	0 ☐ 1 ☐	0 ☐ 1 ☐ 2 ☐ 3 ☐ 4 ☐	0 ☐ 1 ☐ 2 ☐ 3 ☐	Ja ☐ Nein ☐
Obstipation	Nein ☐ ⬇ Ja ☐ ➡	1 ☐ 2 ☐ 3 ☐	0 ☐ 1 ☐ 2 ☐ 3 ☐	0 ☐ 1 ☐	0 ☐ 1 ☐ 2 ☐ 3 ☐ 4 ☐	0 ☐ 1 ☐ 2 ☐ 3 ☐	Ja ☐ Nein ☐
Schlaflosigkeit / -störungen	Nein ☐ ⬇ Ja ☐ ➡	1 ☐ 2 ☐ 3 ☐	0 ☐ 1 ☐ 2 ☐ 3 ☐	0 ☐ 1 ☐	0 ☐ 1 ☐ 2 ☐ 3 ☐ 4 ☐	0 ☐ 1 ☐ 2 ☐ 3 ☐	Ja ☐ Nein ☐

Frage 3

Würden Sie eine weitere Vagusnervenstimulation im Kontext von einer Studienteilnahme durchführen lassen? Bitte kreuzen Sie entweder „ja“ **oder** „nein“ an.

Ja Nein

☐ ☐

Wenn Sie „nein“ angekreuzt haben, warum würden Sie keine weitere Stimulation durchführen lassen? (Grund bitte anführen)

____

Frage 4

Im Rahmen dieser Studie werden therapeutische, also wahre Stimulationen des Vagusnervs und auch Placebostimulationen (Scheinstimulationen) durchgeführt. Schätzen Sie bitte ein, ob es sich bei diesen Studiendurchgang vermutlich um eine therapeutische Vagusnervstimulation **oder** um eine Placebostimulation gehandelt hat. Kreuzen Sie **eines** der beiden Möglichkeiten an.

Wahre Stimulation Placebo

☐ ☐

Wie sicher sind Sie sich bei Ihrer Entscheidung? Bitte kreuzen Sie nur ein Kästchen an.

☐ **1** = Überhaupt nicht sicher
☐ **2** = Leicht sicher
☐ **3** = Mäßig sicher
☐ **4** = Sehr sicher
☐ **5** = Vollkommen sicher

Platz für weitere Bemerkungen:

Vielen Dank für die Beantwortung des Fragebogens!

Fragebogen für Testleiter

Während der Proband den Probandenfragebogen ausfüllt, bitte diesen Testleiterfragebogen auf Grundlage der Beobachtungen während der Studie ausfüllen.

Frage 1:

Ist der Fragebogen vollständig und korrekt ausgefüllt worden?

☐ Ja ☐ Nein

Wenn „nein" angekreuzt wurde, den Probanden darum bitten, diesen vollständig auszufüllen.

Frage 2:

Bitte geben Sie an, welche der Studienblöcke durchgeführt worden ist. Setzen Sie dafür ein Kreuz in die richtige Spalte in der Tabelle.

	Studiendurchgang
	**Placebo** Stimulation mit **30 Sekunden** Stromverabreichung
	**Placebo** Stimulation mit **3,4 Sekunden** Stromverabreichung
	**Therapeutische** Stimulation mit **30 Sekunden** Stromverabreichung
	**Therapeutische** Stimulation mit **3,4 Sekunden** Stromverabreichung

Frage 3:

Mit welcher Intensität wurde in diesem Studiendurchgang stimuliert? ________ mA

Wurde die Intensität während des Durchgangs verändert?

☐ Ja ☐ Nein ➡ weiter zu Frage 4

⬇

Neue Intensität ________ mA

Veränderung der Intensität nach _________ min

Warum verändert: ____

Frage 4:

Sind Symptome / unerwünschte Ereignisse während der Stimulation aufgefallen?

____

____

____

Frage 5:

Kam es zu Symptomen, die den Gesundheitszustand des Probanden ernstzunehmend gefährdet haben? Bitte „ja“ **oder** „nein“ ankreuzen.

☐ Nein ☐ Ja

Wenn „ja“, welche und warum?

____

____

____

<u>Frage 6:</u>

Kam es zum Abbruch der Stimulation aufgrund von unerwünschten Ereignissen? Bitte „ja“ **oder** „nein“ ankreuzen.

☐ Ja ☐ Nein

Wenn „ja“, warum?

____

____

____

<u>Frage 7:</u>

Welche Vorerkrankungen sind bekannt?

____

Welche Medikamente werden regelmäßig eingenommen?

____

Danke für das Ausfüllen des Fragebogens!

Hinweise:

- Siehe eigene Empfehlungen zur Auswertung des Fragebogens
- Siehe Checkliste für Empfehlungen zur Berichterstattung von Farmer et al. 2020

9.3 TaVNS-AEQ – Italian version 3.0

Questionario sulla stimolazione transcutanea del nervo vago auricolare

Questo questionario viene utilizzato per registrare gli eventi indesiderati durante il corso dello studio. Il questionario sarà utilizzato solo a scopo di ricerca. I dati saranno analizzati in forma pseudo-anonima.

Non esiste una risposta "giusta o sbagliata". Se non è sicuro, la preghiamo di indicare la risposta che più le si addice.

Molte grazie per la collaborazione.

Domanda 1

Ha avvertito dei fastidi durante il corso dello studio? Per favore selezioni con una crocetta „si“ o „no“.

☐ Si ☐ No ➡ Procedere alla domanda 2.

⬇

Si prega di elencare i sintomi sperimentati nella colonna di sinistra e di rispondere alle domande corrispondenti nelle colonne di destra della stessa riga per ciascun sintomo. Si prega di selezionare una sola casella per ogni risposta. Ripetere il processo per tutti i sintomi sperimentati.

Lista dei sintomi	Gravità del fastidio<br>1, 2 o 3<br>1= lieve<br>2= moderato<br>3= grave ➡	Momento di inizio<br>0, 1, 2 o 3<br>0= Inizio<br>1= A metà<br>2= Alla fine<br>3= Termine del ciclo di studio ➡	Dopo la fine del ciclo di studio<br>0 o 1<br>0= sintomo persiste<br>1= sintomo non persiste ➡	Totale durata del fastidio<br>0, 1, 2, 3 o 4<br>0= meno di 10min<br>1=11-25min<br>2=26-49min<br>3=50min (dall'inizio alla fine)<br>4=solo durante la stimolazione elettrica ➡	Possibile motivo<br>0,1, 2 o 3<br>0= caratteristiche dello studio o la partecipazione ad esso<br>1= la stimolazione<br>2= Condizione medica o sintomo noto ➡<br>3=casuale, senza apparente motivo	Sintomo fortemente ostacolante durante gli studi?<br>Si o no

	1 ☐ 2 ☐ 3 ☐	0 ☐ 1 ☐ 2 ☐ 3 ☐	0 ☐ 1 ☐	0 ☐ 1 ☐ 2 ☐ 3 ☐ 4 ☐	0 ☐ 1 ☐ 2 ☐ 3 ☐	Si ☐ No ☐
	1 ☐ 2 ☐ 3 ☐	0 ☐ 1 ☐ 2 ☐ 3 ☐	0 ☐ 1 ☐	0 ☐ 1 ☐ 2 ☐ 3 ☐ 4 ☐	0 ☐ 1 ☐ 2 ☐ 3 ☐	Si ☐ No ☐
	1 ☐ 2 ☐ 3 ☐	0 ☐ 1 ☐ 2 ☐ 3 ☐	0 ☐ 1 ☐	0 ☐ 1 ☐ 2 ☐ 3 ☐ 4 ☐	0 ☐ 1 ☐ 2 ☐ 3 ☐	Si ☐ No ☐
	1 ☐ 2 ☐ 3 ☐	0 ☐ 1 ☐ 2 ☐ 3 ☐	0 ☐ 1 ☐	0 ☐ 1 ☐ 2 ☐ 3 ☐ 4 ☐	0 ☐ 1 ☐ 2 ☐ 3 ☐	Si ☐ No ☐

<u>Domanda 2</u>

La preghiamo di leggere i sintomi elencati nella colonna di sinistra della tabella della pagina successiva di questo questionario.

Se non ha riscontrato il sintomo, selezioni la casella sotto la parola "no". In questo caso le corrispondenti colonne della tabella a destra non devono essere compilate. Legga gli altri sintomi elencati e ripeta la procedura.

Se si ha riscontrato il sintomo, selezioni la casella sotto la parola "sì". Poi risponda a tutte le altre domande della riga corrispondente al sintomo riscontrato. Selezioni **esattamente una casella** per ogni domanda.

In caso di domande, contatti l'amministratore del test.

Lista dei sintomi	Sintomo riscontato ?<br>Sì o no<br>Se si ➡	Gravità del fastidio<br>1, 2 o 3<br>1= lieve<br>2= moderato<br>3= grave ➡	Momento di inizio<br>0, 1, 2 o 3<br>0= Inizio<br>1= A metà<br>2= Alla fine<br>3= Termine del ciclo di studio ➡	Dopo la fine del ciclo di studio<br>0 o 1<br>0= sintomo persiste<br>1= sintomo non persiste ➡	Totale durata del fastidio<br>0, 1, 2, 3 o 4<br>0= meno di 10min<br>1=11-25min<br>2=26-49min<br>3=50min (dall'inizio alla fine)<br>4=solo durante la stimolazione elettrica ➡	Possibile motivo<br>0,1, 2 o 3<br>0= caratteristiche dello studio o la partecipazione ad esso<br>1= la stimolazione<br>2= Condizione medica o sintomo noto ➡<br>3=casuale, senza apparente motivo	Sintomo fortemente ostacolante durante gli studi?<br>Si o no
Arrossamento della pelle	No ☐ ⬇ Si ☐ ➡	1 ☐ 2 ☐ 3 ☐	0 ☐ 1 ☐ 2 ☐ 3 ☐	0 ☐ 1 ☐	0 ☐ 1 ☐ 2 ☐ 3 ☐ 4 ☐	0 ☐ 1 ☐ 2 ☐ 3 ☐	Si ☐ No ☐
Sensazione pungente	No ☐ ⬇ Si ☐ ➡	1 ☐ 2 ☐ 3 ☐	0 ☐ 1 ☐ 2 ☐ 3 ☐	0 ☐ 1 ☐	0 ☐ 1 ☐ 2 ☐ 3 ☐ 4 ☐	0 ☐ 1 ☐ 2 ☐ 3 ☐	Si ☐ No ☐
Formicolio sulla pelle	No ☐ ⬇ Si ☐ ➡	1 ☐ 2 ☐ 3 ☐	0 ☐ 1 ☐ 2 ☐ 3 ☐	0 ☐ 1 ☐	0 ☐ 1 ☐ 2 ☐ 3 ☐ 4 ☐	0 ☐ 1 ☐ 2 ☐ 3 ☐	Si ☐ No ☐
Bruciore sulla pelle	No ☐ ⬇ Si ☐ ➡	1 ☐ 2 ☐ 3 ☐	0 ☐ 1 ☐ 2 ☐ 3 ☐	0 ☐ 1 ☐	0 ☐ 1 ☐ 2 ☐ 3 ☐ 4 ☐	0 ☐ 1 ☐ 2 ☐ 3 ☐	Si ☐ No ☐
Prurito sulla pelle	No ☐ ⬇ Si ☐ ➡	1 ☐ 2 ☐ 3 ☐	0 ☐ 1 ☐ 2 ☐ 3 ☐	0 ☐ 1 ☐	0 ☐ 1 ☐ 2 ☐ 3 ☐ 4 ☐	0 ☐ 1 ☐ 2 ☐ 3 ☐	Si ☐ No ☐
Sensazione di pressione sulla pelle	No ☐ ⬇ Si ☐ ➡	1 ☐ 2 ☐ 3 ☐	0 ☐ 1 ☐ 2 ☑ 3 ☐	0 ☐ 1 ☐	0 ☐ 1 ☐ 2 ☐ 3 ☐ 4 ☐	0 ☐ 1 ☐ 2 ☐ 3 ☐	Si ☐ No ☐

Lista dei sintomi	Sintomo riscontato ? Sì o no Se si ➡	Gravità del fastidio 1, 2 o 3 1= lieve 2= moderato 3= grave ➡	Momento di inizio 0, 1, 2 o 3 0= Inizio 1= A metà 2= Alla fine 3= Termine del ciclo di studio ➡	Dopo la fine del ciclo di studio 0 o 1 0= sintomo persiste 1= sintomo non persiste ➡	Totale durata del fastidio 0, 1, 2, 3 o 4 0= meno di 10min 1=11-25min 2=26-49min 3=50min (dall'inizio alla fine) 4=solo durante la stimolazione elettrica ➡	Possibile motivo 0,1, 2 o 3 0= caratteristiche dello studio o la partecipazione ad esso 1= la stimolazione 2= Condizione medica o sintomo noto ➡ 3=casuale, senza apparente motivo	Sintomo fortemente ostacolante durante gli studi? Si o no
Altra sensazione spiacevole sulla pelle	No ☐ ⬇ Si ☐ ➡	1 ☐ 2 ☐ 3 ☐	0 ☐ 1 ☐ 2 ☐ 3 ☐	0 ☐ 1 ☐	0 ☐ 1 ☐ 2 ☐ 3 ☐ 4 ☐	0 ☐ 1 ☐ 2 ☐ 3 ☐	Si ☐ No ☐
Dolore cutaneo	No ☐ ⬇ Si ☐ ➡	1 ☐ 2 ☐ 3 ☐	0 ☐ 1 ☐ 2 ☐ 3 ☐	0 ☐ 1 ☐	0 ☐ 1 ☐ 2 ☐ 3 ☐ 4 ☐	0 ☐ 1 ☐ 2 ☐ 3 ☐	Si ☐ No ☐
Fstidio all'orecchio (p.es. orecchie tappate, pressione)	No ☐ ⬇ Si ☐ ➡	1 ☐ 2 ☐ 3 ☐	0 ☐ 1 ☐ 2 ☐ 3 ☐	0 ☐ 1 ☐	0 ☐ 1 ☐ 2 ☐ 3 ☐ 4 ☐	0 ☐ 1 ☐ 2 ☐ 3 ☐	Si ☐ No ☐
Acufeni / ronzii nelle orecchie	No ☐ ⬇ Si ☐ ➡	1 ☐ 2 ☐ 3 ☐	0 ☐ 1 ☐ 2 ☐ 3 ☐	0 ☐ 1 ☐	0 ☐ 1 ☐ 2 ☐ 3 ☐ 4 ☐	0 ☐ 1 ☐ 2 ☐ 3 ☐	Si ☐ No ☐
Dolore al collo	No ☐ ⬇ Si ☐ ➡	1 ☐ 2 ☐ 3 ☐	0 ☐ 1 ☐ 2 ☐ 3 ☐	0 ☐ 1 ☐	0 ☐ 1 ☐ 2 ☐ 3 ☐ 4 ☐	0 ☐ 1 ☐ 2 ☐ 3 ☐	Si ☐ No ☐
Crampi muscolari al viso e/o al collo	No ☐ ⬇ Si ☐ ➡	1 ☐ 2 ☐ 3 ☐	0 ☐ 1 ☐ 2 ☐ 3 ☐	0 ☐ 1 ☐	0 ☐ 1 ☐ 2 ☐ 3 ☐ 4 ☐	0 ☐ 1 ☐ 2 ☐ 3 ☐	Si ☐ No ☐
Giramenti di testa	No ☐ ⬇ Si ☐ ➡	1 ☐ 2 ☐ 3 ☐	0 ☐ 1 ☐ 2 ☐ 3 ☐	0 ☐ 1 ☐	0 ☐ 1 ☐ 2 ☐ 3 ☐ 4 ☐	0 ☐ 1 ☐ 2 ☐ 3 ☐	Si ☐ No ☐

Lista dei sintomi	Sintomo riscontato ? Sì o no Se si ➡	Gravità del fastidio 1, 2 o 3 1= lieve 2= moderato 3= grave ➡	Momento di inizio 0, 1, 2 o 3 0= Inizio 1= A metà 2= Alla fine 3= Termine del ciclo di studio ➡	Dopo la fine del ciclo di studio 0 o 1 0= sintomo persiste 1= sintomo non persiste ➡	Totale durata del fastidio 0, 1, 2, 3 o 4 0= meno di 10min 1=11-25min 2=26-49min 3=50min (dall'inizio alla fine) 4=solo durante la stimolazione elettrica ➡	Possibile motivo 0,1, 2 o 3 0= caratteristiche dello studio o la partecipazione ad esso 1= la stimolazione 2= Condizione medica o sintomo noto ➡ 3=casuale, senza apparente motivo	Sintomo fortemente ostacolante durante gli studi? Si o no
Mal di testa	No ☐ ⬇ Si ☐ ➡	1 ☐ 2 ☐ 3 ☐	0 ☐ 1 ☐ 2 ☐ 3 ☐	0 ☐ 1 ☐	0 ☐ 1 ☐ 2 ☐ 3 ☐ 4 ☐	0 ☐ 1 ☐ 2 ☐ 3 ☐	Si ☐ No ☐
Alterazione della concentrazione	No ☐ ⬇ Si ☐ ➡	1 ☐ 2 ☐ 3 ☐	0 ☐ 1 ☐ 2 ☐ 3 ☐	0 ☐ 1 ☐	0 ☐ 1 ☐ 2 ☐ 3 ☐ 4 ☐	0 ☐ 1 ☐ 2 ☐ 3 ☐	Si ☐ No ☐
Sbalzi d'umore	No ☐ ⬇ Si ☐ ➡	1 ☐ 2 ☐ 3 ☐	0 ☐ 1 ☐ 2 ☐ 3 ☐	0 ☐ 1 ☐	0 ☐ 1 ☐ 2 ☐ 3 ☐ 4 ☐	0 ☐ 1 ☐ 2 ☐ 3 ☐	Si ☐ No ☐
Stanchezza	No ☐ ⬇ Si ☐ ➡	1 ☐ 2 ☐ 3 ☐	0 ☐ 1 ☐ 2 ☐ 3 ☐	0 ☐ 1 ☐	0 ☐ 1 ☐ 2 ☐ 3 ☐ 4 ☐	0 ☐ 1 ☐ 2 ☐ 3 ☐	Si ☐ No ☐
Agitazione	No ☐ ⬇ Si ☐ ➡	1 ☐ 2 ☐ 3 ☐	0 ☐ 1 ☐ 2 ☐ 3 ☐	0 ☐ 1 ☐	0 ☐ 1 ☐ 2 ☐ 3 ☐ 4 ☐	0 ☐ 1 ☐ 2 ☐ 3 ☐	Si ☐ No ☐
Disagio (non specifico)	No ☐ ⬇ Si ☐ ➡	1 ☐ 2 ☐ 3 ☐	0 ☐ 1 ☐ 2 ☐ 3 ☐	0 ☐ 1 ☐	0 ☐ 1 ☐ 2 ☐ 3 ☐ 4 ☐	0 ☐ 1 ☐ 2 ☐ 3 ☐	Si ☐ No ☐
Dolore al petto Insonnia	No ☐ ⬇ Si ☐ ➡	1 ☐ 2 ☐ 3 ☐	0 ☐ 1 ☐ 2 ☐ 3 ☐	0 ☐ 1 ☐	0 ☐ 1 ☐ 2 ☐ 3 ☐ 4 ☐	0 ☐ 1 ☐ 2 ☐ 3 ☐	Si ☐ No ☐

Lista dei sintomi	Sintomo riscontato ?<br>Sì o no<br>Se si ➡	Gravità del fastidio<br>1, 2 o 3<br>1= lieve<br>2= moderato<br>3= grave ➡	Momento di inizio<br>0, 1, 2 o 3<br>0= Inizio<br>1= A metà<br>2= Alla fine<br>3= Termine del ciclo di studio ➡	Dopo la fine del ciclo di studio<br>0 o 1<br>0= sintomo persiste<br>1= sintomo non persiste ➡	Totale durata del fastidio<br>0, 1, 2, 3 o 4<br>0= meno di 10min<br>1=11-25min<br>2=26-49min<br>3=50min (dall'inizio alla fine)<br>4=solo durante la stimolazione elettrica ➡	Possibile motivo<br>0,1, 2 o 3<br>0= caratteristiche dello studio o la partecipazione ad esso<br>1= la stimolazione<br>2= Condizione medica o sintomo noto ➡<br>3=casuale, senza apparente motivo	Sintomo fortemente ostacolante durante gli studi?<br>Si o no
Sintomi cardiovascolari<br>(p.es. palpitazioni, debolezza, svenimento o collasso)	No ☐ ⬇ Si ☐ ➡	1 ☐ 2 ☐ 3 ☐	0 ☐ 1 ☐ 2 ☐ 3 ☐	0 ☐ 1 ☐	0 ☐ 1 ☐ 2 ☐ 3 ☐ 4 ☐	0 ☐ 1 ☐ 2 ☐ 3 ☐	Si ☐ No ☐
Nausea	No ☐ ⬇ Si ☐ ➡	1 ☐ 2 ☐ 3 ☐	0 ☐ 1 ☐ 2 ☐ 3 ☐	0 ☐ 1 ☐	0 ☐ 1 ☐ 2 ☐ 3 ☐ 4 ☐	0 ☐ 1 ☐ 2 ☐ 3 ☐	Si ☐ No ☐
Dolore allo stomaco	No ☐ ⬇ Si ☐ ➡	1 ☐ 2 ☐ 3 ☐	0 ☐ 1 ☐ 2 ☐ 3 ☐	0 ☐ 1 ☐	0 ☐ 1 ☐ 2 ☐ 3 ☐ 4 ☐	0 ☐ 1 ☐ 2 ☐ 3 ☐	Si ☐ No ☐
Stitichezza	No ☐ ⬇ Si ☐ ➡	1 ☐ 2 ☐ 3 ☐	0 ☐ 1 ☐ 2 ☐ 3 ☐	0 ☐ 1 ☐	0 ☐ 1 ☐ 2 ☐ 3 ☐ 4 ☐	0 ☐ 1 ☐ 2 ☐ 3 ☐	Si ☐ No ☐
Insonnia	No ☐ ⬇ Si ☐ ➡	1 ☐ 2 ☐ 3 ☐	0 ☐ 1 ☐ 2 ☐ 3 ☐	0 ☐ 1 ☐	0 ☐ 1 ☐ 2 ☐ 3 ☐ 4 ☐	0 ☐ 1 ☐ 2 ☐ 3 ☐	Si ☐ No ☐

Domanda 3

In futuro, si sottoporrebbe a un'altra stimolazione del nervo vago nell'ambito di uno studio di ricerca? Si prega di selezionare "sì" **o** "no".
Si No

☐ ☐

Se ha selezionato „no“, perché non si sottoporrebbe ad un'altra stimolazione? (Per favore riporti il motivo)

___

Domanda 4

In questo studio vengono eseguite sia stimolazioni reali del nervo vago, sia stimolazioni placebo (fittizie). Valuti se in questo ciclo di studio ha ricevuto una stimolazione reale o una stimolazione placebo. Selezioni una delle opzioni.Vera stimolazione Placebo

☐ ☐

Quanto è sicuro/a della sua decisione? Per favore, selezioni una sola casella.

☐ **1** = Per niente sicuro/a
☐ **2** = Poco sicuro/a
☐ **3** = Moderatamente sicuro/a
☐ **4** = Molto sicuro/a
☐ **5** = Completamente sicuro/a

Spazio per ulteriori osservazioni:

___

___

Molte grazie per aver compilato il questionario!

Questionario per il coordinatore dello studio

Mentre il partecipante compila la versione a lui dedicata, si prega di compilare questo questionario per il coordinatore, basato sulle osservazioni fatte durante lo studio.

Domanda 1:

Il questionario è stato compilato completamente e correttamente dal partecipante?

☐ Si ☐ No

Se è stato risposto „no“, richiedere al partecipante di compilarlo completamente.

Domanda 2:

Indicare quale blocco di studio è stato completato. Apporre una croce nella colonna corretta della tabella.

	Ciclo di studio
	stimolazione **Placebo con 30 secondi** di stimolazione elettrica
	stimolazione **Placebo** con **3,4 secondi** di stimolazione elettrica
	stimolazione **Terapeutica** con **30 secondi** di stimolazione elettrica
	stimolazione **Terapeutica** con **3,4 secondi** di stimolazione elettrica

Domanda 3:

Qual è stata l'intensità della stimolazione in questo esperimento? ________ mA

L'intensità è stata modificata durante il ciclo di studio?

☐ Si ⬇ ☐ No ➡ segua con la domanda 4

Nuova intensità ________ mA

Cambio dell'intensità dopo _________ min

Motivo del cambio: ____

Domanda 4:

Ha notato sintomi/eventi indesiderati durante la stimolazione?

____

____

____

Domanda 5:

Si sono verificati sintomi che hanno messo a rischio lo stato di salute della persona sottoposta al test? Selezionare "sì" **o** "no".

☐ No ☐ Si

Se „si“, quali e perché?

____

____

____

Domanda 6:

La stimolazione è stata interrotta a causa di eventi indesiderati? Selezionare "sì" **o** "no".
☐ Si ☐ No

Se „si“, perché?

____

____

____

Domanda 7:

Quali patologie pregresse sono note?

____

Quali farmaci vengono assunti regolarmente?

____

Grazie per la compilazione del questionario!

Nota:

- Veda le nostre raccomandazioni per la valutazione del questionario.
- Veda la checklist di riferimento con le raccomandazioni di Farmer et al. 2020

9.4 TaVNS-AEQ – Spanish version 3.0

<u>Cuestionario sobre Effectos Adversos en Estimulación Transcutánea del Nervio Vago Auricular.</u>

Este cuestionario se utiliza para registrar eventos no deseados durante el estudio. El cuestionario se utiliza únicamente con fines de investigación. Sus datos serán evaluados de forma pseudoanónima. No hay respuesta correcta o incorrecta. Si no está seguro, le pedimos que proporcione la respuesta que más se aplique a su caso. Gracias por su participación.

<u>Pregunta 1</u>

¿Notó algún síntoma de estimulación durante el estudio?
Por favor marque "sí" **o** "no".

☐ Sí ☐ No ➡ Proceda a la pregunta 2.
⬇

Por favor, enumere los síntomas experimentados en la columna de la izquierda y responda a las preguntas correspondientes en las columnas de la derecha de la misma fila para cada síntoma. Por favor, seleccione solo una casilla para cada respuesta. Repita el proceso para todos los síntomas experimentados.

Lista de Síntomas	Nivel de gravedad<br>**1, 2 o 3**<br>1= bajo<br>2= medio / moderado<br>3= alto<br>➡	Punto de inicio<br>**0, 1, 2 o 3**<br>0= Al principio<br>1= A la mitad<br>2= Al final<br>3= Luego de completar esta etapa del estudio<br>➡	Una vez completado el estudio, el síntoma<br>**0 o 1**<br>0= seguía presente<br>1= desapareció<br>➡	Duración total del síntoma<br>**0, 1, 2, 3 o 4**<br>0= menos de 10min<br>1= 11-25min<br>2= 26-49min<br>3= 50min (de inicio a fin)<br>4= sólo en presencia de estimulación eléctrica<br>➡	Posible causa<br>**0, 1, 2 o 3**<br>0= las condiciones / entorno del estudio<br>1= estimulación<br>2= enfermedad previa / síntoma conocido<br>3= al azar sin razón aparente ➡	En general, ¿el síntoma le afecta de forma grave durante el estudio?<br>**Sí o no**

	1 ☐ 2 ☐ 3 ☐	0 ☐ 1 ☐ 2 ☐ 3 ☐	0 ☐ 1 ☐	0 ☐ 1 ☐ 2 ☐ 3 ☐ 4 ☐	0 ☐ 1 ☐ 2 ☐ 3 ☐	Sí ☐ No ☐
	1 ☐ 2 ☐ 3 ☐	0 ☐ 1 ☐ 2 ☐ 3 ☐	0 ☐ 1 ☐	0 ☐ 1 ☐ 2 ☐ 3 ☐ 4 ☐	0 ☐ 1 ☐ 2 ☐ 3 ☐	Sí ☐ No ☐
	1 ☐ 2 ☐ 3 ☐	0 ☐ 1 ☐ 2 ☐ 3 ☐	0 ☐ 1 ☐	0 ☐ 1 ☐ 2 ☐ 3 ☐ 4 ☐	0 ☐ 1 ☐ 2 ☐ 3 ☐	Sí ☐ No ☐
	1 ☐ 2 ☐ 3 ☐	0 ☐ 1 ☐ 2 ☐ 3 ☐	0 ☐ 1 ☐	0 ☐ 1 ☐ 2 ☐ 3 ☐ 4 ☐	0 ☐ 1 ☐ 2 ☐ 3 ☐	Sí ☐ No ☐

Pregunta 2

Lea los síntomas enumerados en la columna izquierda de la tabla en la página siguiente de este cuestionario. **Si usted no experimentó el síntoma en cuestión,** marque con una cruz la casilla debajo de la palabra “no”. En ese caso, no es necesario continuar con las columnas continuas del lado derecho de la tabla. Lea los demás síntomas enumerados y repita el proceso.

Si usted sí experimentó el síntoma en cuestión, marque con una cruz la casilla debajo de la palabra “sí”. Responda todas las preguntas siguientes dentro de la misma fila del síntoma en cuestión. Marque **solamente una casilla** por cada pregunta.

Si tiene alguna duda, diríjase al aplicador de la prueba.

Lista de Síntomas	¿Se presentó el síntoma? **Sí o no** En caso afirmativo ➡	Nivel de gravedad **1, 2 o 3** 1= bajo 2= medio / moderado 3= alto ➡	Punto de inicio **0, 1, 2 o 3** 0= Al principio 1= A la mitad 2= Al final 3= Luego de completar esta etapa del estudio ➡	Una vez completado el estudio, el síntoma **0 o 1** 0= seguía presente 1= desapareció ➡	Duración total del síntoma **0, 1, 2, 3 o 4** 0= menos de 10min 1= 11-25min 2= 26-49min 3= 50min (de inicio a fin) 4= sólo en presencia de estimulación eléctrica ➡	Posible causa **0, 1, 2 o 3** 0= las condiciones / entorno del estudio 1= estimulación 2= enfermedad previa / síntoma conocido 3= al azar sin razón aparente ➡	En general, ¿el síntoma le afecta de forma grave durante el estudio? **Sí o no**
Piel enrojecida	No ☐ ⬇ Sí ☐ ➡	1 ☐ 2 ☐ 3 ☐	0 ☐ 1 ☐ 2 ☐ 3 ☐	0 ☐ 1 ☐	0 ☐ 1 ☐ 2 ☐ 3 ☐ 4 ☐	0 ☐ 1 ☐ 2 ☐ 3 ☐	Sí ☐ No ☐
Sensación punzante	No ☐ ⬇ Sí ☐ ➡	1 ☐ 2 ☐ 3 ☐	0 ☐ 1 ☐ 2 ☐ 3 ☐	0 ☐ 1 ☐	0 ☐ 1 ☐ 2 ☐ 3 ☐ 4 ☐	0 ☐ 1 ☐ 2 ☐ 3 ☐	Sí ☐ No ☐
Sensación de hormigueo en la piel	No ☐ ⬇ Sí ☐ ➡	1 ☐ 2 ☐ 3 ☐	0 ☐ 1 ☐ 2 ☐ 3 ☐	0 ☐ 1 ☐	0 ☐ 1 ☐ 2 ☐ 3 ☐ 4 ☐	0 ☐ 1 ☐ 2 ☐ 3 ☐	Sí ☐ No ☐
Ardor / escozor	No ☐ ⬇ Sí ☐ ➡	1 ☐ 2 ☐ 3 ☐	0 ☐ 1 ☐ 2 ☐ 3 ☐	0 ☐ 1 ☐	0 ☐ 1 ☐ 2 ☐ 3 ☐ 4 ☐	0 ☐ 1 ☐ 2 ☐ 3 ☐	Sí ☐ No ☐
Picazón en la piel	No ☐ ⬇ Sí ☐ ➡	1 ☐ 2 ☐ 3 ☐	0 ☐ 1 ☐ 2 ☐ 3 ☐	0 ☐ 1 ☐	0 ☐ 1 ☐ 2 ☐ 3 ☐ 4 ☐	0 ☐ 1 ☐ 2 ☐ 3 ☐	Sí ☐ No ☐
Sensación de presión sobre la piel	No ☐ ⬇ Sí ☐ ➡	1 ☐ 2 ☐ 3 ☐	0 ☐ 1 ☐ 2 ☐ 3 ☐	0 ☐ 1 ☐	0 ☐ 1 ☐ 2 ☐ 3 ☐ 4 ☐	0 ☐ 1 ☐ 2 ☐ 3 ☐	Sí ☐ No ☐

Lista de Síntomas	¿Se presentó el síntoma? **Sí o no** En caso afirmativo ➡	Nivel de gravedad **1, 2 o 3** 1= bajo 2= medio / moderado 3= alto ➡	Punto de inicio **0, 1, 2 o 3** 0= Al principio 1= A la mitad 2= Al final 3= Luego de completar esta etapa del estudio ➡	Una vez completado el estudio, el síntoma **0 o 1** 0= seguía presente 1= desapareció ➡	Duración total del síntoma **0, 1, 2, 3 o 4** 0= menos de 10min 1= 11-25min 2= 26-49min 3= 50min (de inicio a fin) 4= sólo en presencia de estimulación eléctrica ➡	Posible causa **0, 1, 2 o 3** 0= las condiciones / entorno del estudio 1= estimulación 2= enfermedad previa / síntoma conocido 3= al azar sin razón aparente ➡	En general, ¿el síntoma le afecta de forma grave durante el estudio? **Sí o no**
Otra sensación desagradable en la piel	No ☐ ⬇ Sí ☐ ➡	1 ☐ 2 ☐ 3 ☐	0 ☐ 1 ☐ 2 ☐ 3 ☐	0 ☐ 1 ☐	0 ☐ 1 ☐ 2 ☐ 3 ☐ 4 ☐	0 ☐ 1 ☐ 2 ☐ 3 ☐	Sí ☐ No ☐
Dolor en la piel	No ☐ ⬇ Sí ☐ ➡	1 ☐ 2 ☐ 3 ☐	0 ☐ 1 ☐ 2 ☐ 3 ☐	0 ☐ 1 ☐	0 ☐ 1 ☐ 2 ☐ 3 ☐ 4 ☐	0 ☐ 1 ☐ 2 ☐ 3 ☐	Sí ☐ No ☐
Problemas de oído (p.ej.taponamiento o presión)	No ☐ ⬇ Sí ☐ ➡	1 ☐ 2 ☐ 3 ☐	0 ☐ 1 ☐ 2 ☐ 3 ☐	0 ☐ 1 ☐	0 ☐ 1 ☐ 2 ☐ 3 ☐ 4 ☐	0 ☐ 1 ☐ 2 ☐ 3 ☐	Sí ☐ No ☐
Tinnitus / zumbido en los oídos	No ☐ ⬇ Sí ☐ ➡	1 ☐ 2 ☐ 3 ☐	0 ☐ 1 ☐ 2 ☐ 3 ☐	0 ☐ 1 ☐	0 ☐ 1 ☐ 2 ☐ 3 ☐ 4 ☐	0 ☐ 1 ☐ 2 ☐ 3 ☐	Sí ☐ No ☐
Dolor de cuello	No ☐ ⬇ Sí ☐ ➡	1 ☐ 2 ☐ 3 ☐	0 ☐ 1 ☐ 2 ☐ 3 ☐	0 ☐ 1 ☐	0 ☐ 1 ☐ 2 ☐ 3 ☐ 4 ☐	0 ☐ 1 ☐ 2 ☐ 3 ☐	Sí ☐ No ☐
Espasmos musculares en la cara y/o cuello	No ☐ ⬇ Sí ☐ ➡	1 ☐ 2 ☐ 3 ☐	0 ☐ 1 ☐ 2 ☐ 3 ☐	0 ☐ 1 ☐	0 ☐ 1 ☐ 2 ☐ 3 ☐ 4 ☐	0 ☐ 1 ☐ 2 ☐ 3 ☐	Sí ☐ No ☐
Mareo	No ☐ ⬇ Sí ☐ ➡	1 ☐ 2 ☐ 3 ☐	0 ☐ 1 ☐ 2 ☐ 3 ☐	0 ☐ 1 ☐	0 ☐ 1 ☐ 2 ☐ 3 ☐ 4 ☐	0 ☐ 1 ☐ 2 ☐ 3 ☐	Sí ☐ No ☐

Lista de Síntomas	¿Se presentó el síntoma? **Sí o no** En caso afirmativo ➡	Nivel de gravedad **1, 2 o 3** 1= bajo 2= medio / moderado 3= alto ➡	Punto de inicio **0, 1, 2 o 3** 0= Al principio 1= A la mitad 2= Al final 3= Luego de completar esta etapa del estudio ➡	Una vez completado el estudio, el síntoma **0 o 1** 0= seguía presente 1= desapareció ➡	Duración total del síntoma **0, 1, 2, 3 o 4** 0= menos de 10min 1= 11-25min 2= 26-49min 3= 50min (de inicio a fin) 4= sólo en presencia de estimulación eléctrica ➡	Posible causa **0, 1, 2 o 3** 0= las condiciones / entorno del estudio 1= estimulación 2= enfermedad previa / síntoma conocido 3= al azar sin razón aparente ➡	En general, ¿el síntoma le afecta de forma grave durante el estudio? **Sí o no**
Dolor de cabeza	No ☐ ⬇ Sí ☐ ➡	1 ☐ 2 ☐ 3 ☐	0 ☐ 1 ☐ 2 ☐ 3 ☐	0 ☐ 1 ☐	0 ☐ 1 ☐ 2 ☐ 3 ☐ 4 ☐	0 ☐ 1 ☐ 2 ☐ 3 ☐	Sí ☐ No ☐
Cambios en la concentración	No ☐ ⬇ Sí ☐ ➡	1 ☐ 2 ☐ 3 ☐	0 ☐ 1 ☐ 2 ☐ 3 ☐	0 ☐ 1 ☐	0 ☐ 1 ☐ 2 ☐ 3 ☐ 4 ☐	0 ☐ 1 ☐ 2 ☐ 3 ☐	Sí ☐ No ☐
Fluctuación de los sentimientos	No ☐ ⬇ Sí ☐ ➡	1 ☐ 2 ☐ 3 ☐	0 ☐ 1 ☐ 2 ☐ 3 ☐	0 ☐ 1 ☐	0 ☐ 1 ☐ 2 ☐ 3 ☐ 4 ☐	0 ☐ 1 ☐ 2 ☐ 3 ☐	Sí ☐ No ☐
Fatiga / cansancio	No ☐ ⬇ Sí ☐ ➡	1 ☐ 2 ☐ 3 ☐	0 ☐ 1 ☐ 2 ☐ 3 ☐	0 ☐ 1 ☐	0 ☐ 1 ☐ 2 ☐ 3 ☐ 4 ☐	0 ☐ 1 ☐ 2 ☐ 3 ☐	Sí ☐ No ☐
Estado de activación	No ☐ ⬇ Sí ☐ ➡	1 ☐ 2 ☐ 3 ☐	0 ☐ 1 ☐ 2 ☐ 3 ☐	0 ☐ 1 ☐	0 ☐ 1 ☐ 2 ☐ 3 ☐ 4 ☐	0 ☐ 1 ☐ 2 ☐ 3 ☐	Sí ☐ No ☐
Malestar (inespecífico)	No ☐ ⬇ Sí ☐ ➡	1 ☐ 2 ☐ 3 ☐	0 ☐ 1 ☐ 2 ☐ 3 ☐	0 ☐ 1 ☐	0 ☐ 1 ☐ 2 ☐ 3 ☐ 4 ☐	0 ☐ 1 ☐ 2 ☐ 3 ☐	Sí ☐ No ☐
Dolor en el pecho Insomnio	No ☐ ⬇ Sí ☐ ➡	1 ☐ 2 ☐ 3 ☐	0 ☐ 1 ☐ 2 ☐ 3 ☐	0 ☐ 1 ☐	0 ☐ 1 ☐ 2 ☐ 3 ☐ 4 ☐	0 ☐ 1 ☐ 2 ☐ 3 ☐	Sí ☐ No ☐

Lista de Síntomas	¿Se presentó el síntoma? **Sí o no** En caso afirmativo ➡	Nivel de gravedad **1, 2 o 3** 1= bajo 2= medio / moderado 3= alto ➡	Punto de inicio **0, 1, 2 o 3** 0= Al principio 1= A la mitad 2= Al final 3= Luego de completar esta etapa del estudio ➡	Una vez completado el estudio, el síntoma **0 o 1** 0= seguía presente 1= desapareció ➡	Duración total del síntoma **0, 1, 2, 3 o 4** 0= menos de 10min 1= 11-25min 2= 26-49min 3= 50min (de inicio a fin) 4= sólo en presencia de estimulación eléctrica ➡	Posible causa **0, 1, 2 o 3** 0= las condiciones / entorno del estudio 1= estimulación 2= enfermedad previa / síntoma conocido 3= al azar sin razón aparente ➡	En general, ¿el síntoma le afecta de forma grave durante el estudio? **Sí o no**
Síntomas cardiovasculares (p.ej. palpitaciones, debilidad, colapso)	No ☐ ⬇ Sí ☐ ➡	1 ☐ 2 ☐ 3 ☐	0 ☐ 1 ☐ 2 ☐ 3 ☐	0 ☐ 1 ☐	0 ☐ 1 ☐ 2 ☐ 3 ☐ 4 ☐	0 ☐ 1 ☐ 2 ☐ 3 ☐	Sí ☐ No ☐
Náuseas	No ☐ ⬇ Sí ☐ ➡	1 ☐ 2 ☐ 3 ☐	0 ☐ 1 ☐ 2 ☐ 3 ☐	0 ☐ 1 ☐	0 ☐ 1 ☐ 2 ☐ 3 ☐ 4 ☐	0 ☐ 1 ☐ 2 ☐ 3 ☐	Sí ☐ No ☐
Dolor de estómago	No ☐ ⬇ Sí ☐ ➡	1 ☐ 2 ☐ 3 ☐	0 ☐ 1 ☐ 2 ☐ 3 ☐	0 ☐ 1 ☐	0 ☐ 1 ☐ 2 ☐ 3 ☐ 4 ☐	0 ☐ 1 ☐ 2 ☐ 3 ☐	Sí ☐ No ☐
Constipación	No ☐ ⬇ Sí ☐ ➡	1 ☐ 2 ☐ 3 ☐	0 ☐ 1 ☐ 2 ☐ 3 ☐	0 ☐ 1 ☐	0 ☐ 1 ☐ 2 ☐ 3 ☐ 4 ☐	0 ☐ 1 ☐ 2 ☐ 3 ☐	Sí ☐ No ☐
Insomnio	No ☐ ⬇ Sí ☐ ➡	1 ☐ 2 ☐ 3 ☐	0 ☐ 1 ☐ 2 ☐ 3 ☐	0 ☐ 1 ☐	0 ☐ 1 ☐ 2 ☐ 3 ☐ 4 ☐	0 ☐ 1 ☐ 2 ☐ 3 ☐	Sí ☐ No ☐

Pregunta 3

¿Estaría usted dispuesto a participar en otro estudio sobre estimulación del nervio vago? Marque "sí" o "no" con una cruz.

Sí No

☐ ☐

Si usted ha marcado "no", ¿por qué no estaría dispuesto a recibir más estimulación del nervio vago? (Por favor indique el motivo)

Pregunta 4

En el marco de este estudio se lleva a cabo una estimulación terapéutica, es decir, verdadera, del nervio vago, así como una estimulación placebo (estimulación simulada). Evalúe si este estudio presumiblemente fue una estimulación terapéutica del nervio vago o una estimulación con placebo. Marque una de las dos opciones.

Estimulación verdadera Placebo

☐ ☐

¿Qué tan seguro(a) se siente de su decisión? Por favor, marque solo una casilla.

☐ **1** = Nada seguro(a)
☐ **2** = Poco seguro(a)
☐ **3** = Moderadamente seguro(a)
☐ **4** = Muy seguro(a)
☐ **5** = Completamente seguro(a)

Espacio para más comentarios:

¡Muchas gracias por responder el cuestionario!

Cuestionario para Aplicadores de Prueba

Mientras el participante completa el cuestionario para participantes, por favor complete este cuestionario para aplicadores de prueba según las observaciones realizadas durante el estudio.

Pregunta 1:

¿Se ha respondido al cuestionario de forma completa y correcta?

☐ Sí ☐ No

En caso negativo, pídale al participante que lo complete de forma integral.

Pregunta 2:

Indique cuál de los bloques de estudio se llevó a cabo. Para ello, marque con una cruz la columna correcta de la tabla.

	Ronda de estudio
	Estimulación **placebo** con **30 segundos** de aplicación de corriente eléctrica
	Estimulación **placebo** con **3.4 segundos** de aplicación de corriente eléctrica
	Estimulación **terapéutica** con **30 segundos** de aplicación de corriente eléctrica
	Estimulación **terapéutica** con **3.4 segundos** de aplicación de corriente eléctrica

Pregunta 3:

¿Con qué intensidad se aplicó la estimulación en esta ronda de estudio? ________ mA

¿Se realizó algún cambio en la intensidad durante esta ronda?

☐ Sí ⬇ ☐ No ➡ pase a la pregunta 4

Nueva intensidad: ________ mA

Cambio de intensidad después de ________ min

Razón del cambio: ____

Pregunta 4:

¿Notó algún síntoma / evento no deseado durante la estimulación?

____

____

____

Pregunta 5:

¿Hubo algún síntoma que pusiera en grave peligro la salud del participante? Por favor marque "sí" **o** "no".

☐ No ☐ Sí

En caso afirmativo, ¿cuáles y por qué?

____

____

____

Pregunta 6:

¿Fue necesario suspender la estimulación debido a eventos no deseados? Por favor marque "sí" **o** "no".

☐ Sí ☐ No

En caso afirmativo, ¿por qué?

____

____

____

Pregunta 7:

¿Qué enfermedades preexistentes se conocen?

____

¿Qué medicamentos se toman regularmente?

____

¡Gracias por completar el cuestionario!

Observaciones:

- Consulte las recomendaciones para evaluar el cuestionario.
- Consulte la lista de verificación de recomendaciones para la presentación de informes de Farmer et al. 2020

9.5 TaVNS-AEQ – Frensh version 3.0

<u>Questionnaire sur la stimulation transcutanée du nerf vague auriculaire</u>

TaVNS-AEQ

Ce questionnaire vise à recueillir des événements indésirables survenant pendant la réalisation de l'étude. Ce questionnaire sera utilisé uniquement à des fins de recherche. Vos données seront analysées de manière pseudo-anonyme.
Il n'y a pas de réponse "juste ou fausse". En cas d'incertitude, nous vous demandons de choisir la réponse qui vous semble la plus appropriée.
Nous vous remercions pour votre participation.

<u>Question 1</u>

Avez-vous remarqué des symptômes de stimulation pendant la réalisation de l'étude ?

Veuillez cocher „oui“ **ou** „non“.

☐ Oui ☐ Non ➡ Veuillez passer à la question 2.

⬇

Veuillez indiquer les symptômes ressentis dans la colonne de gauche, puis répondre aux questions correspondantes dans les colonnes de droite pour chaque symptôme. Veuillez cocher une seule case pour chaque réponse. Procédez de la même façon pour chacun des symptômes ressentis.

Liste des symptômes	Degré de gravité **1, 2 ou 3**<br>1= léger<br>2= moyen/ modéré<br>3= grave ➡	Moment d'apparition du symptôme<br>**0, 1, 2 ou 3**<br>0 = au début<br>1 = au milieu<br>2 = à la fin<br>3 = après la participation à l'étude ➡	Après la participation à l'étude<br>**0 ou 1**<br>0 = symptôme encore présent<br>1 = Symptôme disparu ➡	Durée totale du symptôme<br>**0, 1, 2, 3 ou 4**<br>0 = moins de 10min<br>1 = entre 10-25min<br>2 = entre 26-49min<br>3 = plus de 50min (du début à la fin)<br>4 = seulement lors de la stimulation électrique ➡	Cause probable<br>**0,1, 2 ou 3**<br>0= dans le cadre de l'étude / de la participation à l'étude<br>1= la stimulation<br>2= antécédent médical / Symptôme connu<br>3= aléatoire, sans raison identifiable ➡	Symptôme particulièrement gênant pendant la participation à l'étude ?<br>**Oui ou non**
	1 ☐ 2 ☐ 3 ☐	0 ☐ 1 ☐ 2 ☐ 3 ☐	0 ☐ 1 ☐	0 ☐ 1 ☐ 2 ☐ 3 ☐ 4 ☐	0 ☐ 1 ☐ 2 ☐ 3 ☐	Oui ☐ Non ☐
	1 ☐ 2 ☐ 3 ☐	0 ☐ 1 ☐ 2 ☐ 3 ☐	0 ☐ 1 ☐	0 ☐ 1 ☐ 2 ☐ 3 ☐ 4 ☐	0 ☐ 1 ☐ 2 ☐ 3 ☐	Oui ☐ Non ☐
	1 ☐ 2 ☐ 3 ☐	0 ☐ 1 ☐ 2 ☐ 3 ☐	0 ☐ 1 ☐	0 ☐ 1 ☐ 2 ☐ 3 ☐ 4 ☐	0 ☐ 1 ☐ 2 ☐ 3 ☐	Oui ☐ Non ☐
	1 ☐ 2 ☐ 3 ☐	0 ☐ 1 ☐ 2 ☐ 3 ☐	0 ☐ 1 ☐	0 ☐ 1 ☐ 2 ☐ 3 ☐ 4 ☐	0 ☐ 1 ☐ 2 ☐ 3 ☐	Oui ☐ Non ☐

Question 2

Sur la page suivante de ce questionnaire, veuillez lire les symptômes énumérés dans la colonne de gauche. **Si le symptôme ne s'est pas manifesté chez vous**, veuillez cocher la case "non". Si « non », veuillez ne pas compléter les colonnes de droite du tableau. Pour chaque symptôme de la liste, veuillez répéter cette procédure. **Si vous avez ressenti le symptôme**, veuillez alors cocher la case "oui". Ensuite répondez à toutes les questions supplémentaires correspondant au symptôme ressenti. Cochez **une seule case pour chaque question** posée.

Si vous avez des questions, n'hésitez pas à contacter les responsables de l'étude.

Liste des symptômes	Symptôme présent ? **Oui ou non** Si oui ➡	Degré de gravité **1, 2 ou 3** 1= léger 2= moyen/ modéré 3= grave ➡	Moment d'apparition du symptôme **0, 1, 2 ou 3** 0 = au début 1 = au milieu 2 = à la fin 3 = après la participation à l'étude ➡	Après la participation à l'étude **0 ou 1** 0=symptôme encore présent 1=Symptôme disparu ➡	Durée totale du symptôme **0, 1, 2, 3 ou 4** 0 = moins de 10min 1 = entre 10-25min 2 = entre 26-49min 3 = plus de 50min (du début à la fin) 4= seulement lors de la stimulation électrique ➡	Cause probable **0,1, 2 ou 3** 0= dans le cadre de l'étude / de la participation à l'étude 1= la stimulation 2= antécédent médical / Symptôme connu 3= aléatoire sans raison identifiable ➡	Symptôme particulièrement gênant pendant la participation à l'étude ? **Oui ou non**
Rougeurs sur la peau	Non ☐ ⬇ Oui ☐ ➡	1 ☐ 2 ☐ 3 ☐	0 ☐ 1 ☐ 2 ☐ 3 ☐	0 ☐ 1 ☐	0 ☐ 1 ☐ 2 ☐ 3 ☐ 4 ☐	0 ☐ 1 ☐ 2 ☐ 3 ☐	Oui ☐ Non ☐
Sensation de piqûre sur la peau	Non ☐ ⬇ Oui ☐ ➡	1 ☐ 2 ☐ 3 ☐	0 ☐ 1 ☐ 2 ☐ 3 ☐	0 ☐ 1 ☐	0 ☐ 1 ☐ 2 ☐ 3 ☐ 4 ☐	0 ☐ 1 ☐ 2 ☐ 3 ☐	Oui ☐ Non ☐
Sensation de picotement de la peau	Nein ☐ ⬇ Oui ☐ ➡	1 ☐ 2 ☐ 3 ☐	0 ☐ 1 ☐ 2 ☐ 3 ☐	0 ☐ 1 ☐	0 ☐ 1 ☐ 2 ☐ 3 ☐ 4 ☐	0 ☐ 1 ☐ 2 ☐ 3 ☐	Oui ☐ Non ☐
Sensation de brûlure de la peau	Non ☐ ⬇ Oui ☐ ➡	1 ☐ 2 ☐ 3 ☐	0 ☐ 1 ☐ 2 ☐ 3 ☐	0 ☐ 1 ☐	0 ☐ 1 ☐ 2 ☐ 3 ☐ 4 ☐	0 ☐ 1 ☐ 2 ☐ 3 ☐	Oui ☐ Non ☐
Démangeaisons sur la peau	Non ☐ ⬇ Oui ☐ ➡	1 ☐ 2 ☐ 3 ☐	0 ☐ 1 ☐ 2 ☐ 3 ☐	0 ☐ 1 ☐	0 ☐ 1 ☐ 2 ☐ 3 ☐ 4 ☐	0 ☐ 1 ☐ 2 ☐ 3 ☐	Oui ☐ Non ☐
Sensation de pression sur la peau	Non ☐ ⬇ Oui ☐ ➡	1 ☐ 2 ☐ 3 ☐	0 ☐ 1 ☐ 2 ☐ 3 ☐	0 ☐ 1 ☐	0 ☐ 1 ☐ 2 ☐ 3 ☐ 4 ☐	0 ☐ 1 ☐ 2 ☐ 3 ☐	Oui ☐ Non ☐

Liste des symptômes	Symptôme présent ? **Oui ou non** Si oui ➡	Degré de gravité **1, 2 ou 3**<br>1= léger<br>2= moyen/ modéré<br>3= grave ➡	Moment d'apparition du symptôme **0, 1, 2 ou 3**<br>0 = au début<br>1 = au milieu<br>2 = à la fin<br>3 = après la participation à l'étude ➡	Après la participation à l'étude **0 ou 1**<br>0=symptôme encore présent<br>1=Symptôme disparu ➡	Durée totale du symptôme **0, 1, 2, 3 ou 4**<br>0 = moins de 10min<br>1 = entre 10-25min<br>2 = entre 26-49min<br>3 = plus de 50min (du début à la fin)<br>4= seulement lors de la stimulation électrique ➡	Cause probable **0,1, 2 ou 3**<br>0= dans le cadre de l'étude / de la participation à l'étude<br>1= la stimulation<br>2= antécédent médical / Symptôme connu<br>3= aléatoire sans raison identifiable ➡	Symptôme particulièrement gênant pendant la participation à l'étude ? **Oui ou non**
autre sensation désagréable sur la peau	Non ☐ ⬇ Oui ☐ ➡	1 ☐ 2 ☐ 3 ☐	0 ☐ 1 ☐ 2 ☐ 3 ☐	0 ☐ 1 ☐	0 ☐ 1 ☐ 2 ☐ 3 ☐ 4 ☐	0 ☐ 1 ☐ 2 ☐ 3 ☐	Oui ☐ Non ☐
Douleurs de la peau	Non ☐ ⬇ Oui ☐ ➡	1 ☐ 2 ☐ 3 ☐	0 ☐ 1 ☐ 2 ☐ 3 ☐	0 ☐ 1 ☐	0 ☐ 1 ☐ 2 ☐ 3 ☐ 4 ☐	0 ☐ 1 ☐ 2 ☐ 3 ☐	Oui ☐ Non ☐
Maux d'oreilles (p.ex. sensation d'oreilles bouchées ou de pression auriculaire)	Non ☐ ⬇ Oui ☐ ➡	1 ☐ 2 ☐ 3 ☐	0 ☐ 1 ☐ 2 ☐ 3 ☐	0 ☐ 1 ☐	0 ☐ 1 ☐ 2 ☐ 3 ☐ 4 ☐	0 ☐ 1 ☐ 2 ☐ 3 ☐	Oui ☐ Non ☐
Acouphènes, bruit dans l'oreille	Non ☐ ⬇ Oui ☐ ➡	1 ☐ 2 ☐ 3 ☐	0 ☐ 1 ☐ 2 ☐ 3 ☐	0 ☐ 1 ☐	0 ☐ 1 ☐ 2 ☐ 3 ☐ 4 ☐	0 ☐ 1 ☐ 2 ☐ 3 ☐	Oui ☐ Non ☐
Douleurs cervicales	Non ☐ ⬇ Oui ☐ ➡	1 ☐ 2 ☐ 3 ☐	0 ☐ 1 ☐ 2 ☐ 3 ☐	0 ☐ 1 ☐	0 ☐ 1 ☐ 2 ☐ 3 ☐ 4 ☐	0 ☐ 1 ☐ 2 ☐ 3 ☐	Oui ☐ Non ☐
Crampes musculaires au visage et/ou à la nuque	Non ☐ ⬇ Oui ☐ ➡	1 ☐ 2 ☐ 3 ☐	0 ☐ 1 ☐ 2 ☐ 3 ☐	0 ☐ 1 ☐	0 ☐ 1 ☐ 2 ☐ 3 ☐ 4 ☐	0 ☐ 1 ☐ 2 ☐ 3 ☐	Oui ☐ Non ☐

Liste des symptômes	Symptôme présent ? **Oui ou non** Si oui ➡	Degré de gravité **1, 2 ou 3**<br>1= léger<br>2= moyen/ modéré<br>3= grave ➡	Moment d'apparition du symptôme **0, 1, 2 ou 3**<br>0 = au début<br>1 = au milieu<br>2 = à la fin<br>3 = après la participation à l'étude ➡	Après la participation à l'étude **0 ou 1**<br>0=symptôme encore présent<br>1=Symptôme disparu ➡	Durée totale du symptôme **0, 1, 2, 3 ou 4**<br>0 = moins de 10min<br>1 = entre 10-25min<br>2 = entre 26-49min<br>3 = plus de 50min (du début à la fin)<br>4= seulement lors de la stimulation électrique ➡	Cause probable **0,1, 2 ou 3**<br>0= dans le cadre de l'étude / de la participation à l'étude<br>1= la stimulation<br>2= antécédent médical / Symptôme connu<br>3= aléatoire sans raison identifiable ➡	Symptôme particulièrement gênant pendant la participation à l'étude ? **Oui ou non**
Vertiges/étourdissements	Non ☐ ⬇ Oui ☐ ➡	1 ☐ 2 ☐ 3 ☐	0 ☐ 1 ☐ 2 ☐ 3 ☐	0 ☐ 1 ☐	0 ☐ 1 ☐ 2 ☐ 3 ☐ 4 ☐	0 ☐ 1 ☐ 2 ☐ 3 ☐	Oui ☐ Non ☐
Maux de tête	Non ☐ ⬇ Oui ☐ ➡	1 ☐ 2 ☐ 3 ☐	0 ☐ 1 ☐ 2 ☐ 3 ☐	0 ☐ 1 ☐	0 ☐ 1 ☐ 2 ☐ 3 ☐ 4 ☐	0 ☐ 1 ☐ 2 ☐ 3 ☐	Oui ☐ Non ☐
Changements de capacité concentration	Non ☐ ⬇ Oui ☐ ➡	1 ☐ 2 ☐ 3 ☐	0 ☐ 1 ☐ 2 ☐ 3 ☐	0 ☐ 1 ☐	0 ☐ 1 ☐ 2 ☐ 3 ☐ 4 ☐	0 ☐ 1 ☐ 2 ☐ 3 ☐	Oui ☐ Non ☐
Fluctuation des sentiments / sensations	Non ☐ ⬇ Oui ☐ ➡	1 ☐ 2 ☐ 3 ☐	0 ☐ 1 ☐ 2 ☐ 3 ☐	0 ☐ 1 ☐	0 ☐ 1 ☐ 2 ☐ 3 ☐ 4 ☐	0 ☐ 1 ☐ 2 ☐ 3 ☐	Oui ☐ Non ☐
Fatigue	Non ☐ ⬇ Oui ☐ ➡	1 ☐ 2 ☐ 3 ☐	0 ☐ 1 ☐ 2 ☐ 3 ☐	0 ☐ 1 ☐	0 ☐ 1 ☐ 2 ☐ 3 ☐ 4 ☐	0 ☐ 1 ☐ 2 ☐ 3 ☐	Oui ☐ Non ☐
État d'excitation	Non ☐ ⬇ Oui ☐ ➡	1 ☐ 2 ☐ 3 ☐	0 ☐ 1 ☐ 2 ☐ 3 ☐	0 ☐ 1 ☐	0 ☐ 1 ☐ 2 ☐ 3 ☐ 4 ☐	0 ☐ 1 ☐ 2 ☐ 3 ☐	Oui ☐ Non ☐

Liste des symptômes	Symptôme présent ? **Oui ou non** Si oui ➡	Degré de gravité **1, 2 ou 3** 1= léger 2= moyen/ modéré 3= grave ➡	Moment d'apparition du symptôme **0, 1, 2 ou 3** 0 = au début 1 = au milieu 2 = à la fin 3 = après la participation à l'étude ➡	Après la participation à l'étude **0 ou 1** 0=symptôm e encore présent 1=Symptôm e disparu ➡	Durée totale du symptôme **0, 1, 2, 3 ou 4** 0 = moins de 10min 1 = entre 10-25min 2 = entre 26-49min 3 = plus de 50min (du début à la fin) 4= seulement lors de la stimulation électrique ➡	Cause probable **0,1, 2 ou 3** 0= dans le cadre de l'étude / de la participation à l'étude 1= la stimulation 2= antécédent médical / Symptôme connu 3= aléatoire sans raison identifiable ➡	Symptôme particulière ment gênant pendant la participation à l'étude ? **Oui ou non**
Malaise (non spécifié)	Non ☐ ⬇ Oui ☐ ➡	1 ☐ 2 ☐ 3 ☐	0 ☐ 1 ☐ 2 ☐ 3 ☐	0 ☐ 1 ☐	0 ☐ 1 ☐ 2 ☐ 3 ☐ 4 ☐	0 ☐ 1 ☐ 2 ☐ 3 ☐	Oui ☐ Non ☐
Douleurs thoraciques	Non ☐ ⬇ Oui ☐ ➡	1 ☐ 2 ☐ 3 ☐	0 ☐ 1 ☐ 2 ☐ 3 ☐	0 ☐ 1 ☐	0 ☐ 1 ☐ 2 ☐ 3 ☐ 4 ☐	0 ☐ 1 ☐ 2 ☐ 3 ☐	Oui ☐ Non ☐
Symptômes cardiovasculaires (p.ex.palpitations, faiblesse, perte de connaissance)	Non ☐ ⬇ Oui ☐ ➡	1 ☐ 2 ☐ 3 ☐	0 ☐ 1 ☐ 2 ☐ 3 ☐	0 ☐ 1 ☐	0 ☐ 1 ☐ 2 ☐ 3 ☐ 4 ☐	0 ☐ 1 ☐ 2 ☐ 3 ☐	Oui ☐ Non ☐
Nausée	Non ☐ ⬇ Oui ☐ ➡	1 ☐ 2 ☐ 3 ☐	0 ☐ 1 ☐ 2 ☐ 3 ☐	0 ☐ 1 ☐	0 ☐ 1 ☐ 2 ☐ 3 ☐ 4 ☐	0 ☐ 1 ☐ 2 ☐ 3 ☐	Oui ☐ Non ☐
Douleur à l'estomac	Non ☐ ⬇ Oui ☐ ➡	1 ☐ 2 ☐ 3 ☐	0 ☐ 1 ☐ 2 ☐ 3 ☐	0 ☐ 1 ☐	0 ☐ 1 ☐ 2 ☐ 3 ☐ 4 ☐	0 ☐ 1 ☐ 2 ☐ 3 ☐	Oui ☐ Non ☐
Constipation	Non ☐ ⬇ Oui ☐ ➡	1 ☐ 2 ☐ 3 ☐	0 ☐ 1 ☐ 2 ☐ 3 ☐	0 ☐ 1 ☐	0 ☐ 1 ☐ 2 ☐ 3 ☐ 4 ☐	0 ☐ 1 ☐ 2 ☐ 3 ☐	Oui ☐ Non ☐
Insomnie	Non ☐ ⬇ Oui ☐ ➡	1 ☐ 2 ☐ 3 ☐	0 ☐ 1 ☐ 2 ☐ 3 ☐	0 ☐ 1 ☐	0 ☐ 1 ☐ 2 ☐ 3 ☐ 4 ☐	0 ☐ 1 ☐ 2 ☐ 3 ☐	Oui ☐ Non ☐

Question 3

Accepteriez-vous de recevoir une autre stimulation du nerf vague dans le contexte de la participation à une étude ? Veuillez cocher "oui" **ou** "non".

☐ Oui ☐ Non

Si vous avez coché "non", pour quelle raison refuseriez-vous de recevoir une autre stimulation ? (Pouvez-vous développer, s’il-vous-plaît ?)

Question 4

Dans le cadre de cette étude, des stimulations thérapeutiques, c'est-à-dire réelles, du nerf vague ainsi que des stimulations placebo (stimulations fictives) sont réalisées. Indiquez si cette séance d'étude correspond vraisemblablement à une stimulation thérapeutique du nerf vague ou à une stimulation placebo. Cochez **l'une** des deux options.

Vraie stimulation Placebo

☐ ☐

À quel point êtes-vous sûr(e) de votre décision ? Veuillez cocher une seule case.

☐ **1** = Pas du tout sûr(e)
☐ **2** = Légèrement sûr(e)
☐ **3** = Modérément sûr(e)
☐ **4** = Très sûr(e)
☐ **5** = Complètement sûr(e)

Espace pour commentaires supplémentaires :

Merci beaucoup d’avoir répondu à ce questionnaire !

Questionnaire pour l’investigateur

Pendant que le participant remplit le questionnaire destiné aux participants, veuillez compléter ce questionnaire destiné aux investigateurs sur la base des observations faites au cours de l'étude.

<u>Question 1:</u>

Le questionnaire a-t-il été complètement et correctement rempli ?

☐ Oui ☐ Non

Si "Non" est coché, veuillez demander au participant de compléter le questionnaire.

<u>Question 2:</u>

Veuillez indiquer quelle séance d'étude a été exécutée. Cochez la case correspondante dans la colonne appropriée du tableau.

	séance d'étude
	**Stimulation placebo** avec une administration de courant de **30 secondes**
	**Stimulation placebo** avec une administration de courant de **3,4 secondes**
	Stimulation **thérapeutique** avec une administration de courant de **30 secondes**
	Stimulation **thérapeutique** avec une administration de courant de **3,4 secondes**

Question 3 :

Quelle intensité de stimulation a été utilisée cette séance d'étude ? _______ mA

L'intensité a-t-elle été modifiée pendant la séance ?

☐ Oui ☐ Non ➡ passez à la question: 4

⬇

Nouvelle intensité : _______ mA

Modification de l'intensité après________ min

Raison de cette modification :____

Question 4 :

Des symptômes ou des événements indésirables ont-ils été observés au cours de la stimulation ?

____

____

____

Question 5:

Des symptômes mettant sérieusement en danger l'état de santé du participant ont-ils été constatés ? Veuillez cocher « oui » **ou** « non ».

☐ Non ☐ Oui

Si oui, lesquels et pour quelle raison ?

___

___

___

<u>Question 6:</u>

La stimulation a-t-elle été interrompue en raison d'événements indésirables ? Veuillez cocher « oui » ou « non ».

☐ Oui ☐ Non

Si oui, lesquels et pour quelle raison ?

___

___

___

<u>Question 7:</u>

Quelles pathologies préexistantes sont connues ?

___

Quels médicaments sont pris régulièrement ?

___

Nous vous remercions d'avoir complété ce questionnaire !

Remarques:

- Voir les recommandations pour l'analyse du questionnaire.
- Voir la liste de vérification pour les recommandations de rapport de Farmer et al. 2020

9.6 Instructions for application and analysis of questionnaire

Before application

- Could this subject group face specific symptoms that should be added to the questionnaire?
- Could the stimulation setting, study type or way of stimulation application lead to a different symptom spectrum and why?

During application

- Hand out the questionnaire to the subjects right after stimulation in short term studies. If taVNS is applied over an extended period, plan the time periods for distributing the questionnaire.
- Do not comment the questionnaire in order to obtain a standardized condition. Only answer when questions are asked.
- While subjects complete the questionnaire, the test leaders should also start to fill out the question part.
- After subjects complete their questionnaires, check them for any missing information, and kindly bring attention to any omissions.

Information to analyze and to mention in study paper

- Absolute numbers
    - for every symptom (also when 0)
    - for each study group
    - of people experiencing the symptom
- severity level
- time of onset
- duration
- which symptoms impair the subject heavily
- potential reason for symptom
- whether proband would engage in another taVNS study
- blinding situation in study
- observation and additional symptoms of study coordinators
- study drop-outs and reason
- how many reductions of stimulation intensity during study and reason
- health-threatening symptoms and detailed description
- stimulation setting (checklist from Farmer et al. (2020))
- detection method description

Supplementary Material 10

TaVNS-AEQ – Old German version 2.0 used in study

Fragebogen zur transkutanen aurikulären Vagusnervstimulation - alte Version aus Studie

Dieser Fragebogen dient dazu unerwünschte Ereignisse während der Studiendurchführung zu erfassen. Der Fragebogen wird nur für Forschungszwecke verwendet. Ihre Daten werden pseudoanonym ausgewertet.

Bei den Antworten gibt es kein „Richtig oder Falsch". Bei Unsicherheiten bitten wir Sie eine Antwort anzugeben, die am meisten auf Sie zutrifft. Vielen Dank für Ihre Mithilfe.

Frage 1

Haben Sie Symptome der Stimulation während der Studiendurchführung bemerkt?

Bitte „ja" **oder** „nein" ankreuzen.

☐ Ja ☐ Nein

Frage 2

Bitte lesen Sie die aufgelisteten Symptome in der linken Spalte der Tabelle auf der nächsten Seite dieses Fragebogens. **Wenn das Symptom bei Ihnen nicht aufgetreten ist,** setzen Sie ein Kreuz in das Kästchen unter dem Wort „nein". Die Spalten der Tabelle rechts daneben müssen dann nicht ausgefüllt werden. Lesen Sie die weiteren aufgelisteten Symptome und wiederholen Sie den Vorgang.

Wenn das Symptom aufgetreten ist, kreuzen Sie zuerst das Kästchen unter dem Wort „ja" an. Entscheiden Sie sich dann in der nächsten Spalte zwischen den Schweregraden der unerwünschten Ereignisse und tragen Sie **eine der Zahlen** in die Spalte ein.

In der daneben liegenden Spalte soll der Zeitpunkt eingetragen werden, an dem das Symptom bei dem Studiendurchgang aufgetreten ist. Dafür soll **eine der Zahlen** notiert werden. Danach bitten wir Sie die Dauer des Anhaltens des Symptoms in **Minutenangaben** anzugeben (beispielsweise 3 Minuten). Zudem soll eingetragen werden, ob Ihnen dieses Symptom bekannt ist und bei Ihnen schon **vor der Stimulation** aufgetreten ist, möglicherweise aufgrund einer Vorerkrankung (beispielsweise Kopfschmerzen bei der Vorerkrankung Migräne).

Liste der Symptome	Symptom vorhanden?<br>**Ja oder nein**<br>Wenn ja ➡	Schweregrad<br>**1, 2 oder 3**<br>1= mild<br>2= mittel / moderat<br>3= schwer ➡	Zeitpunkt<br>**0, 1, 2 oder 3**<br>0= Beginn<br>1= Mitte<br>2= Ende<br>3= nach der Studie ➡	Dauer<br>**in Minuten** ➡	Symptom bekannt?<br>**0 oder 1**<br>0= ja<br>1= nein
X	Nein ☐ Ja➡ ☐				

Liste der Symptome	Symptom vorhanden? **Ja oder nein** Wenn ja ➡	Schweregrad **1, 2 oder 3** 1= mild 2= mittel / moderat 3= schwer ➡	Zeitpunkt **0, 1, 2 oder 3** 0= Beginn 1= Mitte 2= Ende 3= nach der Studie ➡	Dauer **in Minuten** ➡	Symptom bekannt? **0 oder 1** 0= ja 1= nein
Hautrötung	Nein ☐ Ja➡ ☐				
Hautwunde	Nein ☐ Ja➡ ☐				
Kribbelgefühl an der Haut	Nein ☐ Ja➡ ☐				
Brennen an der Haut	Nein ☐ Ja➡ ☐				
Juckreiz an der Haut	Nein ☐ Ja➡ ☐				
Taubheitsgefühl der Haut	Nein ☐ Ja➡ ☐				
Druckgefühl an der Haut	Nein ☐ Ja➡ ☐				
Schmerzen an der Haut	Nein ☐ Ja➡ ☐				
Weiteres oder anderes unangenehmes Hautgefühl	Nein ☐ Ja➡ ☐				
Atem-beschwerden	Nein ☐ Ja➡ ☐				
Gefühl von Pulsveränderungen (Schwäche oder Herzrasen)	Nein ☐ Ja➡ ☐				
Schwindel	Nein ☐ Ja➡ ☐				

Liste der Symptome	Symptom vorhanden? **Ja oder nein** Wenn ja ➡	Schweregrad **1, 2 oder 3** 1= mild 2= mittel / moderat 3= schwer ➡	Zeitpunkt **0, 1, 2 oder 3** 0= Beginn 1= Mitte 2= Ende 3= nach der Studie ➡	Dauer **in Minuten** ➡	Symptom bekannt? **0 oder 1** 0= ja 1= nein
Muskelkrämpfe im Gesicht und/oder Nackens	Nein ☐ Ja➡ ☐				
Schluckbeschwerden	Nein ☐ Ja➡ ☐				
Kopfschmerzen	Nein ☐ Ja➡ ☐				
Nackenschmerzen	Nein ☐ Ja➡ ☐				
Brustschmerzen	Nein ☐ Ja➡ ☐				
Stimmstörung / Heiserkeit	Nein ☐ Ja➡ ☐				
Ohrbeschwerden (z.B. Völlegefühl, Druck)	Nein ☐ Ja➡ ☐				
Tinnitus / Ohrgeräusch	Nein ☐ Ja➡ ☐				
Übelkeit	Nein ☐ Ja➡ ☐				
Unwohlsein (unspezifisch)	Nein ☐ Ja➡ ☐				
Konzentrationsveränderungen	Nein ☐ Ja➡ ☐				
Müdigkeit	Nein ☐ Ja➡ ☐				

Frage 3

Wenn weitere Symptome während der Studie aufgetreten sind und diese nicht in Frage 2 abgefragt wurden, dann tragen Sie diese bitte in der linken Spalte der Tabelle ein. Füllen Sie dann bitte auch die weiteren Tabellenspalten nach dem in Frage 2 beschriebenen Schema aus.

Haben Sie weitere Symptome bemerkt? Wenn ja, diese bitte eintragen.	Schweregrad **1, 2 oder 3** 1= mild 2= moderat 3= schwer ➡	Zeitpunkt **0, 1, 2 oder 3** 0= Beginn 1= Mitte 2= Ende 3= nach der Studie ➡	Dauer **in Minuten** ➡	Symptom bekannt? **0 oder 1** 0= ja 1= nein

Frage 4

Wenn Symptome aufgetreten sind, dann geben Sie bitte eine Einschätzung dazu ab, welche Sie am meisten beeinträchtigt und gestört haben. Es können **maximal drei** Symptome angegeben werden.

Symptome			

Frage 5

Würden Sie eine weitere Vagusnervenstimulation im Kontext von einer Studienteilnahme durchführen lassen? Bitte kreuzen Sie entweder „ja“ **oder** „nein“ an.

☐ Ja ☐ Nein

Wenn Sie „nein“ angekreuzt haben, warum würden Sie keine weitere Stimulation durchführen lassen? (Grund bitte anführen)

____

____

Frage 6

Im Rahmen dieser Studie werden therapeutische, also wahre Stimulationen des Vagusnervs und auch Placebostimulationen (Scheinstimulationen) durchgeführt.
Schätzen Sie bitte ein, ob es sich bei diesen Studiendurchgang vermutlich um eine therapeutische Vagusnervstimulation **oder** um eine Placebostimulation gehandelt hat.
Kreuzen Sie **eines** der beiden Möglichkeiten an.

☐ Wahre Stimulation	☐ Placebo-Stimulation

Platz für weitere Bemerkungen:

Vielen Dank für die Beantwortung des Fragebogens!

Fragebogen für Testleiter

Während der Proband den Probandenfragebogen ausfüllt, bitte diesen Testleiterfragebogen auf Grundlage der Beobachtungen während der Studie ausfüllen.

Frage 1:

Ist der Fragebogen vollständig ausgefüllt worden?

☐ Ja ☐ Nein

Wenn „nein“ angekreuzt wurde, den Probanden darum bitten, diesen vollständig auszufüllen.

Frage 2:

Bitte geben Sie an, welche der Studienblöcke durchgeführt worden ist. Setzen Sie dafür ein Kreuz in die richtige Spalte in der Tabelle.

	Studiendurchgang
	**Placebo** Stimulation mit **30 Sekunden** Stromverabreichung
	**Placebo** Stimulation mit **3,4 Sekunden** Stromverabreichung
	**Therapeutische** Stimulation mit **30 Sekunden** Stromverabreichung
	**Therapeutische** Stimulation mit **3,4 Sekunden** Stromverabreichung

Frage 3:

Mit welcher Intensität wurde in diesem Studiendurchgang stimuliert?

_______ mA

Wurde die Intensität während des Durchgangs verändert? Wenn ja, wann?

Neue Intensität _______ mA, wann verändert: nach ________ min

Warum verändert:____

Frage 4:

Beschreiben der Beobachtungen bezüglich Symptome / unerwünschte Ereignisse:

____
____
____
____
____

Frage 5:

Kam es zu Symptomen, die den Gesundheitszustand des Probanden ernstzunehmend gefährdet haben? Bitte „ja“ **oder** „nein“ ankreuzen.

☐ Nein ☐ Ja

Wenn „ja“, welche und warum?

____
____
____
____
____
____

Frage 6:

Kam es zum Abbruch der Stimulation aufgrund von unerwünschten Ereignissen? Bitte „ja“ **oder** „nein“ ankreuzen.

☐ Ja ☐ Nein

Wenn „ja“, warum?

____
____
____
____
____
____

Hinweise:

- Siehe eigene Empfehlungen zur Auswertung des Fragebogens
- Siehe Checkliste für Empfehlungen zur Berichterstattung von Farmer et al. 2020
